# Photorelease of Hydrogen Sulfide (H_2_S) from Stable Water-Soluble Precursors

**DOI:** 10.1021/acs.joc.5c01997

**Published:** 2025-09-19

**Authors:** Kun Wang, Vladimir Popik

**Affiliations:** Department of Chemistry, University of Georgia, Athens, Georgia 30602, United States

## Abstract

Two hydrogen sulfide photoreleasing molecules (photo-HSRMs), 3-hydroxy-2-naphthalene-methanethiol and the corresponding disulfide, have been synthesized, and their properties and photochemical reactivity have been explored. Both compounds are stable in the neat form and in aqueous solutions in the dark but release H_2_S under exposure to UVA and UVB light in good quantum and chemical yields. In the presence of thiols, such as cysteine, glutathione, and mercaptoethanol, the yield of hydrogen sulfide release reaches 90%. The mechanism of H_2_S photorelease from these photo-HSRMs is discussed.

## Graphical abstract

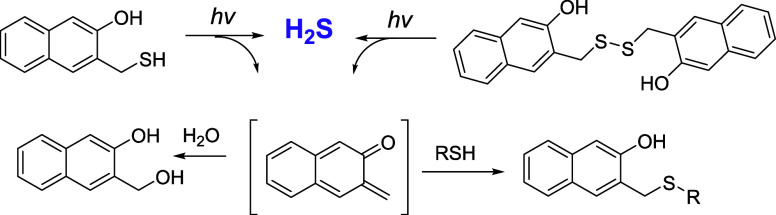

## Introduction

Hydrogen sulfide (H_2_S) is commonly known as a poisonous gas with the foul smell of rotten eggs. However, H_2_S was found to be a ubiquitous gasotransmitter, which is produced endogenously in mammals, albeit in very low concentrations.^1,2^ Hydrogen sulfide shows anti-inflammatory and antioxidative effects, offers cardioprotection under hypoxic conditions, shows antitumor properties, and is involved in ion channel regulation and many other processes.^3–5^ With this broad spectrum of biological functions, the development of selective H_2_S-releasing prodrugs has become an important goal of modern pharmacology.^6,7^ The systemic toxicity of hydrogen sulfide, however, prevents therapeutic use of inorganic donors, such as soluble sulfides and hydrosulfides.^8^ Due to the low acidity of H_2_S (p*K*_A1_ = 6.9), these salts undergo instant hydrolysis at biological pH, producing an equilibrium mixture of HS^–^ and H_2_S. Organic donors, on the other hand, can release hydrogen sulfide much slower, providing some control over its systemic concentration.^9^ For example, the biological activity of garlic-derived H_2_S donors, such as diallyl trisulfide (DATS or Allitridin) and diallyl disulfide (DADS), has been characterized both in vitro and in vivo*.* ^4,10^ Among synthetic hydrolysis-triggered H_2_S donors, 1,2-dithiole-3-thiones (DTTs), Lawesson’s reagent, and its analogs are extensively tested as adjuvants for anti-inflammatory and antitumor treatments.^11^ Light-directed generation of hydrogen sulfide potentially allows for further reduction of side effects by creating a relatively high concentration of the gasotransmitter in the target tissues while sparing the rest of the system from its toxicity.^4,12^

Three general approaches have been developed to achieve the photorelease of hydrogen sulfide. The first strategy relies on caging of both acidic hydrogens in hydrogen sulfide^13,14^ with photolabile protecting groups (PPGs, Scheme 1A). The sequential cleavage of both PPGs releases H_2_S.^13,14^ The second tactic employs the photogeneration of gem-dithiols^15,16^ or thioaldehydes,^17^ which produce hydrogen sulfide upon hydrolysis (Scheme 1B). The third method is based on the use of caged thiocarbamates.^18–20^ Thiocarbamates, which are released upon irradiation, undergo “decarbonylation,” forming the carbonyl sulfide (O=C=S). The conversion of the hydrated form of the latter (H_2_CO_2_S) into H_2_S and CO_2_ is catalyzed by carbonic anhydrase (Scheme 1C).^18–20^ In addition, a three-component system has been reported.^21^ It employs Diels–Alder addition of singlet oxygen, generated in triplet sensitizer (PS)-mediated photolysis, to 1,3-diphenylisobenzothiophene, with a subsequent reductive release of hydrogen sulfide (Scheme 1D).

**Scheme 1:**
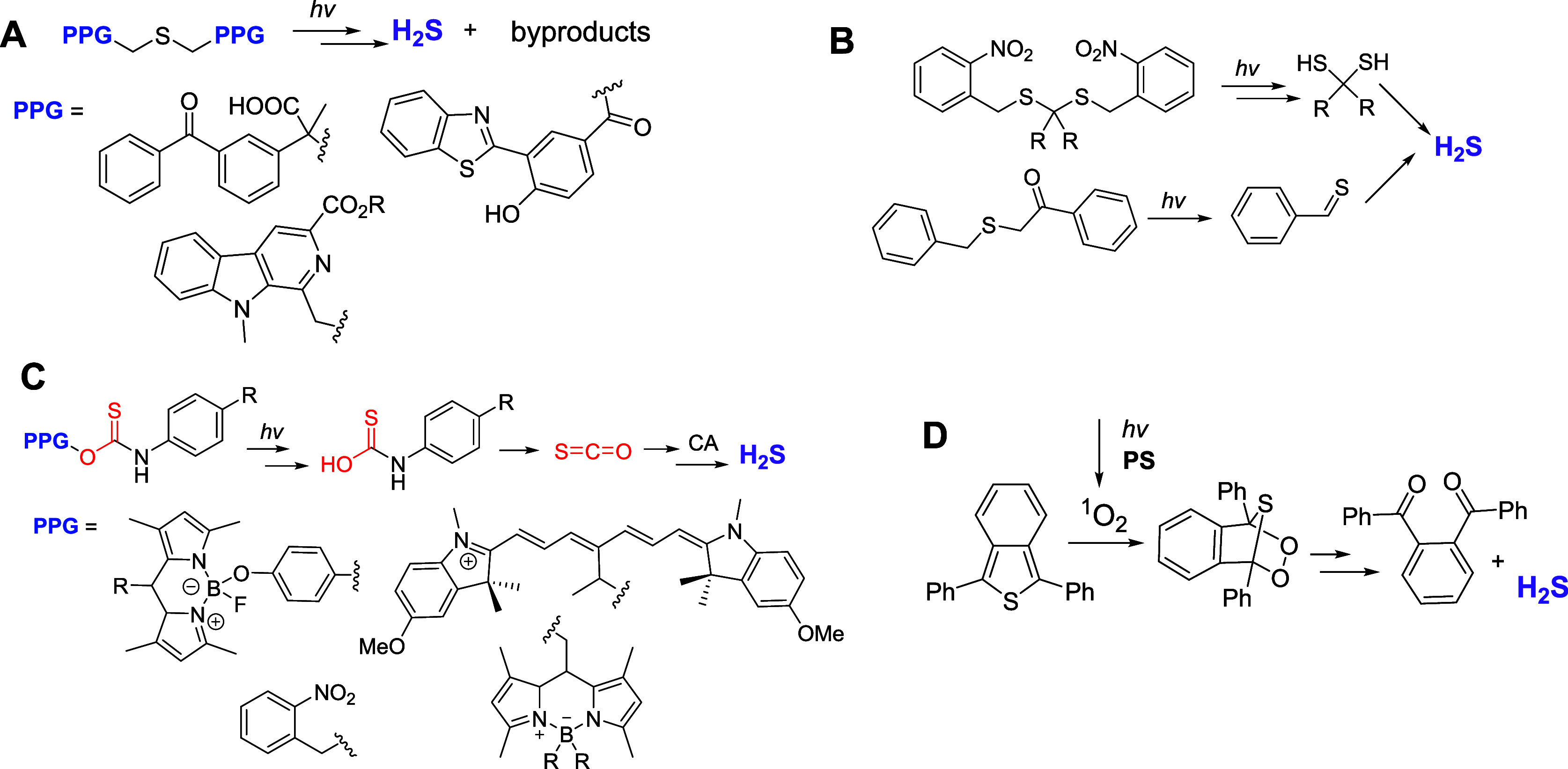
Light-Induced Release of Hydrogen Sulfide

In this report, we discuss the utility of 3-hydroxy-2-naphthalenemethyl PPG for the rapid single-step release of hydrogen sulfide. 3-Hydroxy-2-naphthalenemethyl-caged substrates are released within ∼12 μs upon excitation, producing o-naphthoquinone methide (oNQM) as a byproduct.^22,23^ In aqueous solutions, the latter undergoes rapid hydration to 3-hydroxy-2-naphthalenemethanol (NQMP, for NaphthoQuinone Methide Precursor). Thioethers of NQMP (NQMP-SR) have been shown to release thiols in good quantum and chemical yields upon UVA irradiation (Scheme 2).^24,25^ The reaction is reversible, as NQM can add thiols to regenerate the starting material (NQMP-SR).^25^ However, in aqueous solution, under appropriate conditions (pH ≪ pKA of RSH and [NQMP-SR] < 0.4 mM), the hydration of oNQM to NQMP is faster than the return reaction (Scheme 2).^25^ Alternatively, the oNQM intermediate can be intercepted by another nucleophile (e.g., glutathione)^24,25^ or by IEDDA cycloaddition to electron-rich alkenes (e.g., vinyl ethers or enamines).^24–27^

**Scheme 2:**
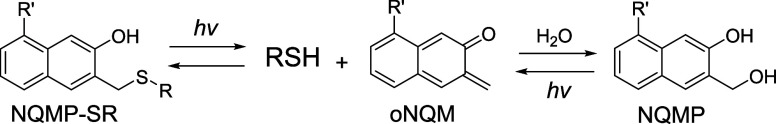
Reversible Photocleavage of (3-Hydroxy-naphthalen-2-yl)­methyl Thioethers (NQMP-SR)

The substituent R′ in the 8-position of NQMP can be employed for the modulation of solubility (e.g., R′= −(CH_2_CH_2_O)_*n*_-X), attachment of additional functionalities (e.g., fluorophore, biotin, cyclooctyne, azide, amine, carbohydrate, etc.), or modification of photophysical properties.^24,25,28^

Here, we report preparation, properties, and photoreactivity of two novel water-soluble H_2_S photodonors: 3-(mercaptomethyl)-2-naphthol (NQMP-SH, **1**) and the corresponding disulfide (**2**, DNQMP-S, Scheme 3).^29^ Upon UVA irradiation of NQMP-SH (**1**) and DNQMP-S (**2**) in aqueous solutions at biological pH, hydrogen sulfide is released with excellent quantum efficiency in good chemical yield. oNQM, which is formed in this reaction, is rapidly hydrated^23^ or is quenched by an appropriate nucleophile (Scheme 3).

**Scheme 3:**
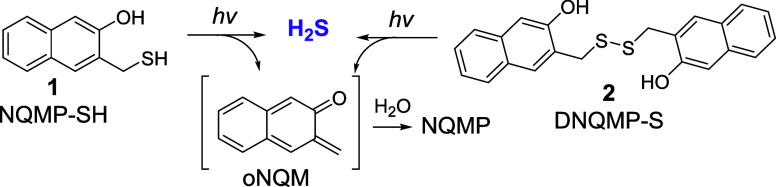
Photorelease of Hydrogen Sulfide from NQMP-SH (**1**) and DNQMP-S (**2**)

## Results and Discussion

Two synthetic approaches to NQMP-SH (**1**) and DNQMP-S (**2**) have been explored. In the first method, NQMP diacetate **3** has been prepared by acylation of NQMP^23^ with acetic anhydride in the presence of pyridine. The reaction of diacetate **3** with sodium hydrosulfide in aqueous acetonitrile (1:4) or DMF produced the target DNQMP-S (**2**) in a moderate yield. The reduction of disulfide **2** with LAH in THF quantitatively converts **2** to the target NQMP-SH (**1**, Scheme 4).

**Scheme 4:**
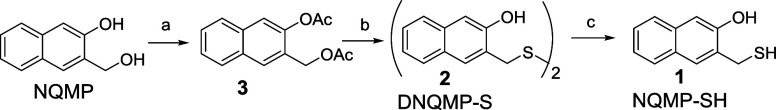
Synthesis of NQMP-SH (**1**) and DNQMP-S (**2**)­^a^

a Reagents and conditions: (a) acetic anhydride, pyridine quant.; (b) NaSH, aqueous acetonitrile (65%) or DMF (34%); and (c) LAH, THF, quant.

The alternative approach to thiol **1** via the addition of thiourea to benzylic bromide with subsequent basic hydrolysis requires the protection of phenolic hydroxyl due to the instability of 3-(bromomethyl)-2-naphthol (3-(bromomethyl)-2-naphthol rapidly loses HBr to produce oNQM with subsequent oligomerization and other reactions.) Therefore, both hydroxy groups in NQMP were converted to TBDMS ethers under conventional conditions to give **4**. The benzylic protection in **4** has been selectively removed by hydrofluoric acid in acetonitrile, yielding mono-TBDMS-protected NQMP **5**, which, in turn, was treated by PBr_3_ to give the desired benzylic bromide **6** in a good yield. The reaction of **6** with thiourea, followed by basic hydrolysis, produced a 3:1 mixture of NQMP-SH (**1**) and DNQMP-S (**2**) with a combined yield of 81%. It should be noted that at longer reaction times, the yield of sulfide **2** grows at the expense of **1**. A byproduct, 3-hydroxy-2-naphthaldehyde (**13**, 17%), has been isolated at this step. The treatment of the reaction mixture with LAH produced NQMP-SH (**1**) in 80% overall yield (Scheme 5). It is interesting to note that disulfide **2** is surprisingly unreactive toward common disulfide reducing reagents, such as TCEP, LiCl/NaBH_4_, Mg, and LTBA.

**Scheme 5:**
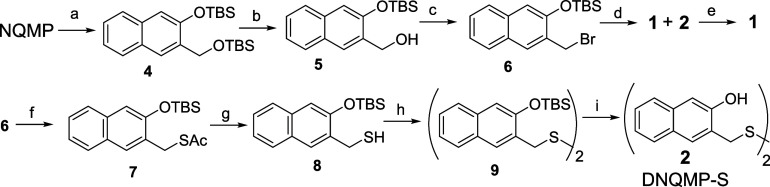
Synthesis of NQMP-SH (**1**) and DNQMP-S (**2**)­^a^

a Reagents and conditions: (a) TBDMS chloride, imidazole, DMAP, DMF, quant.; (b) HF, aqueous acetonitrile; 80%; (c) PBr_3_, ether; 83%; (d) *i*) thiourea, EtOH; *ii*) NaOH, **1** (54%), **2** (27%); (e) LAH, THF; quant.; (f) potassium thioacetate, DMF, 76%; (g) NaOMe, MeOH, 65%; (h) H_2_O_2_, quant.; and (i) TBAF, THF, 17%.

The red-ox interconversion of NQMP-SH (**1**) and DNQMP-S (**2**) was found to be a clean reaction; hence, disulfide **2** has also been prepared by reacting benzylic bromide **6** with thioacetate to give thioester **7**, which was saponified by sodium methoxide in MeOH to TBDMS-protected NQMP-SH (**8**). Hydrogen peroxide oxidation of the latter to disulfide **2**, followed by deprotection, produced the target DNQMP-S (**2**, Scheme 5). NQMP-SH (**1**) is a colorless crystalline compound, while DNQMP-S (**2**) is a yellow solid. Both compounds are stable under ambient conditions in the dark and are soluble in water up to ca. 100 μM concentration. Aqueous solutions of **1** slowly accumulate disulfide **2** over time, while DNQMP-S (**2**) solutions are stable in the dark for over a month.

### Photorelease of Hydrogen Sulfide from NQMP-SH (1)

The UV spectrum of compound **1** is presented in the Supporting Information.^31^ The photolyses were conducted either in aqueous biphosphate buffer (PB, pH= 7.4) or in a 1:1 acetonitrile–biphosphate buffer solution (ACN:PB, pH= 7.4). The latter medium has been employed to avoid turbidity in the photolyzates caused by the formation of insoluble byproducts. The quantification of H_2_S release in photolyses of NQMP-SH (**1**) and DNQMP-S (**2**) was conducted using the methylene blue (MB) assay.^30,31^ Upon 350 nm irradiation of a 0.50 mM NQMP-SH (**1**) in ACN:PB, the concentration of photoreleased hydrogen sulfide rapidly grew during the first 5 min but plateaued at ∼0.018 mM (36%, Figure 1).^31^

**Figure 1:**
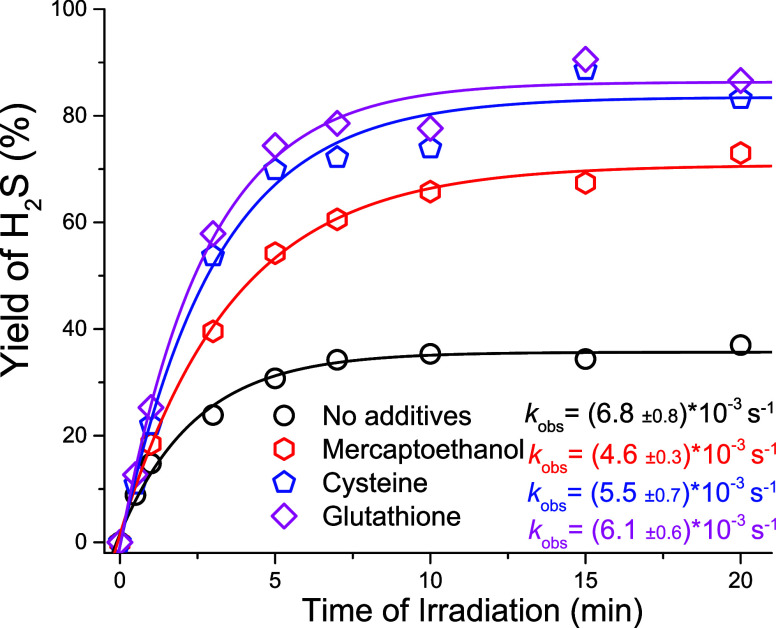
Yield of H_2_S formed under 350 nm irradiation of a 0.50 mM NQMP-SH **1** solution in ACN:PB (black circles) and in the presence of 10 mM 2-mercaptoethanol (red hexagons), cysteine (blue pentagons), and glutathione (magenta squares). The apparent rate constants of the H_2_S release were obtained by fitting the data to single exponential equation. Each data point represents the averaged results of three independent runs.

Incubation of the same NQMP-SH (**1**) solution in the dark for 2 h produced no detectable levels of hydrogen sulfide or consumption of the starting material. The moderate yield of hydrogen sulfide in this experiment can be explained by achieving the steady-state conditions due to photodriven equilibrium between NQMP-SH (**1**) and NQMP, similar to one that has been observed previously in thiol photorelease studies.^25^ oNQM formed upon the photorelease of H_2_S from **1** can be hydrated to give NQMP or react with hydrogen sulfide to produce the starting material (**1**). NQMP, on the other hand, can also undergo photodehydration to oNQM^23^ (Scheme 6).

**Scheme 6:**
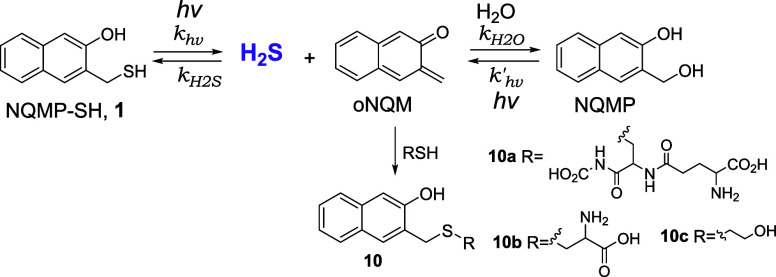
Generation of Hydrogen Sulfide from NQMP-SH (**1**) in Aqueous Solutions Alone or in the Presence of Thiols (Glutathione, Cysteine, or 2-Mercaptoethanol)

The position of this equilibrium is defined by the rate of reaction of the common intermediate oNQM with water (*k*_H2O_[H_2_O]) and H_2_S (*k*_H2S_[H_2_S]), as well as by the rate of the photoelimination reaction of NQMP-SH (**1**, *k*_*hv*_[NQMP-SH]) and NQMP (*k*′_*hv*_[NQMP]). Since, under the established equilibrium conditions, the concentrations of **1** and NQMP remain constant, we can write the following equation (eq 1), where [NQMP], [H_2_S], and [NQMP-SH] are equilibrium concentrations of the corresponding substrates:

$$\frac{k_{hv}[\mathrm{NQMP}-\mathrm{SH}]}{k_{H2S}[H_{2}S]}=\frac{k_{hv}^{\prime }[\mathrm{NQMP}]}{k_{H2O}[H_{2}O]}$$ 1

$$[H_{2}S]=\frac{k_{hv}[\mathrm{NQMP}-\mathrm{SH}]k_{H2O}[H_{2}O]}{k_{hv}^{\prime }[\mathrm{NQMP}]k_{H2S}}$$ 2

Assuming that Scheme 6 accurately describes the process, the equilibrium concentration of hydrogen sulfide can be described by eq 2. Since UV spectra of NQMP and **1** are almost identical (same chromophore), in dilute solutions, we can equate the ratio of photochemical rate constants to the ratio of quantum yields (eq 3). Therefore, the rate of the oNQM reaction with H_2_S (*k*_H2S_) can be evaluated using eq 4:

$$\frac{k_{hv}}{k_{hv}^{\prime }}=\frac{\Phi_{1}}{\Phi_{\mathrm{NQMP}}}$$ 3

$$k_{H2S}=\frac{\phi_{1}[\mathrm{NQMP}-\mathrm{SH}]k_{H2O}[H_{2}O]}{\phi_{\mathrm{NQMP}}[\mathrm{NQMP}][H_{2}S]}$$ 4

Using the previously determined values for the rate of oNQM hydration in aqueous solutions (pH = 3–9), *k*_H2O_ = 2.61 M^–1^ s^–1,^ ^23^ quantum yields Φ_**1**_= 0.1,^26^ Φ_NQMP_ = 0.2,^26^ and experimental [H_2_S] = [NQMP] = 0.18 mM, and [NQMP-SH] = 0.32 mM (Figure 1), we can estimate the rate of the oNQM reaction with hydrogen sulfide at pH = 7.4 at *k*_H2S_ ∼ 4 × 10^5^ M^–1^ s^–1^. There is also a possibility that the yield of H_2_S was somewhat reduced due to oxidation; however, even at 30 min photolyses, we have not observed a significant reduction in hydrogen sulfide yield.

The introduction of competing nucleophiles, which could intercept the oNQM intermediate, by trapping it as sulfides **10**, should result in the enhancement of H_2_S yield (Scheme 9). Since the cytosol of mammalian cells contains ca. 10 mM glutathione (GSH), photolysis of NQMP-SH (**1**) was conducted in the presence of 10 mM GSH (Figure 1). Under these conditions, the yield of H_2_S photorelease has reached 90% (Figure 1). The irradiation of NQMP-SH (**1**) in the presence of 10 mM of cysteine and 2-mercaptoethanol (2-ME) also resulted in the enhancement of H_2_S release. The concentration of H_2_S formed after 15 min of 300 nm irradiation of a 0.50 mM NQMP-SH (**1**) solution in the presence of GSH was 0.45 mM (90%), the concentration of cysteine was 0.44 mM (88%), and the concentration of 2-ME was 0.36 mM (74%) (Figure 1).^31^ The lower yield of H_2_S release in the presence of mercaptoethanol is, probably, explained by the lower acidity (p*K*_a_ = 9.72, while GSH p*K*_a_= 8.56 and l-cysteine p*K*_a_= 8.4), which translates in lower concentration of the reactive thiolate species and a slower oNQM quenching rate. Irradiation of 10 mM solutions of GSH, cysteine, and 2-ME with 300 nm light for 15 min or 15 min incubation of NQMP-SH (**1**) with thiols did not produce detectable levels of hydrogen sulfide.

Product studies of NQMP-SH (**1**) photolyses were conducted by HPLC. The identity of the photoproducts was confirmed by coinjection with the authentic samples. Compounds **10c** and **11** were isolated in preparative photolyses, aldehyde **13** was produced as a byproduct in NQMP-SH (**1**) synthesis, and thioether **12** was independently synthesized.^31^

Under 300 nm irradiation, photoconversion of **1** is 15–20 times faster due to the higher extinction coefficient of the substrate at this wavelength. After 30 s of irradiation of **1** (0.125 mM) in PB, the photolysate contained NQMP-SH (**1**, ∼38 μM), NQMP (∼17 μM), DNQMP-S (**2**, ∼19 μM), bis-NQMP-thioether (**12**, ∼4 μM), and 3-methyl-2-naphtol (**11**, ∼18 μM, Scheme 7). The reaction mixture of the exhaustive photolysis (8 min) contains only NQMP (∼90 μM), **11** (∼12 μM), and 3-hydroxy-2-naphtaldehyde (**13**, 2–5 μM). 3-Methyl-2-naphtol (**10**) and 3-hydroxy-2-naphtaldehyde (**13**) are apparent products of the disproportionation of NQMP. In fact, exhaustive photolyses of either **1** or **2** in the presence of thiols (acting as reducing agents) result in high yields of **11** (*vide infra*). Sulfide **12** is an apparent product of the reaction of the oNQM intermediate with the starting material.

**Scheme 7:**
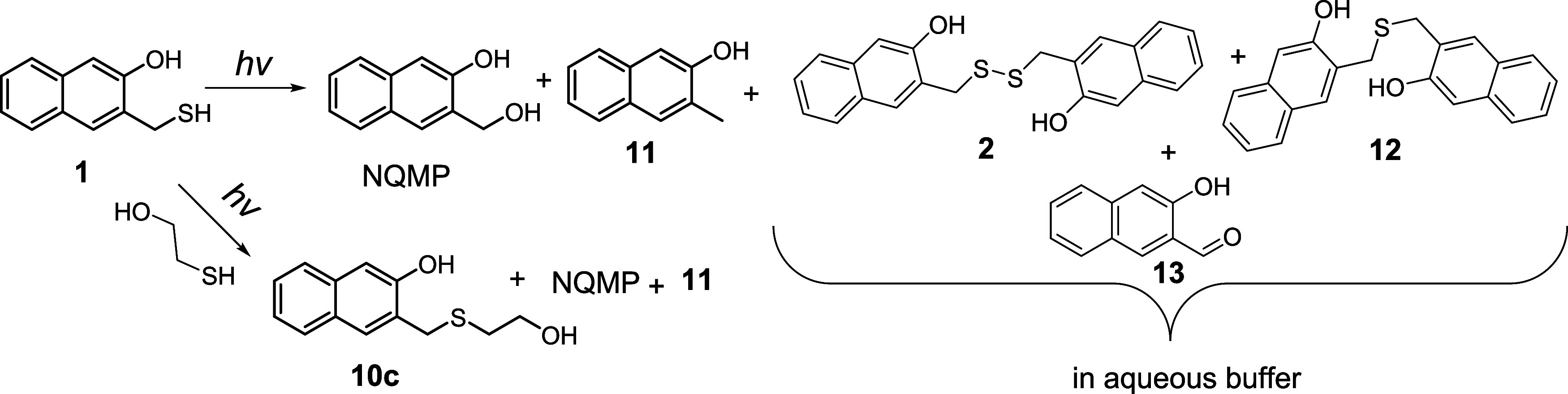
Irradiation of NQMP-SH (**1**) in Aqueous Biphosphate Buffer (pH = 7.4), Acetonitrile–Biphosphate Buffer Solution Alone, or in the Presence of Mercaptoethanol (2-ME)

In contrast, the irradiation of 500 μM of **1** in ACN:BP resulted in a much cleaner reaction: after 1 min of irradiation (longer exposure is required to accommodate higher concentrations), we observed only three prominent peaks: NQMP-SH (**1**, ∼260 μM), NQMP (∼280 μM), and some unknown highly polar intermediates. DNQMP-S (**2**) and thioether (**12**) were not detected. The starting thiol **1** was completely consumed after 7 min of irradiation, producing two products: NQMP (**1**, ∼320 μM) and **11** (∼130 μM, Scheme 7).

Photolysis of **1** in ACN:BP in the presence of 10 mM 2-ME initially produces a mixture of 3-(((2-hydroxyethyl)­thio)­methyl)­naphthalen-2-ol (**10c**), an apparent product of oNQM trapping by 2-ME, NQMP, and **11** (Scheme 7). Upon exhaustive irradiation, only **11** was detected in the photolysate. In the presence of 10 mM glutathione (GSH), only NQMP and a mixture of GSH-NQM adducts (**10a**) (identified only by mass spectra) were detected at ∼30% photolysis conversion. Longer irradiation leading to the consumption of NQMP-SH (**1**) yields **11** and traces of aldehyde **13**.

### Photorelease of Hydrogen Sulfide from DNQMP-S (**2**)

The 350 nm irradiation of DNQMP-S (**2**, 0.25 mM) in ACN:PB produced a low yield of hydrogen sulfide, barely reaching 11% (Figure 2). This was an expected outcome since the sequential removal of both photolabile NQMP-protecting groups should produce hydrogen persulfide, H_2_S_2_. The latter is known to undergo disproportionation in aqueous solutions to give H_2_S and elemental sulfur, among other transformations.^32^ The addition of thiols to the solution of **2** before irradiation should not only help trap the intermediate oNQM but also enhance the rate of hydrogen sulfide release by sulfide exchange with the initially produced persulfide NQMP-SSH (**14**, Scheme 8). Subsequent photocleavage of disulfide **15** is expected to produce persulfide **16**, which can undergo another sulfide exchange with the excess of thiol. Hydrogen persulfide, which can be formed by the subsequent uncaging from **14** (3-hydroxy-naphthalen-2-yl)­methylsulfenothioic acid (14) was prepared as a reference for product studies^31^), is also expected to produce H_2_S and **16**. Thus, the photolysis of DNQMP-S (**2**) in the presence of thiols is expected to release 2 equivalents of hydrogen sulfide (Scheme 8).

**Scheme 8:**
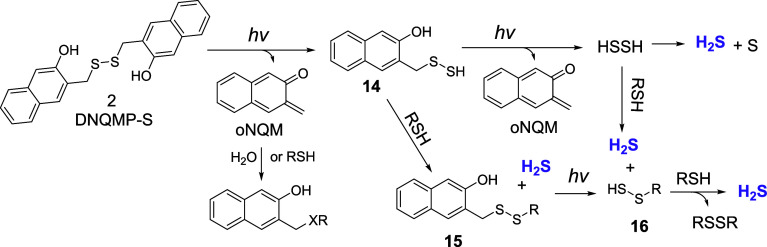
Irradiation of DNQMP-S (**2**) in Acetonitrile–Biphosphate Buffer Solution Alone or in the Presence of Thiols

**Figure 2:**
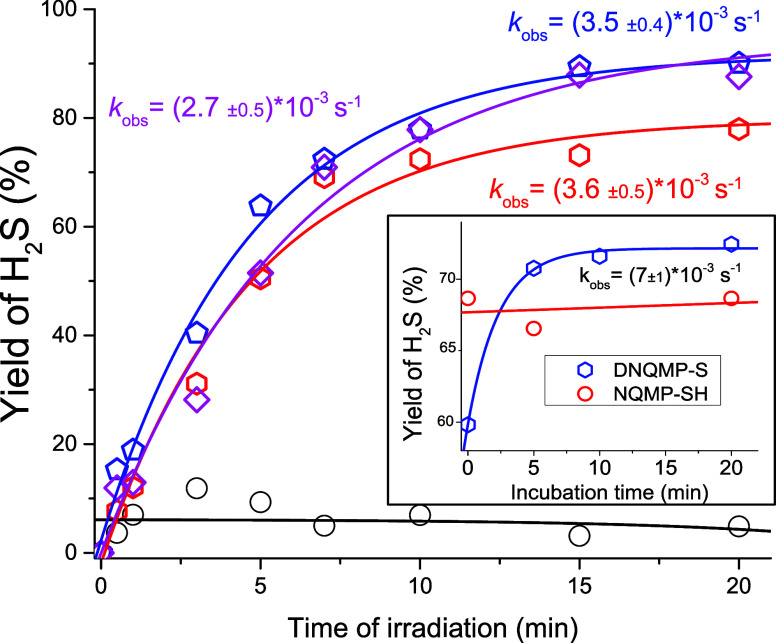
Yield of H_2_S formed in 350 nm photolyses of 0.25 mM disulfide **2** in ACN:BP (1:1) (black circles) and in the presence of 10 mM 2-mercaptoethanol (red hexagons), cysteine (blue pentagons), and glutathione (magenta squares). The apparent rate constants of the H_2_S release were obtained by fitting the data to a single exponential equation. The inset illustrates the delayed H_2_S release after the incomplete photolysis of **2** in the presence of 10 mM cysteine (blue pentagons). Analogous data for **1** (red circles) are plotted for the comparison. Each data point represents the averaged results of three independent runs.

In fact, the concentration of H_2_S formed after 20 min of 350 nm irradiation of a 250 μM solution of disulfide **2** in ACN:PB in the presence of glutathione was 440 μM (88% of theoretical yield), in the presence of cysteine was 450 μM (90%), and in the presence of mercaptoethanol was 390 μM (78%, Figure 2). No significant formation of hydrogen sulfide has been observed after 2 h of incubation of the above solutions in the dark.

The mechanism of the hydrogen sulfide release in the irradiation of DNQMP-S (**2**) in the presence of thiols suggests that the actual formation of H_2_S occurs in the dark reaction after the photochemical step (Scheme 8). In fact, shorter (<20 min *vide supra*) irradiation of **2** in the presence of cysteine, followed by incubation in the dark, demonstrated the delayed release of hydrogen sulfide. Thus, the incubation of the photolysate of **2** after 1 min of irradiation in the dark for 20 min doubles the initial yield of H_2_S (from 14 to 27%).^31^ After 5 min of irradiation, the yield of H_2_S is ∼60% and it grows in the dark in an exponential fashion, saturating at ∼74% at 20 min (insert in Figure 2). The apparent rate of the dark reaction is 0.007 ± 0.001 s^–1^ at a starting concentration of DNQMP-S (**2**) of 250 μM and r.t. Such delayed hydrogen sulfide release has not been observed in NQMP-SH (**1**) photolyses, e.g., 10 min of 300 nm irradiation of **1** (500 μM in ACN:PB) in the presence of cysteine produces 340 μM of H_2_S. The concentration of H_2_S has not increased after 5 and 20 min of incubation in the dark (inset in Figure 2).

The photorelease of hydrogen persulfide from NQMP-SSH (**14**) is currently under investigation.

Product studies of DNQMP-S (**2**) 300 nm photolysis were conducted by HPLC. The pattern of byproduct formation was similar to the photolyses of NQMP-SH (**1**). After 30 s irradiation of **2** (63 μM) in ACN:PB, ∼75% conversion has been achieved. The photolysate contained NQMP (∼30 μM), bis-NQMP-thioether (**12**, ∼17 μM), 3-methyl-2-naphtol (**11**, ∼8 μM), and 3-hydroxy-2-naphtaldehyde (**13**, ∼18 μM, Scheme 9). After 4 min of irradiation, a complete consumption of **2** has been observed, producing NQMP (∼56 μM), **11** (∼39 μM), and **13** (∼17 μM). Further exposure of the reaction mixture to 300 nm light resulted in a slow increase of concentration of **11** at the expense of NQMP, while the amount of aldehyde **13** was practically unchanged.

**Scheme 9:**
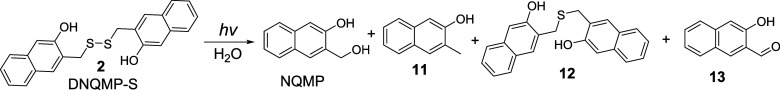
Products Detected in the Photolysate of DNQMP-S (**2**) in Acetonitrile–Biphosphate Buffer Solution

HPLC analysis of a solution of disulfide **2** and 2-ME in ACN:BP indicated the presence of significant amounts of NQMP-SH (**1**) and 3-(((2-hydroxyethyl)­disulfaneyl)­methyl)-naphthalen-2-ol (**15c**), an apparent product of disulfide exchange between **2** and 2-ME (Scheme 10). At ambient temperature and neutral pH, the half-lifetime of DNQMP-S (**2**) in the presence of 10 mM 2-ME is ca. 30 min. It took 3 h for the complete consumption of **2**. Overnight incubation resulted in the quantitative formation of NQMP-SH (**1**).

**Scheme 10:**
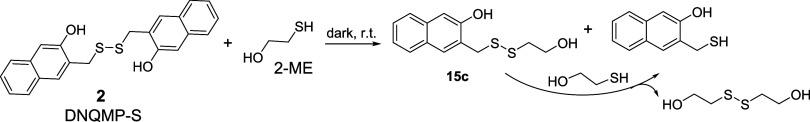
Dark Reaction of DNQMP-S (**2**) with 2-ME

Thirty minutes of 300 nm irradiation of a freshly prepared solution of 0.125 mM disulfide **2** and 10 mM 2-ME in ACN:BP resulted in complete consumption of the starting material and the formation of 3-methyl-2-naphtol (**11**, ∼0.2 mM) as the main product, as well as minor quantities (<20 μM) of NQMP and thioether **10c** (Scheme 11). Similar results were obtained in the photolysis of **2** in the presence of glutathione.

**Scheme 11:**
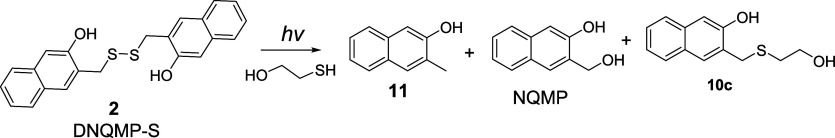
Products Detected in the Photolysate of DNQMP-S (**2**) in Acetonitrile–Biphosphate Buffer Solution in the Presence of 2-ME

To support the participation of oNQM in the photochemical reactions of NQMP-SH (**1**) and disulfide **2**, these compounds were irradiated in ACN:BP in the presence of ethyl vinyl ether (EVE). HPLC analysis of the photolysate, using an independently prepared sample of 2-ethoxy-3,4-dihydro-2*H*-benzo­[*g*]­chromene (**21**),^31^ allows for detection of the formation of **21**, the apparent product of a Diels–Alder addition of EVE to oNQM (Scheme 12).

**Scheme 12:**
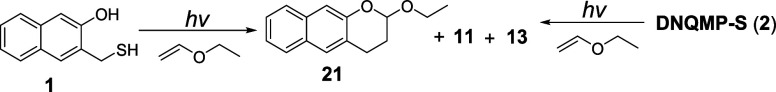
Photolysis on NQMP-SH (**1**) and DNMP-S (**2**) in the Presence of Ethyl Vinyl Ether

At low conversion of the starting materials, the adduct **21** is the major product, however, the exhaustive photolysis of NQMP-SH (**1**) and DNMP-S (**2**) in the presence of a 200-fold excess of EVE produced ∼20% yield of **21**, along with 3-hydroxy-2-naphthaldehyde (**13**) and 3-methyl-2-naphthol (**11**, Scheme 12).

## Conclusions

Two water-soluble photoactivated hydrogen-sulfide-releasing molecules (photo-HSRM), thiol NQMP-SH (**1**) and disulfide DNQMP-S (**2**), were synthesized, and their photochemistry was studied in detail. Both compounds have long shelf-life and good stability in solutions in the dark. Thiol **1** and disulfide **2** are readily interchangeable via reduction/oxidation reactions. Irradiation of NQMP-SH (**1**) and DNQMP-S (**2**) in aqueous solutions at pH = 7.4 without any additives results in the release of hydrogen sulfide in 30% and 11% chemical yields, correspondingly. In the presence of thiols (mercaptoethanol, cysteine, and glutathione), the yield of H_2_S reaches 90%. DNQMP-S (**2**) is capable of releasing 2 equivalents of hydrogen sulfide.

Disulfide DNQMP-S (**2**) and persulfide NQMP-SSH (**14**) are currently studied as potential precursors for the photorelease of hydrogen persulfide H_2_S_2_.

## Supplementary Material



## Data Availability

The data underlying this study are available in the published article and its Supporting Information.

## Notes

General information on experimental conditions, photochemical setup, quantification of H_2_S release, synthesis procedures, UV spectrum of compound **1**, and NMR spectra (PDF)

## 1

Frontage Laboratories, Inc., Exton, PA 19341, United States
