# Supplementary material for: Photorelease of Hydrogen Sulfide (H2S) from Stable Water-Soluble Precursors
Source: J Org Chem. 2025 Sep 19;90(39):13938–44. doi: 10.1021/acs.joc.5c01997 (PMC12501947; doi:10.1021/acs.joc.5c01997)

Supporting Information

Photo-release of hydrogen sulfide (H_2S) from stable water-soluble precursors

Kun Wang, Vladimir Popik*

Department of Chemistry, University of Georgia, GA 30602, USA

vpopik@uga.edu

Table of contents:

General Information……..……………………..…………………...………………………………S2

Quantification of H_2S Release…………………..…………………..……………………………. S2

Materials……………......………………………..…………………..………………………………S3

UV Spectrum of NQMP-SH……………………..………………………………………………..S11

^{1}H and ^{13}C NMR spectra……………………..………………………………………………..S13

General Information. All organic solvents were dried and freshly distilled before use. Flash chromatography was performed using 40-63 μm. Anhydrous sodium sulfate has been employed for drying in work-up procedures. All NMR spectra were recorded in $CDCl_3$ using 400 MHz instrument and chemical shift were referenced to TMS unless otherwise noted. Solutions for photochemical reactions were prepared using HPLC grade water and acetonitrile.

Photochemical reactions. Analytical photolyses were conducted using standard 1X1X3 cm quartz fluorescent cuvette suspended in the center of the Rayonet RPR-600 Photochemical Reactor (ca. 60 mm from the lamps) equipped with 8 (4 W) fluorescent UV lamps (254, 300 or 350 nm).[1] For the preparative reactions, sample solutions were placed in the quartz test tube (16X180 mm) equipped with a stirrer and stopper. The tube was suspended in the center of the Rayonet RPR-100 reactor (ca. mm from the lamps) equipped with 16 (18 W) fluorescent UV lamps (254, 300 or 350 nm).[1] .

Product studies were conducted by HPLC using authentic samples of the photoproducts for calibration. The latter were either isolated in preparative photolyses or independently synthesized (*vide infra*).

Quantification of H_2S Release was conducted using Methylene Blue test.[2] Aqueous solution of $FeCl_3$, *N,N*-dimethyl-p-phenylenediamine sulfate, and zinc acetate was added to photolysates and incubated for 15 min. In the presence of H_2S Methylene Blues is formed and its yield is proportional to H_2S concentration. The intensity of the characteristic absorbance band of the former at 663 nm was employed for H_2S quantification. Solutions of sodium sulfide in the appropriate medium were employed for calibration (Figure S1).

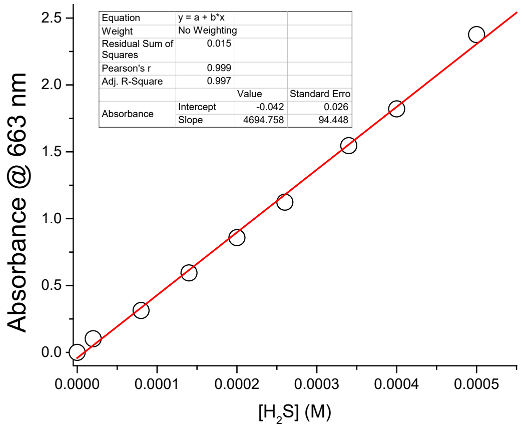

Figure S1. Absorbance of Methylene Blue at 663 nm in 1:1 acetonitrile – biphosphate buffer solution (pH= 7.4) of sodium sulfide.

Materials: All commercially available reagents were used without further purification. 3-hydroxy-naphthalenemethanol (NQMP),[3] 3-(acetoxymethyl)naphthalen-2-yl acetate (**3**)[4] and 2-(bromo-methyl)-3-(ethoxymethoxy)naphthalene[5] have been prepared as previously reported in the literature.

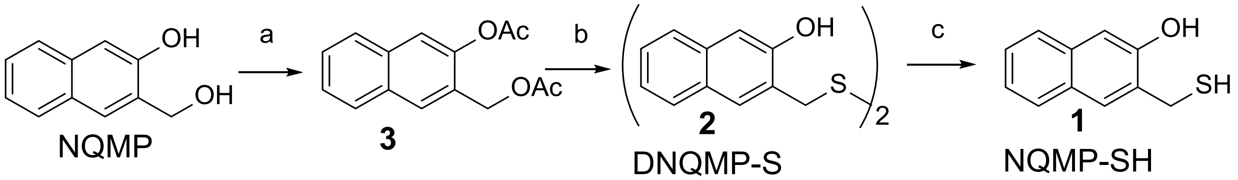

3-(acetoxymethyl)naphthalen-2-yl acetate (**3**).[4] Acetic anhydride (7.1 ml) was added to a solution of NQMP (3.2 g, 18.3 mmol) in pyridine (7.3 ml). The reaction mixture was stirred under room temperature for 2 h, solvent removed in vacuum, the residual solid was re-dissolved with CH_2Cl_2, washed with 1M HCl, water, brine, and dried. The solvent was removed in vacuum give the NMR-pure **3** (4.0 g, quant.) as a pale-yellow solid.

^{1}H NMR δ 7.91 (s, 1H), 7.85 – 7.78 (m, 2H), 7.57 (s, 1H), 7.51-7.45 (m, 2H), 5.24 (s, 2H), 2.37 (s, 3H), 2.10 (s, 3H).

^{13}C{^{1}H} NMR δ 170.7, 169.6, 146.8, 133.7, 131.3, 130.2, 127.9, 127.4, 127.2, 127.0, 126.14, 120.1, 62.0, 21.97, 20.92.

3-(mercaptomethyl)naphthalen-2-ol, NQMP-SH (**1**). NaSH (2.25 g, 40 mmol) was added to a solution of compound **3** (2 g, 7.7 mmol) in 50 mL of acetonitrile : water (4:1). The reaction mixture was stirred at 40 ^{0}C (using oil bath) for 1 h, acetonitrile removed in vacuum, and the product extracted with DCM, dried, solvent removed in vacuum to give the yellowish oil containing mixture of compounds **2** and **1** (1.16 g, 65%), where disulfide **2** was the major component. It was used in the next step without purification.

The solution of the above mixture (0.1 g ca. 022 mmol) in THF (1 mL) was added dropwise to a stirred solution of LAH (0.72 mmol) in THF (1 mL). The reaction mixture was stirred at room temperature for 30 min, quenched by adding water and 1M HCl dropwise, extracted by ethyl acetate. The organic layer was washed with brine, dried over Na_2SO_4, filtered, and purified by chromatography (ethyl acetate: hexanes 1:3) to afford the product (82 mg, 100%) as colorless crystals.

^{1}H NMR δ 7.67 (d, *J* = 8.1 Hz, 1H), 7.57 (d, *J* = 12.0 Hz, 1H), 7.57 (s, 1H), 7.40 – 7.33 (m, 1H), 7.29 (td, *J* = 7.5, 6.9, 1.4 Hz, 1H), 7.08 (s,1H), 6.19 (s, 1H), 3.86 (d, *J* = 7.5 Hz, 2H), 1.97 (td, *J* = 7.6, 1.3 Hz, 1H).

$^{13}C\{^1H\}$ NMR δ 151.9, 134.0, 129.1, 128.9, 128.3, 127.4, 126.3, 126.0, 123.9, 110.9, 25.2.

IR (neat) cm^{-1}: 3321 vs, br (O-H); 3050 w (C_{Ar}-H); 2536 m (S-H); 1608 s, 1513 s (Ar).

HRMS (ESI/ Orbitrap), *m/z*: $[M-H]^-$ Calcd for $C_{11}H_9OS$ 189.0380; Found 189.0379.

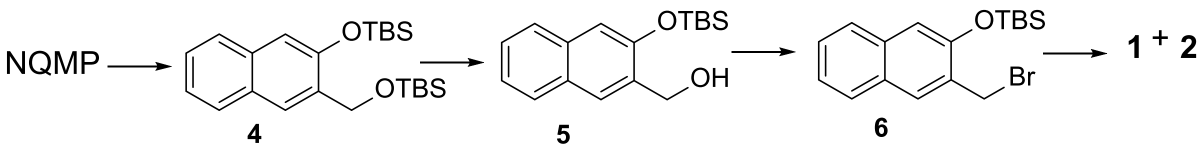

tert-butyl((3-(((tert-butyldimethylsilyl)oxy)methyl)naphthalen-2-yl)oxy)dimethylsilane (**4**).[6] TBDMS chloride (2.9 g, 19 mmol), imidazole (1.3 g, 19 mmol) and DMAP (10 mg) were added to a stirred solution of NQMP (1 g, 5.74 mmol) in DMF (20 mL). The reaction mixture was stirred at room temperature overnight, extracted with ethyl acetate, washed with water (50 mL×3), dried, solvent removed in vacuum, and the residue purified by a flash chromatography (ethyl acetate: hexanes 1:4) to obtain product **4** (2.3 g, 100%) as a yellow oil.

^1H NMR δ 7.89 (s, 1H), 7.78 (d, *J* = 8.0 Hz, 1H), 7.66 (d, *J* = 8.0 Hz, 1H), 7.42 – 7.27 (m, 2H), 7.08 (s, 1H), 4.90 (s, 2H), 1.04 (s, 9H), 1.00 (s, 9H), 0.29 (s, 6H), 0.15 (s, 6H).

$^{13}C\{^1H\}$ NMR δ 151.0, 133.8, 133.4, 129.3, 127.7, 126.3, 125.6, 125.5, 123.7, 112.7, 61.3, 26.1, 25.8, 18.7, 18.4, -4.1, -5.2.

(3-((tert-butyldimethylsilyl)oxy)naphthalen-2-yl)methanol (**5**).[7] 0.4 mL of 48% aqueous hydrofluoric acid (20 mmol) was added to a stirred solution of compound **4** (2 g, 5 mmol) in acetonitrile (30 mL) at 50 °C (using oil bath) and stirred for 15 min. The reaction was quenched by solid $NaHCO_3$, filtered, concentrated in vacuo, and dissolved in ethyl acetate. The organic layer was washed with water and brine, evaporated, purified by chromatography (ethyl acetate: hexanes 1:4) to afford the product **5** (1.14 g, 80%) as a yellow oil.

^1H NMR δ 7.76 (s, 1H) 7.75 (d, 1H), 7.67 (dd, 1H), 7.41 (ddd, 1H), 7.34 (ddd, 1H), 7.14 (s, 1H), 4.82 (d, 2H), 2.30 (t, 1H), 1.05 (s, 9H), 0.33 (s, 6H).

$^{13}C\{^1H\}$ NMR δ 151.8, 133.9, 132.8, 129.1, 127.6, 127.4, 126.3, 126.1, 124.0, 113.3, 62.4, 25.8, 18.2, -4.2.

((3-(bromomethyl)naphthalen-2-yl)oxy)(tert-butyl)dimethylsilane (**6**).[7] PBr_3 (0.47 g, 1.73 mmol) was added dropwise to a solution of compound **5** (1 g, 3.47 mmol) in anhydrous ether (6 mL). The reaction mixture was stirred at room temperature for 0.5 h, quenched by saturated sodium bicarbonate solution (50 mL), and extracted by ethyl ether. The organic layer was dried

and concentrated in vacuo. The residue was purified by chromatography (ethyl acetate: hexanes 1:30) to afford **6** (1 g, 83%) as a white solid.

^{1}H NMR δ 7.95 (s, 1H), 7.87 (dd, *J* = 8.2, 4.5 Hz, 1H), 7.86 (dd, *J* = 8.2, 4.5 Hz, 1H), 7.60 (dt, *J* = 8.2, 6.8 Hz, 1H), 7.51 (dt, *J* = 8.2, 6.8 Hz, 1H), 7.38 (s, 1H), 4.86 (s, 2H), 1.34 (s, 9H), 0.59 (s, 6H).

^{13}C{^{1}H} NMR δ 152.0, 135.0, 130.9, 130.2, 129.1, 127.9, 127.0, 126.6, 124.4, 113.6, 29.9, 26.2, 18.6, -3.8.

3,3'-(disulfanediylbis(methylene))bis(naphthalen-2-ol) DNQMP-S (**2**).[8] A solution of **6** (5.1 g, 14.5 mmol) in EtOH (40 mL) was added to a solution of thiourea (1.2 g, 15.8 mmol) in warm EtOH (40 mL). Then the mixture was heated to reflux using heating mantle and kept for 2.5 h. NaOH solution (1.5 g, 37.5 mmol) in H_2O (20 mL) was slowly added and white solid precipitates formed. Another 50 mL EtOH was added to the mixture and a light-yellow homogeneous solution was formed. The mixture was heated to 80 °C (using oil bath) for 30 min. The reaction mixture was allowed to reach r.t. and quenched with 2 M HCl (20.5 mL). The reaction mixture was extracted with ethyl acetate, washed with brine, dried, and concentrated in vacuo. The crude product was purified by column chromatography (ethyl acetate: hexanes 1:3) to obtain DNQMP-S (**2**) (1.5 g, 54%). The major byproduct was NQMP-SH (**1**, 27%). If the reaction mixture is separated without any delays, the ratio changes to 20% of **2** and 61% of **1**. The treatment of the mixture of **1** and **2** with LAH quantitatively produces NQMP-SH (**1**).

^{1}H NMR (CD_3OD) δ 7.62 (d, *J* = 8.2 Hz, 2H), 7.57 (d, *J* = 8.2 Hz, 2H), 7.40 (s, 2H), 7.27 (m, 4H), 7.09 (s, 2H), 3.77 (s, 4H).

^{13}C{^{1}H} NMR (CD_3OD) δ 153.5, 134.6, 130.0, 128.2, 127.3, 126.3, 125.6, 125.4, 122.7, 108.4, 38.1

IR (neat) cm^{-1}: 3483 vs, br (O-H); 3034 w (C_{Ar}-H); 1626 s, 1507 s (Ar).

HRMS (ESI/ Orbitrap), *m/z*: $[M-H]^-$ Calcd for $C_{22}H_{17}O_2S_2$ 377.0675; Found 377.0671.

Alternative preparative rout.

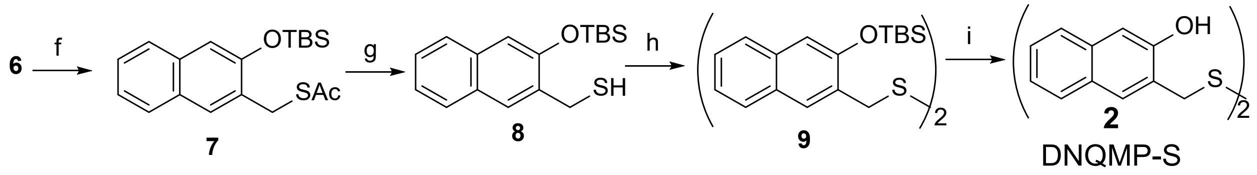

(3-((tert-butyldimethylsilyl)oxy)naphth-2-yl)methyl thioacetate (**7**). Compound **6** (1 g, 2.8 mmol) was added to a solution of potassium thioacetate (0.65 g, 5.6 mmol) in DMF (10 mL) at 0 °C (using ice-water bath). The mixture was stirred at 0 °C for 10 min, then diluted with diethyl ether. The organic layer was washed with water, brine, dried over Na_2SO_4, solvents evaporated, and the residue purified by flash silica chromatography (ethyl acetate: hexanes 1:30) to afford **7** (0.75 g, 76%) as a colorless oil.

^{1}H NMR δ 7.63 (s, 1H), 7.53 (d, *J* = 8.1 Hz, 1H), 7.44 ((d, *J* = 8.1 Hz, 1H), 7.19 (dt, *J* = 8.1, 6.8 Hz, 1H), 7.11 (dt, *J* = 8.1, 6.8 Hz, 1H), 6.93 (s, 1H), 4.07 (s, 2H), 2.12 (s, 3H), 0.87 (s, 9H), 0.15 (s, 6H).

^{13}C{^{1}H} NMR δ 195.6, 152.0, 134.0, 129.8, 129.7, 128.9, 127.6, 126.3, 126.2, 120.0, 113.0, 30.5, 29.3, 25.9, 18.4, -4.1.

(3-((tert-butyldimethylsilyl)oxy)naphthalen-2-yl)methanethiol (**8**). $NaOCH_3$ (0.16 g, 3 mmol) was added to a stirred solution of thioacetate **7** (0.35 g, 1 mmol) in THF (4 mL)/CH3OH (4 mL) at 0 °C (using ice-water bath). The mixture was stirred at 0 °C for 5 min, quenched by saturated aqueous NH_4Cl, extracted by ethyl acetate (30 mL x 3). The organic layer was washed with brine, dried over Na_2SO_4, evaporated, and purified by chromatography (ethyl acetate: hexanes 1:30) to afford **8** (0.2 g, 65%) as a colorless to light yellow liquid.

^{1}H NMR δ 7.53 (s, 1H), 7.52 (d, *J* = 8.2 Hz, 1H), 7.46 (d, *J* = 8.2 Hz, 1H), 7.19 (dt, *J* = 8.2, 6.8 Hz, 1H), 7.14 (dt, *J* = 8.2, 6.8 Hz, 1H), 6.94 (s, 1H), 3.66 (d, *J* = 8.0 Hz, 2H), 1.72 (t, *J* = 8.0 Hz, 1H), 0.88 (s, 9H), 0.15 (s, 6H).

^{13}C NMR δ 151.6, 133.9, 133.5, 129.1, 128.4, 127.4, 126.3, 126.0, 124.0, 113.4, 25.9, 24.8, 18.4, -4.1.

MS (ESI/quadrupole filter), *m/z*: $[M+H]^+$ Calcd for $C_{17}H_{25}OSSi$ 305.14; Found 305.12.

1,2-bis((3-((tert-butyldimethylsilyl)oxy)naphthalen-2-yl)methyl)disulfane (**9**). 30% hydrogen peroxide solution (0.34 mL, 3 mmol) was added to a solution of compound **8** (0.2 g, 0.66 mmol) in ethyl acetate (3 mL). Then, sodium iodide (0.02 g, 0.13 mmol) was added to the stirred solution as bubbling was observed. The mixture was stirred at room temperature for 3 days and extracted by ethyl acetate. The organic layer was washed by water and brine, dried, and solvent evaporated. The crude product was purified by column chromatography (ethyl acetate : hexanes 1:30) to provide disulfide **9** (0.2 g, quant.) as a yellow solid. It has been used in the next step without further purification.

^{1}H NMR δ 7.86 – 7.78 (m, 4H), 7.63 (m, 2H), 7.53-7.49 (m, 4H), 7.30 – 7.28 (m, 2H), 3.92 (m, 4H), 1.24 – 1.21 (m, 18H), 0.49 (m, 12H).

$^{13}C\{^1H\}$ NMR δ 152.1, 134.3, 130.4, 130.3, 129.6, 127.7, 126.5, 126.3, 124.1, 113.2, 39.7, 26.1, 18.5, -3.9.

3,3'-(disulfanediylbis(methylene))bis-(naphthalen-2-ol), **DNQMP-S** (**2**). 0.16 mL of TBAF solution (1M in THF, 0.16 mmol) was added to a solution of compound **9** (0.1 g, 0.16 mmol) in THF (4 mL) at 0 °C (using ice-water bath), poured into brine (30 mL), extracted with ethyl acetate. The organic layer was dried and solvent removed in vacuum. The crude product was purified by column chromatography (ethyl acetate: hexanes 1:3) to obtain 10 mg of DNQMP-S (**2**) (17%) as a light-yellow solid.

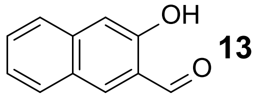

13

3-hydroxy-2-naphthaldehyde (**13**).[9] A solution of DNQMP-S (**2**) (28 mg, 0.5 mmol) in 290 mL of aqueous acetonitrile (1:1) was irradiated with 300 nm light for 5 min. The reaction mixture was extracted with ethyl acetate. The organic layer was washed with brine, dried, solvent removed in vacuum, and the residue purified by chromatography to provide 12 mg (43%) of aldehyde **13** as a yellow solid.

^1H NMR δ 10.26 (s, 1H), 10.02 (d, J = 0.6 Hz, 1H), 8.09 (s, 1H), 7.81 (d, J = 8.4 Hz, 1H), 7.65 (d, J = 8.4 Hz, 1H), 7.50 (t, J = 8.4 Hz, 1H), 7.31 (t, J = 8.4 Hz, 1H), 7.22 (s, 1H).

$^{13}C\{^1H\}$ NMR δ 196.8, 155.9, 138.2, 137.9, 130.3, 129.4, 127.4, 126.7, 124.5, 122.3, 110.0.

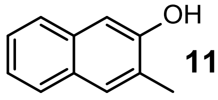

11

3-Methyl-2-naphthol (**11**).[10] A solution of NQMP (25 mg, 0.14 mmol) and ethyl vinyl ether (1.0 g, 14 mmol) in acetonitrile/water (1:1, 130 mL) was irradiated at 254 nm for 1.5 h. Photolysate was extracted with ethyl acetate, dried, concentrated in vacuo. The residue was purified by chromatography (ethyl acetate: hexanes 1:30) to afford **11** (10 mg, 44%) as white powder.

^1H NMR δ 7.70 (d, J = 8.2 Hz, 1H), 7.64 (d, J = 8.2 Hz, 1H), 7.59 (s, 1H), 7.36 (d, J = 8.2 Hz, 1H), 7.29 (t, J = 8.2 Hz, 1H), 7.10 (s, 1H), 5.06 (s, 1H), 2.42 (s, 3H).

$^{13}C\{^1H\}$ NMR δ 152.7, 133.4, 129.4, 129.2, 120.0, 126.4, 125.8, 125.6, 123.5, 109.0, 16.5.

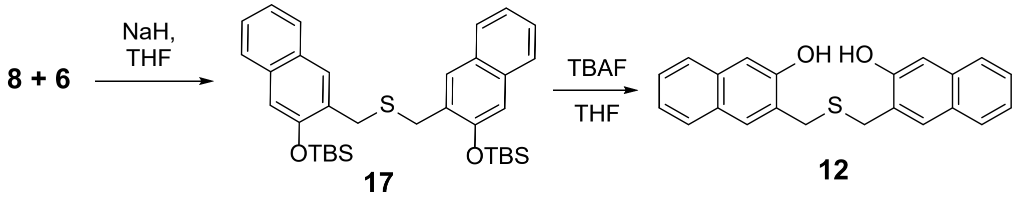

bis((3-((tert-butyldimethylsilyl)oxy)naphthalen-2-yl)methyl)sulfane (**17**). Sodium hydride (0.1 g of 60% dispersion in oil, 3 mmol) was added to the stirred solution of thiol **8** (0.3 g, 1 mmol) in THF (6 mL) at 0 °C (using ice-water bath). Then, bromide **6** (0.35 g, 1 mmol) was added to the reaction mixture, and stirred at 0 °C for 30 min. The reaction mixture was quenched by saturated aqueous NH_4Cl, extracted by ethyl acetate, the organic layer was washed with brine, dried, solvent evaporated, and the residue purified by chromatography (ethyl acetate: hexanes 1:30) to afford sulfide **17** (0.3 g, 64%) as a colorless oil.

^{1}H NMR δ 7.64 (s, 2H), 7.61 – 7.57 (m, 4H), 7.31 (t, *J* = 8.2 Hz, 2H), 7.24 (t, *J* = 8.2 Hz, 2H), 7.08 (s, 2H), 3.79 (s, 4H), 0.93 (s, 18H), 0.22 (s, 12H).

^{13}C{^{1}H} NMR δ 152.3, 134.0, 130.5, 129.2, 129.0, 127.5, 126.3, 125.9, 123.9, 113.5, 31.7, 26.0, 18.4, -4.0.

MS (ESI/quadrupole filter) *m/z*: $[M+H]^+$ Calcd for $C_{34}H_{47}O_2SSi_2$ $[M+H]^+$ 575.3; Found 575.2.

3,3'-(thiobis(methylene))bis(naphthalen-2-ol) (**12**). TBAF (0.52 mL of 1M in THF solution, 0.52 mmol) was added to a stirred solution of compound **17** (0.3 g, 0.52 mmol) in THF (4 mL) at 0 °C, stirred for 5 min, and diluted with ethyl acetate. The organic layer was washed with brine, dried, solvent evaporated in vacuum. The crude product was purified by column chromatography (ethyl acetate: hexanes 1:3) to afford target sulfide **12** (0.05 g, 27%) as a light-yellow solid.

NMR spectra shows the presence of two conformation in the ratio of ~ 6:10.

^{1}H NMR δ 7.72 – 7.62 (m 4H), 7.54 (s, 0.8H), 7.47 (s, 1.2H), 7.44 – 7.34 (m, 4H), 7.23 (s, 0.8H), 7.17 (s, 1.2H), 6.76 (s, 0.8H), 6.29 (s, 1.2H), 3.94 (s, 1.6H), 3.89 (s, 2.4H).

^{13}C{^{1}H} NMR δ 152.8, 152.1, 134.4, 130.7, 130.1, 128.8, 128.7, 127.6, 127.4, 126.5, 126.5, 126.3, 126.2, 125.6, 124.9, 124.0, 124.0, 111.5, 110.9, 39.1, 32.3.

HRMS (ESI/ Orbitrap), *m/z*: $[M-H]^-$ Calcd for $C_{22}H_{17}O_2S$ 345.0955; Found 345.0953.

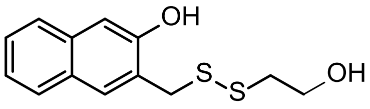

15c

3-(((2-hydroxyethyl)disulfaneyl)methyl)naphthalen-2-ol (**15c**). 2-Mercaptoethanol (36 mg, 150 mmol) was added to 30 mL of 1 mM solution of DNQMP-S (**2**) in acetonitrile/water (1:1) and left in dark for 10 h. The reaction mixture was extracted with ethyl acetate, dried, and concentrated in vacuo. The residue was separated by chromatography (ethyl acetate: hexanes 1:3) to produce 13 mg (77%) of **15c** as colorless oil.

^1H NMR δ 7.75 – 7.70 (m, 2H), 7.60 (d, *J* = 8.2 Hz, 1H), 7.40 (t, *J* = 8.2 Hz, 1H), 7.33 (t, *J* = 8.2 Hz, 1H), 7.13 (s, 1H), 6.95 (s, 1H), 4.12 (s, 2H), 3.84 (t, *J* = 5.7 Hz, 2H), 2.60 (t, *J* = 5.7 Hz, 2H), 2.01 (s, 1H).

$^{13}C\{^1H\}$ NMR δ 152.3, 134.3, 130.5, 128.7, 127.5, 126.5, 126.1, 125.6, 123.9, 110.4, 60.8, 40.6, 38.9.

MS (ESI/quadrupole filter), *m/z*: $[M-H]^-$ Calcd for $C_{13}H_{13}O_2S_2$ 265.0; Found 264.8.

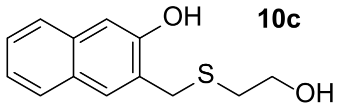

10c

3-(((2-hydroxyethyl)thio)methyl)naphthalen-2-ol (10c). A solution of NQMP-SH (**1**) (27.5 mg, 0.144 mmol) and 2-mercaptoethanol (1.13 g, 14.5 mmol) in aqueous acetonitrile (1:1, 145 mL) was irradiated with 15 fluorescent UV lamps (254 nm) in quartz vessel for 20 min with stirring. Photolysate was extracted with ethyl acetate, dried, concentrated in vacuo. The product was separated by column chromatography (ethyl acetate: hexanes 1:5) to give 16 mg (42%) of **10c** as a colorless oil (contained small impurity of mercaptoethanol).

^1H NMR δ 7.71 (d, *J* = 8.1 Hz, 1H), 7.66 (d, *J* = 8.2 Hz, 1H), 7.62 (s, 1H), 7.40 (dt, *J* = 8.2, 1.3 Hz, 1H), 7.32 (dt, *J* = 8.1, 1.2 Hz, 1H), 7.23 (s, 1H), 3.99 (s, 2H), 3.79 (t, *J* = 5.9 Hz, 2H), 2.88 (t, *J* = 5.9 Hz, 2H).

$^{13}C\{^1H\}$ NMR δ 152.8, 134.3, 129.7, 128.7, 127.4, 126.4, 126.2, 125.6, 123.9, 111.4, 61.1, 33.8, 32.2.

MS (ESI/quadrupole filter): *m/z*: $[M-H]^-$ Calcd for $C_{13}H_{13}O_2S$ 233.1; Found 232.8.

HRMS (ESI/ Orbitrap): *m/z*: $[M+H]^+$ Calcd for $C_{13}H_{15}O_2S$ 235.0787; Found 235.0789.

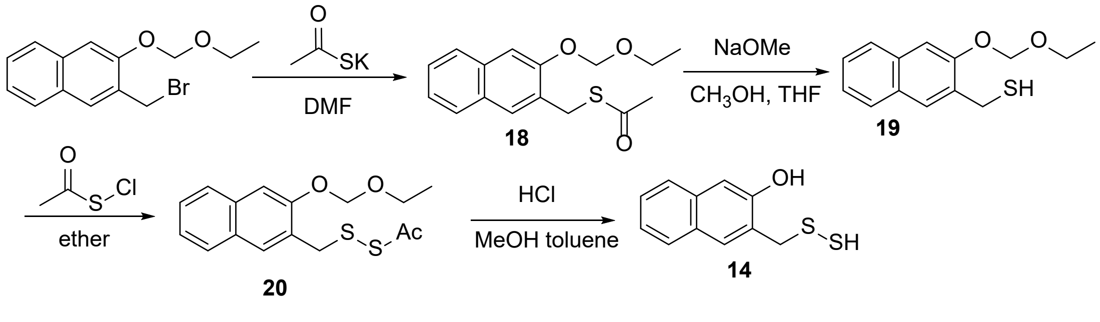

***S*-((3-(ethoxymethoxy)naphthalen-2-yl)methyl) ethanethioate** (**18**). 2-(Bromomethyl)-3-(ethoxymethoxy)naphthalene[5] (9.9 g, 3 mmol) in DMF (10 mL) was added to a stirred solution of potassium thioacetate (6 g, 53 mmol) in DMF (25 mL) at 0 °C (using ice-water bath), was stirred for 20 min at 0 °C, and diluted with ethyl acetate. The organic layer was washed with water (30

mL×10), brine, dried, filtered, concentrated in vacuo, and purified by chromatography (ethyl acetate: hexanes 1:9) to afford **18** (8.8 g, 91%) as a light-yellow liquid.

^{1}H NMR δ 7.79 (s, 1H), 7.70-7.66 (m, 2H), 7.40 – 7.33 (m, 2H), 7.32 – 7.26 (m, 1H), 5.33 (s, 2H), 4.27 (s, 2H), 3.74 (q, *J* = 7.1 Hz, 2H), 2.27 (s, 3H), 1.23 (t, *J* = 7.1 Hz, 3H).

^{13}C{^{1}H} NMR δ 195.4, 153.2, 134.1, 129.6, 129.0, 127.8, 127.6, 126.8, 126.4, 124.3, 108.8, 93.0, 64.7, 30.5, 29.2, 15.3.

(3-(ethoxymethoxy)naphthalen-2-yl)methanethiol (**19**). Sodium methoxide (5.2 g, 96 mmol) was added to a stirred solution of compound **18** (8.8 g, 32 mmol) in THF (20 mL) and methanol (20 mL) at 0 °C (using ice-water bath). The reaction mixture was stirred for 20 min at 0 °C, and diluted with ethyl acetate. The organic layer was washed with water, brine, dried, filtered, evaporated, and purified by chromatography (ethyl acetate: hexanes 1:19) to afford **19** (6.1 g, 82%) as a colorless oil.

^{1}H NMR δ 7.72 – 7.68 (m, 2H), 7.66 (s, 1H), 7.40 – 7.35 (m, 2H), 7.35 – 7.27 (m, 1H), 5.39 (s, 2H), 3.86 (d, *J* = 8.0 Hz, 2H), 3.78 (q, *J* = 7.1 Hz, 2H), 1.96 (t, *J* = 8.0 Hz, 1H), 1.24 (t, *J* = 7.1 Hz, 3H).

^{13}C{^{1}H} NMR δ 153.0, 133.9, 131.6, 129.2, 128.2, 127.4, 126.9, 126.2, 124.3 109.1, 93.2, 64.7, 24.7, 15.3.

Acetyl (3-(ethoxymethoxy)naphth-2-yl)methyl) disulfide (**20**). Acetylsulfenyl chloride[11] (2 g, 18 mmol) in ether (5 mL) was added dropwise to a stirred solution of thiol **19** (0.7 g, 3 mmol) in ether (10 mL) in at 0 °C (using ice-water bath). The reaction mixture was stirred at room temperature for 15 h, quenched by adding sodium bicarbonate solution. The reaction mixture was diluted with ethyl acetate, the organic layer was separated, washed with water and brine, dried, filtered, and solvent evaporated in vacuum. The residue was purified by chromatography (ethyl acetate: hexanes 1:19) to afford **20** (0.46 g, 50%) as yellow oil.

^{1}H NMR δ 7.75 – 7.73 (m, 2H), 7.60 (s, 1H), 7.45 – 7.41 (m, 2H), 7.39 – 7.34 (m, 1H), 5.45 – 5.36 (m, 2H), 4.31 (s, 2H), 3.86 – 3.74 (m, 2H), 2.50 (s, 3H), 1.31 – 1.20 (m, 3H).

^{13}C{^{1}H} NMR δ 195.5, 153.1, 134.3, 131.2, 130.0, 128.6, 127.5, 126.9, 126.5, 124.3, 109.1, 93.2, 64.7, 38.9, 28.5, 15.2.

MS (ESI/quadrupole filter), *m/z*: : $[M+Na]^+$ Calcd for $C_{16}H_{18}NaO_3S_2$ 345.1; Found 345.0.

(3-hydroxy-naphthalen-2-yl)methylsulfenothioic acid (**14**). A pre-chilled to 0 °C 3M HCl solution in MeOH (1.2 mL) was added dropwise to a stirred solution of **20** (0.23 g, 0.71 mmol) in

EtOH : toluene mixture (1.5 mL EtOH and 1.1ml toluene) at 0 °C (using ice-water bath). The reaction mixture was allowed to reach room temperature and stirred for 24 h, and diluted with ethyl acetate. The organic layer was washed by brine, dried, filtered, solvents evaporated in vacuum, and purified by chromatography (ethyl acetate: hexanes 1:19) to afford the target hydrosulfide **14** (0.08 g, 51%) as a yellow oil.

^{1}H NMR δ 7.77 – 7.68 (m, 2H), 7.66 – 7.58 (m, 1H), 7.44 – 7.37 (m, 1H), 7.36 – 7.29 (m, 1H), 7.17 – 7.07 (m, 1H), 5.68 – 5.63 (m, 1H), 4.36-4.31(m, 2H), 1.77 (s, 1H).

^{13}C{^{1}H} NMR δ 151.9, 134.4, 130.9, 128.9, 127.7, 126.6, 126.1, 124.7, 124.0, 110.7, 39.7, 39.2.

MS (ESI/quadrupole filter), *m/z*: [M-H]$^{-}$ Calcd ffor $C_{11}H_{9}OS_{2}$ 221.0; Found 221.0.

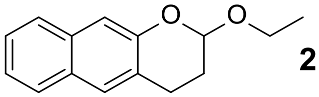

21

2-Ethoxy-3,4-dihydro-*2H*-benzo[*g*]chromene (**21**).[3] A solution of NQMP (27.5 mg, 0.158 mmol) and ethyl vinyl ether (1.1 g, 15.3 mmol) in 145 mL aqueous acetonitrile (1:1) was irradiated with 16 fluorescent UV lamps (254 nm) for 1.5 h with stirring. Photolysate was extracted with ethyl acetate, dried, and solvents removed in vacuum. The residue was separated by chromatography (1:5 EtOAc: hexane) to give **21** (17.3 mg, 48%) as a colorless oil.

^{1}H NMR δ 7.68 (m, 2H), 7.52 (s, 1H), 7.36 – 7.27 (m, 2H), 7.22 (s, 1H), 5.32 (t, *J* = 2.8 Hz, 1H), 3.92 (m, 1H), 3.67 (m, 1H), 3.19 (m, 1H), 2.85 (m, 1H), 2.28 (m, 2H), 1.18 (t, *J* = 7.0 Hz, 3H).

^{13}C{^{1}H} NMR δ 151.0, 133.5, 128.9, 127.6, 127.0, 126.3, 125.4, 124.6, 123.5, 111.7, 97.2, 63.7, 26.8, 21.0, 15.1.

HRMS (ESI/ Orbitrap): *m/z*: [M+H]$^{+}$ Calcd $C_{15}H_{17}O_{2}$ 229.1223; Found 229.1223.

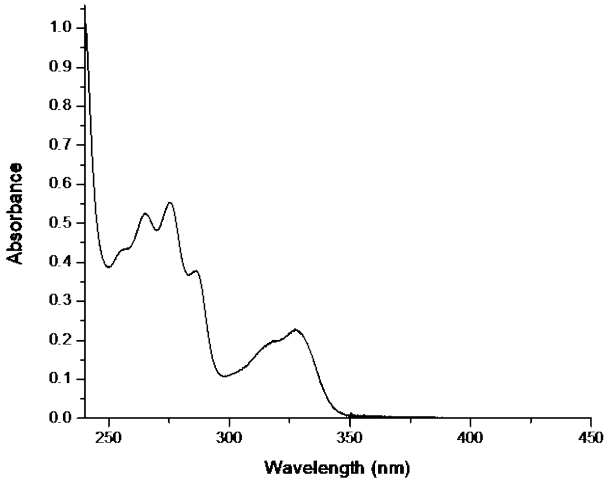

UV spectrum of 0.1 mM of NQMP-SH in MeOH

REFERENCES

[1] The emission spectra of the lamps can be found on the Southern New England Ultraviolet Co. website: https://www.rayonet.org/shop.php?pline=lamps&compat=600.

[2] Devarie-Baez, N.O., Bagdon, P.E., Peng, B., Zhao, Y., Park, C.M. and Xian, M., Light-induced hydrogen sulfide release from "caged" gem-dithiols. *Org. Lett.* **2013,** *15*(11), 2786-2789.

[3] Arumugam, S.; Popik, V.V. "Photochemical Generation and the Reactivity of o-Naphthoquinone Methides in Aqueous Solutions", *J. Am. Chem. Soc.* **2009**, *131*(33), 11892-9.

[4] Chen, C.-T.; Kuo, J.-H.; Pawar, V. D.; Munot, Y. S.; Weng, S.-S.; Ku, C.-H.; Liu, C.-Y. "Nucleophilic acyl substitutions of anhydrides with protic nucleophiles catalyzed by amphoteric, oxomolybdenum species", *J. Org. Chem.*, **2005**, *70*(4) 1188–1197.

[5] Nekongo, E. E.; Popik V.V. "Photoactivatable Fluorescein Derivatives Caged with (3-Hydroxy-2-naphthalenyl)methyl Group", *J. Org. Chem.* **2014**, *79* (16), 7665-7671: DOI: 10.1021/jo501116g.

[6] Bunel, E.E. "Preparation of linear aldehydes", Patent US6175043 B1 2001-01-16.

[7] Kulikov, A.; Arumugam, S.; Popik, V.V. "Photolabile Protection of Alcohols, Phenols, and Carboxylic Acids with 3-Hydroxy-2-Naphthalenemethanol" *J. Org. Chem.* **2008**, *73*(19), 7611-7615; https://pubs.acs.org/doi/10.1021/jo801302m.

[8] Cohen, V. I. "A Convenient Alkyl, Cycloalkyl and Aralkyl Disulfides Synthesis from Aliphatic and Aromatic Aldehydes, Aliphatic Ketones and Cycloketones", Helv. Chim. Acta, **1976**, *59*(3), 840-844; DOI: 10.1002/hlca.19760590313; Buyukliev, R., "Synthesis of diaryl and di(arylmethyl) disulfides with hydrophilic substituents for use as additives to acid copper electrolytes", *Bulgarian Chem. Commun.*, **1990**, *23*(3), 456 – 464.

[9] Palusiak, M.; Simon, S.; Sola, M. "Interplay between Intramolecular Resonance-Assisted Hydrogen Bonding and Aromaticity in o-Hydroxyaryl Aldehydes", *J. Org. Chem.*, **2006**, *71*(14), 5241 – 5248; https://pubs.acs.org/doi/10.1021/jo060591x.

[10] Rong, Ming-Guang; Qin, Tian-Zhu; Liu, Xin-Rui; Wang, Hong-Fa; Zi, Weiwei "De Novo Synthesis of Phenols and Naphthols through Oxidative Cycloaromatization of Dienynes", *Org. Lett.*, **2018**, *20*(19), 6289 -6293; https://pubs.acs.org/doi/10.1021/acs.orglett.8b02786

[11] Bailey, T. Spencer; Zakharov, Lev N.; Pluth, Michael D. "Understanding Hydrogen Sulfide Storage: Probing Conditions for Sulfide Release from Hydrodisulfides", *J. Am. Chem. Soc.*, **2014**, *136*(30), 10573-10576.

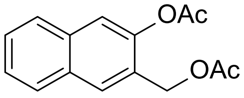

3

^{1}H NMR (400 MHz, $CDCl_3$)

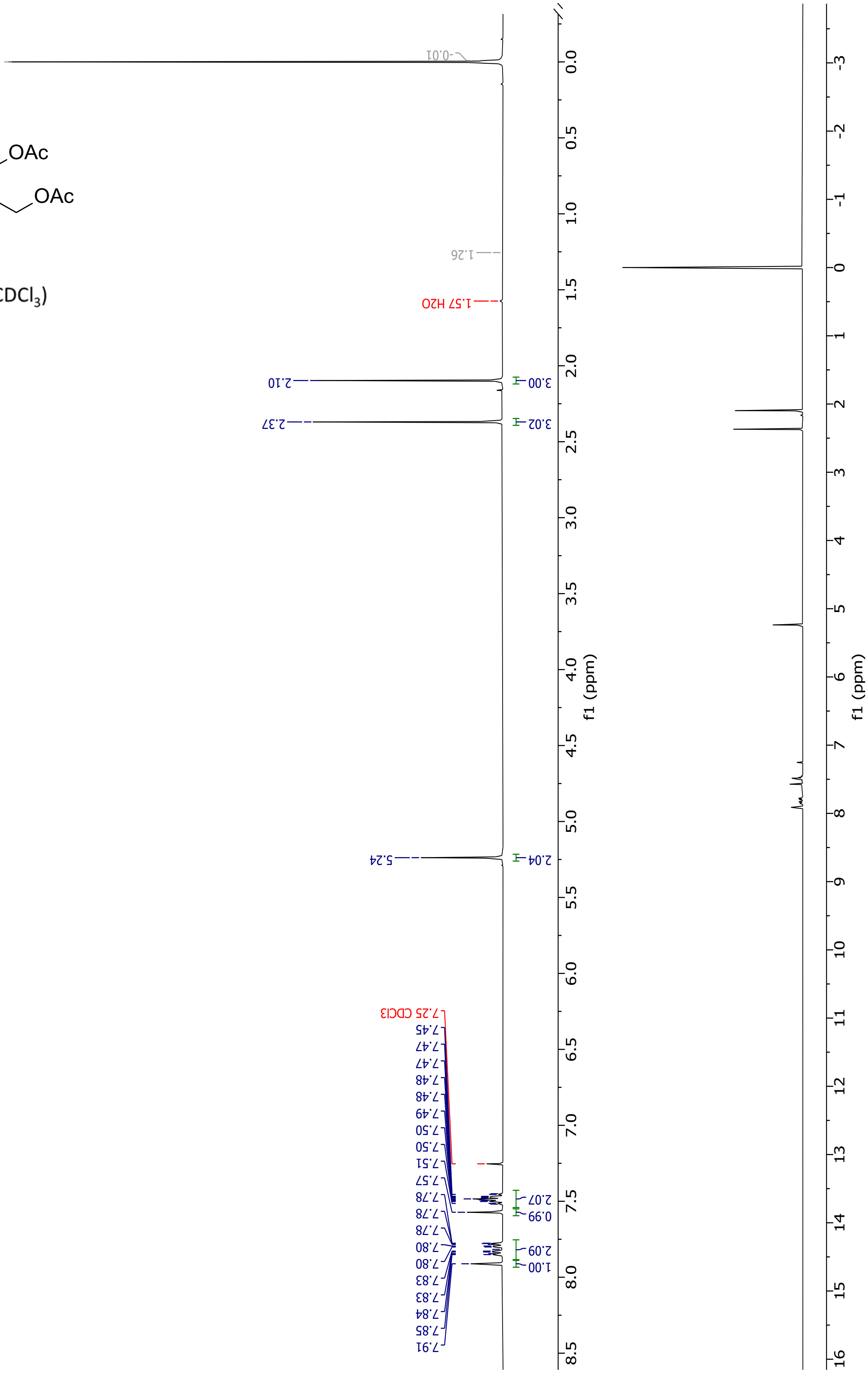

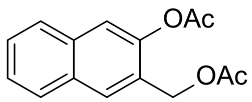

3

$^{13}C\{^{1}H\}$ NMR (101 MHz, $CDCl_3$)

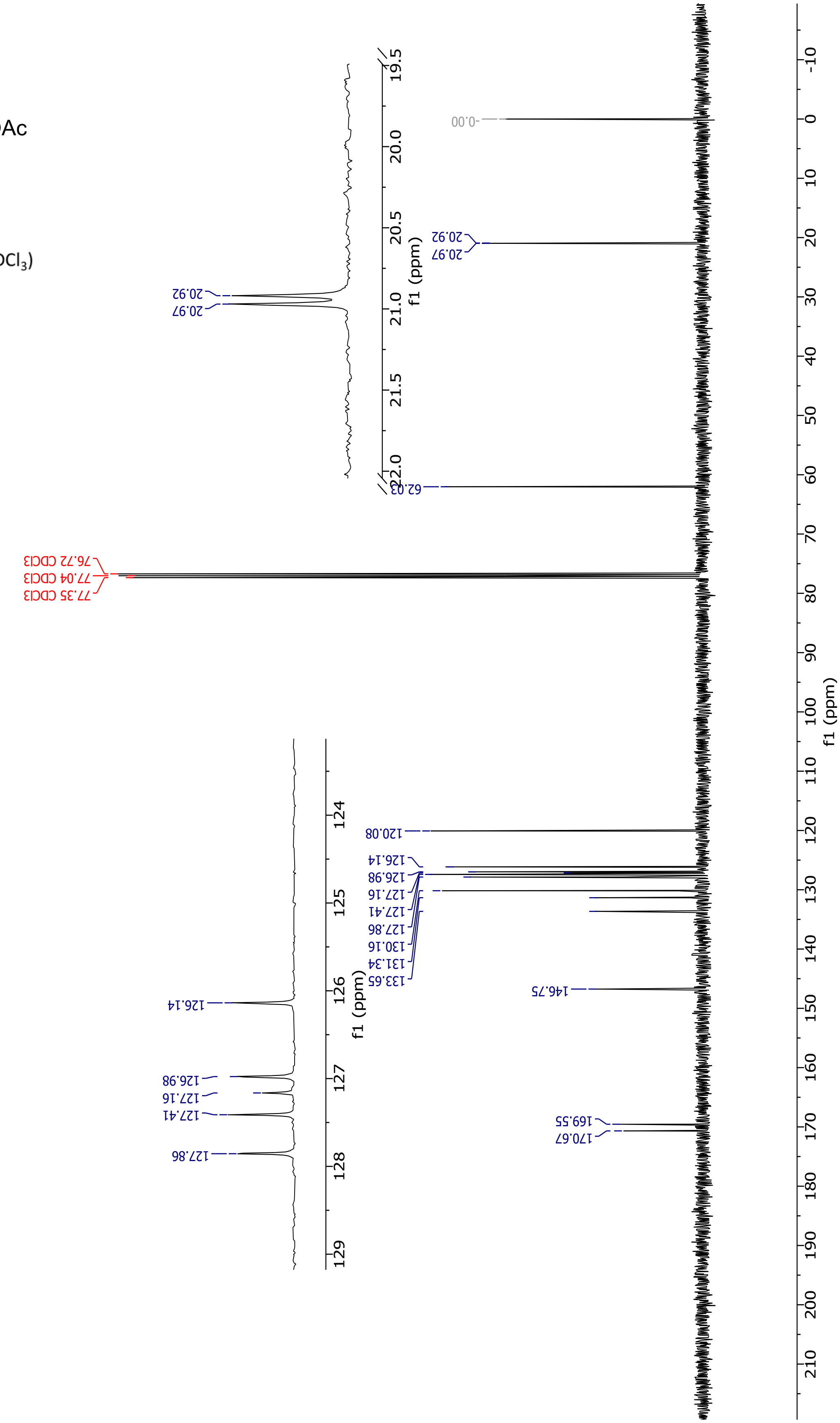

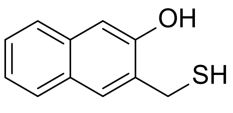

1

^{1}H NMR (400 MHz, $CDCl_3$)

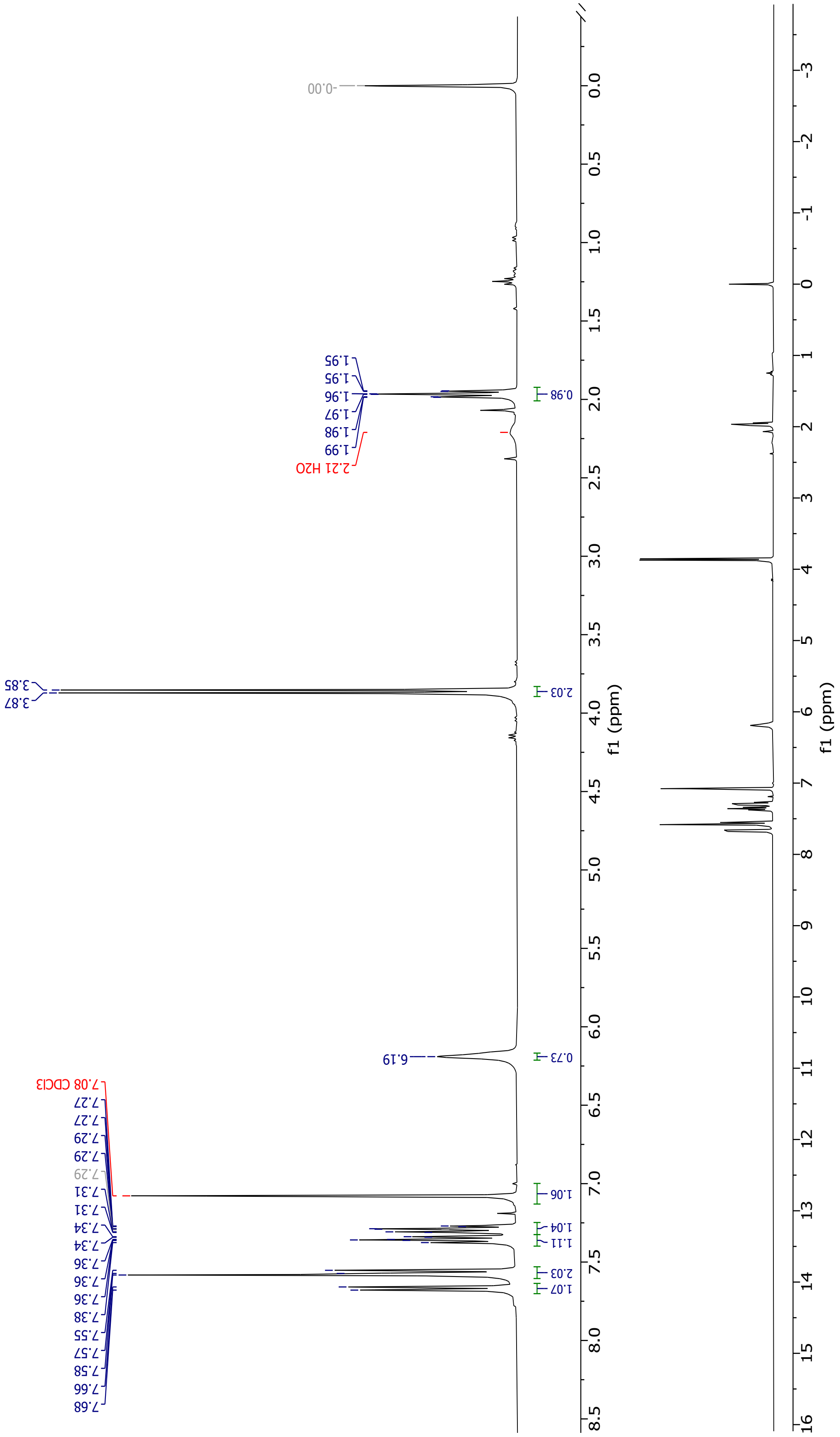

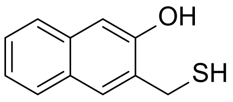

1

$^{13}C\{^{1}H\}$ NMR (101 MHz, $CDCl_3$)

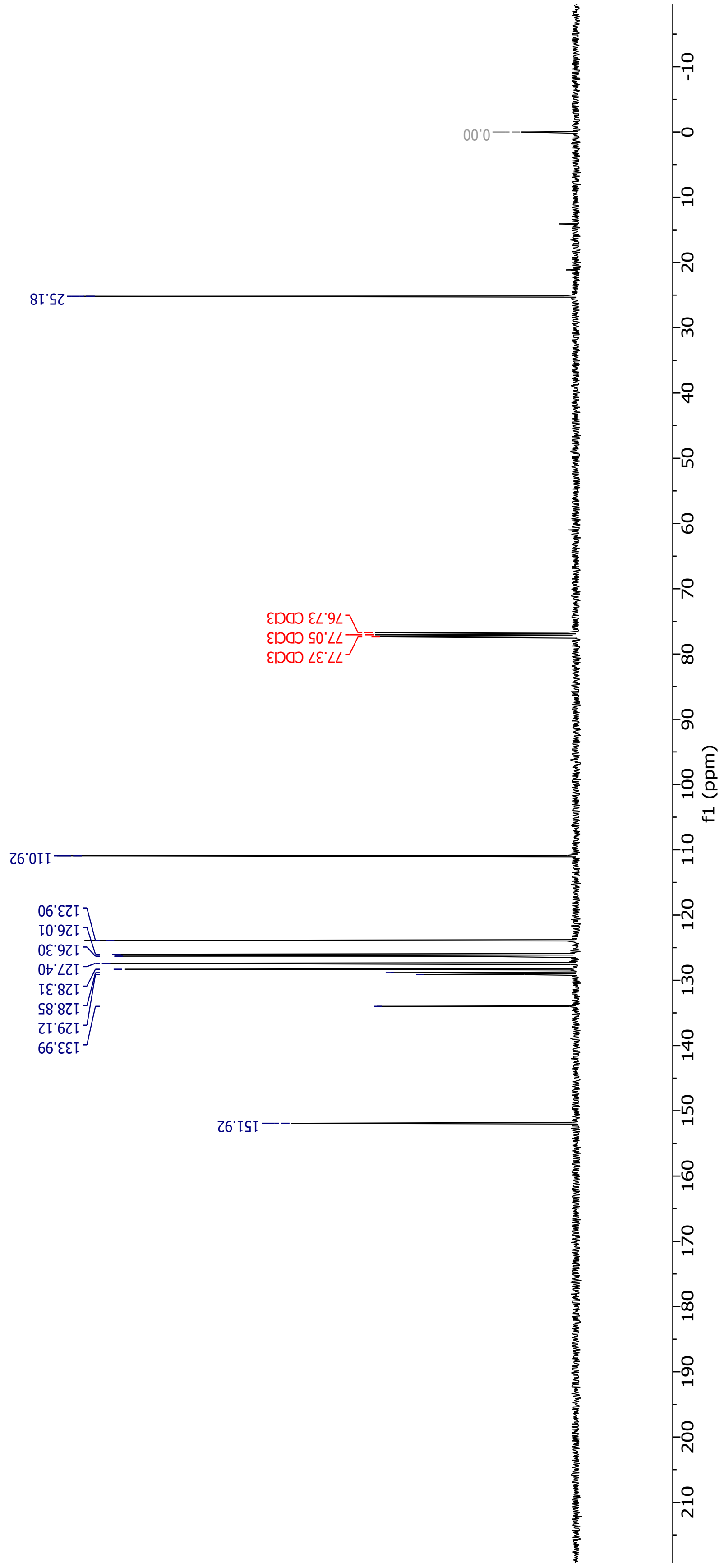

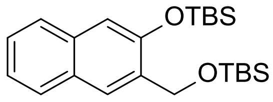

4

^{1}H NMR (400 MHz, $CDCl_3$)

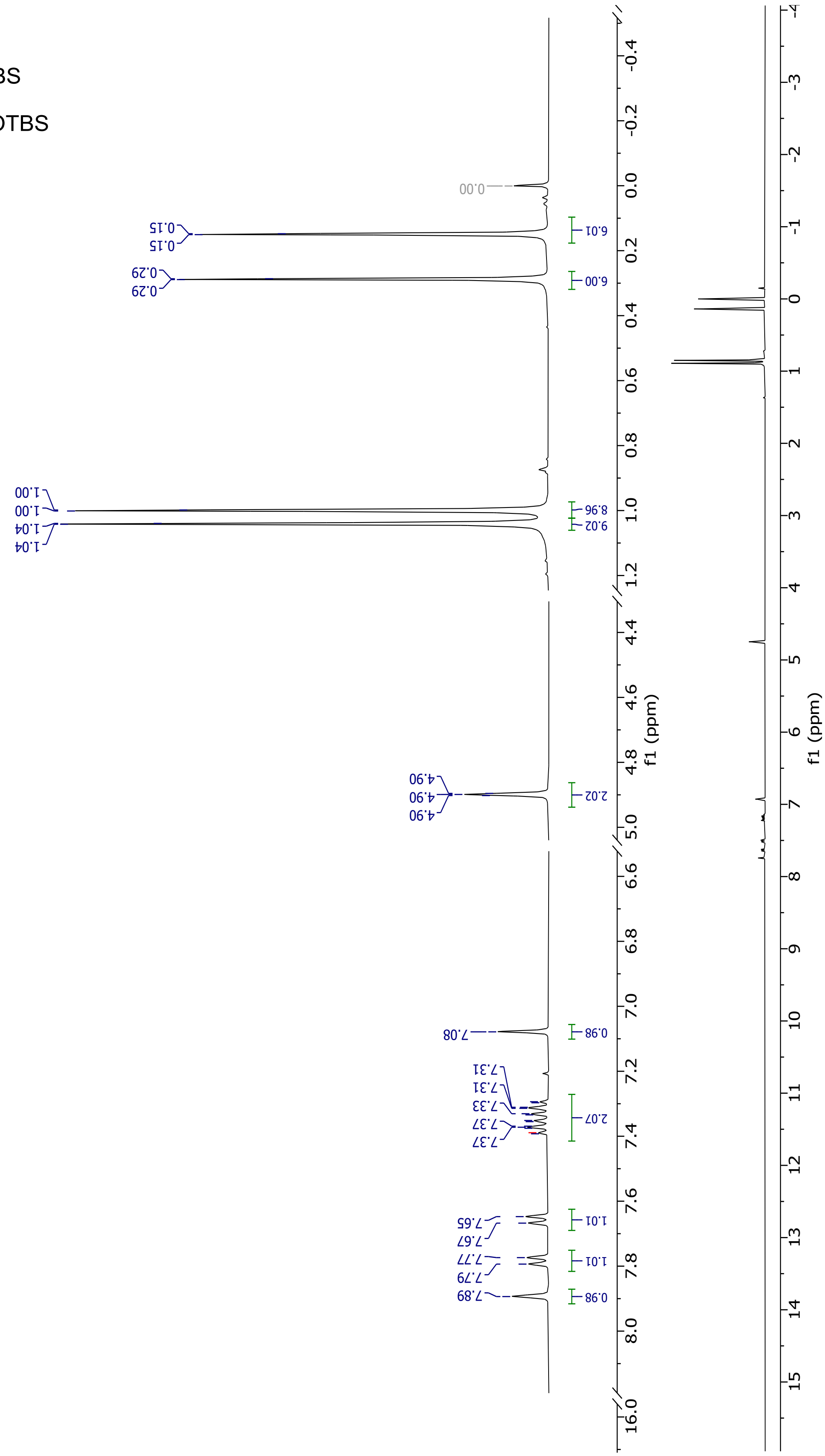

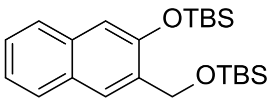

4

$^{13}C\{^{1}H\}$ NMR (101 MHz, $CDCl_3$)

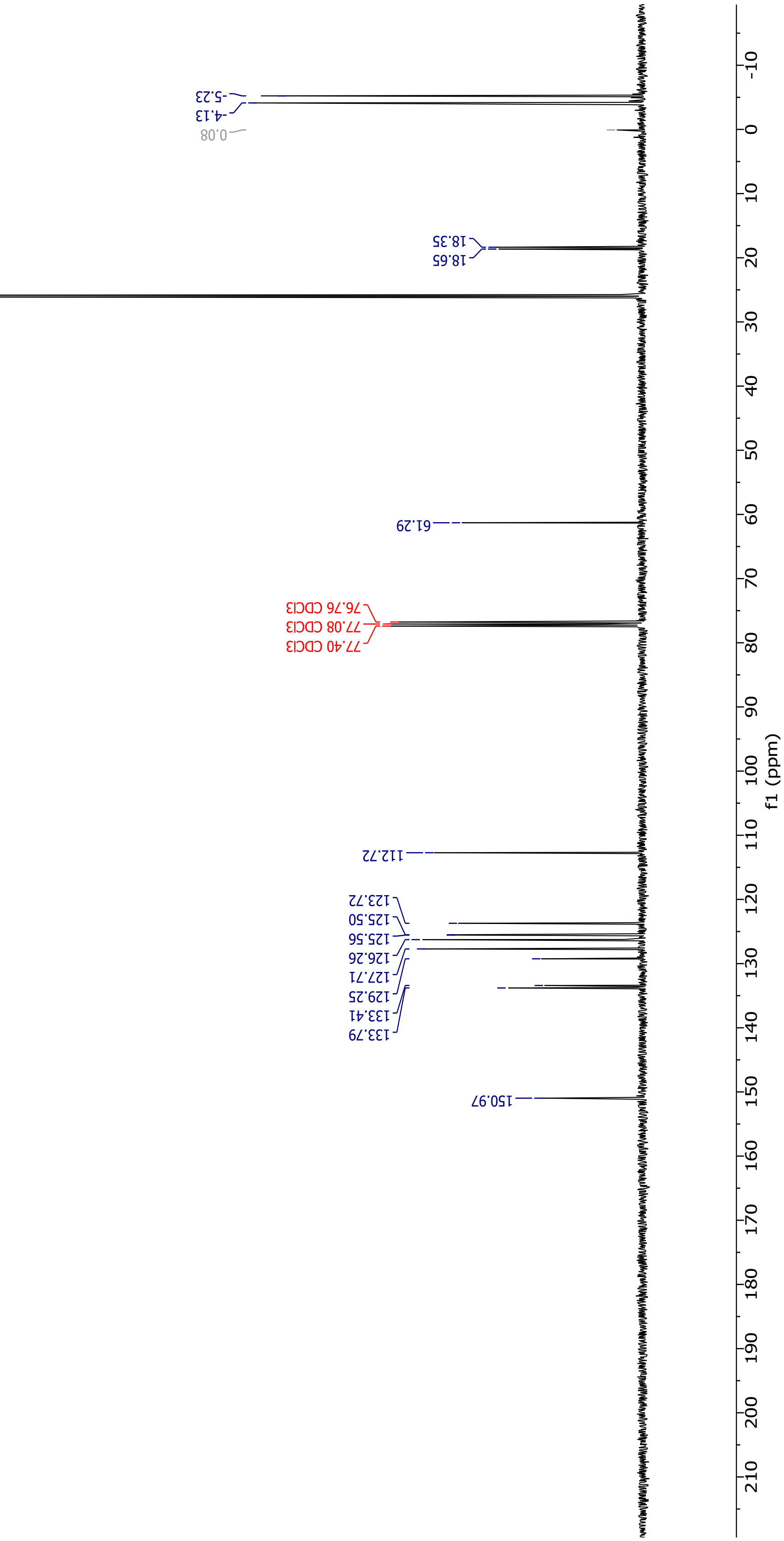

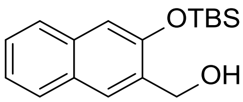

5

^{1}H NMR (400 MHz, $CDCl_3$)

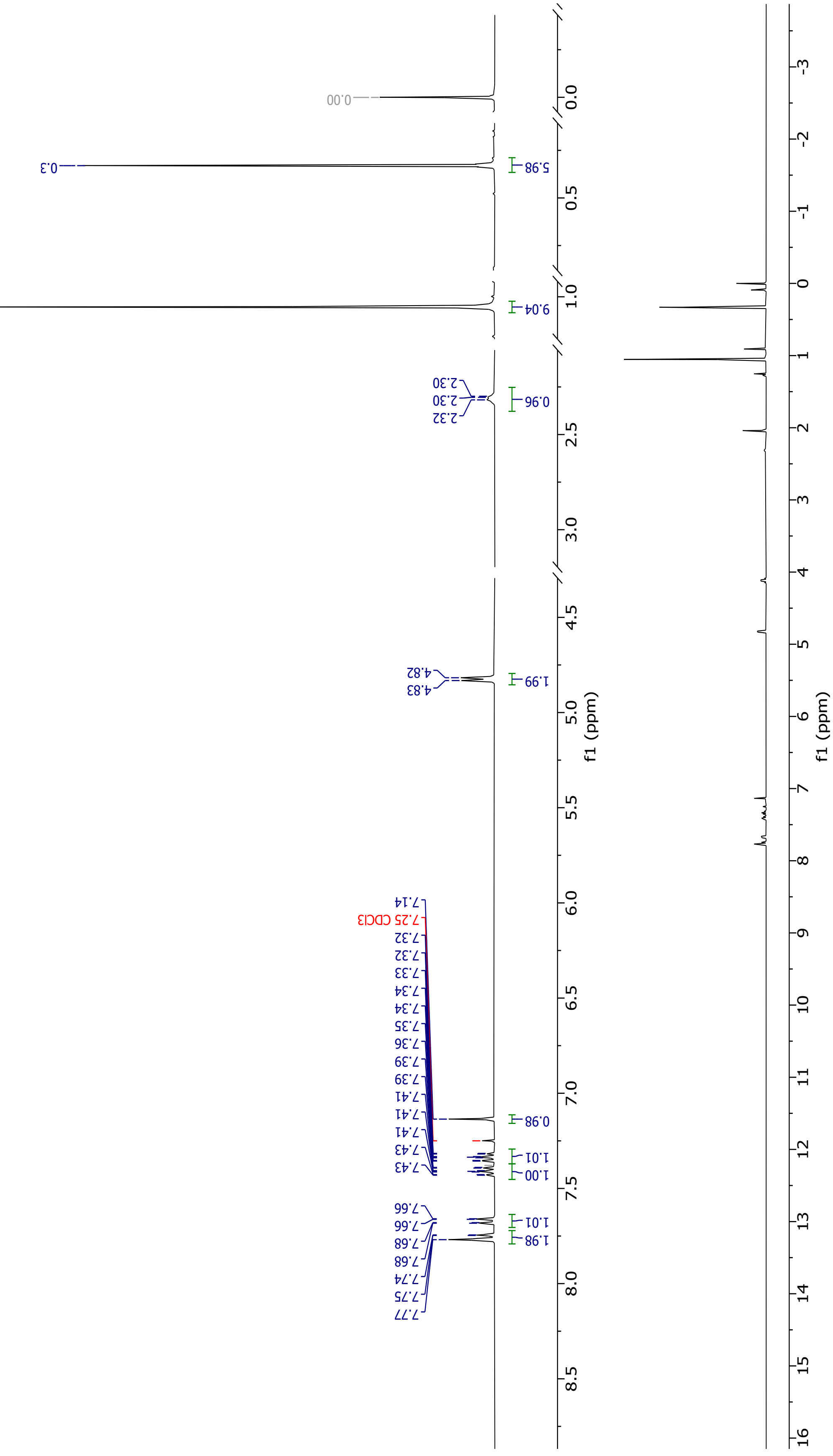

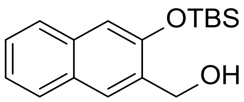

5

$^{13}C\{^{1}H\}$ NMR (101 MHz, $CDCl_3$)

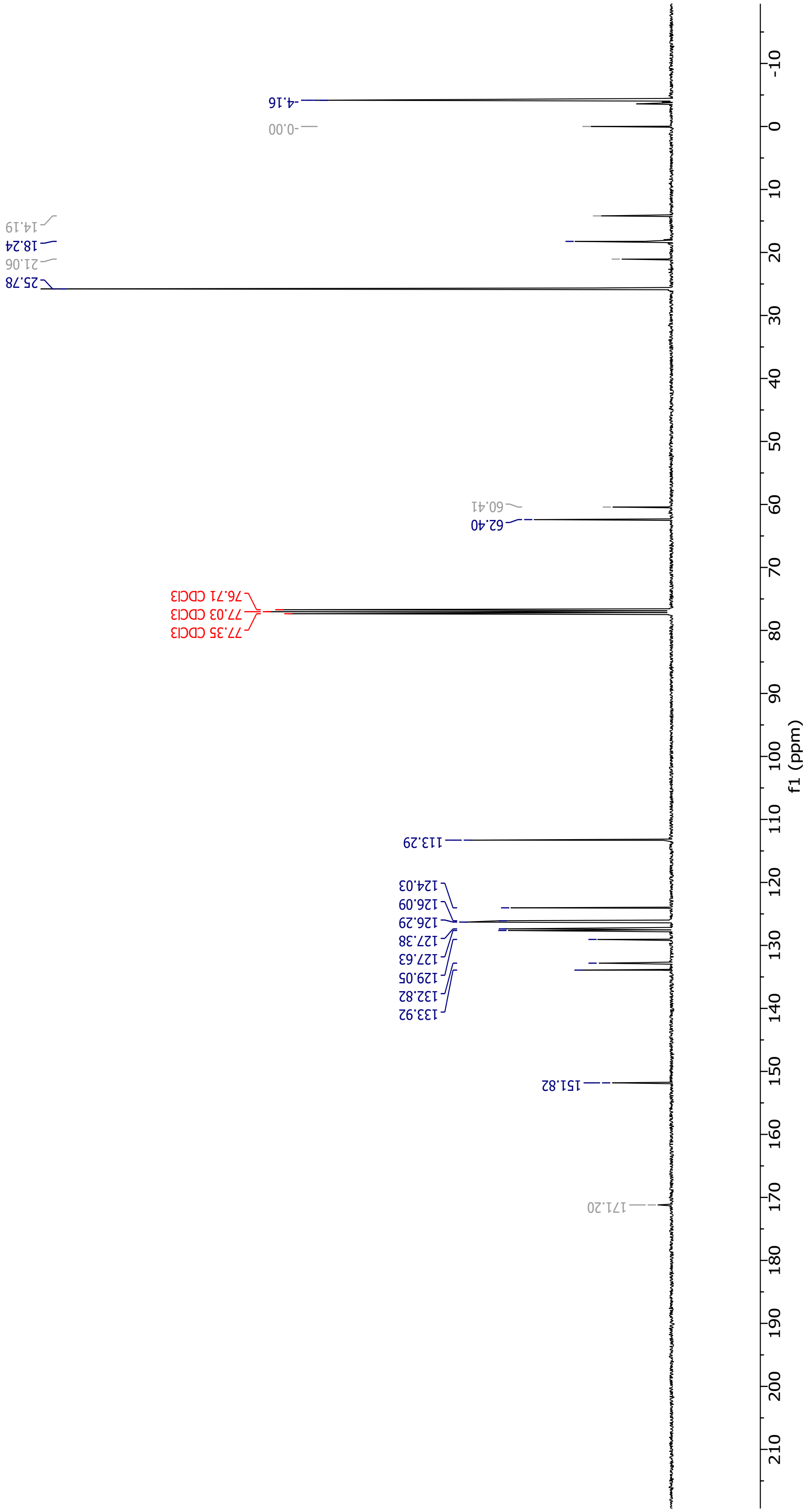

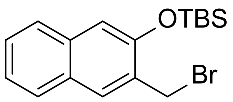

6

^{1}H NMR (400 MHz, $CDCl_3$)

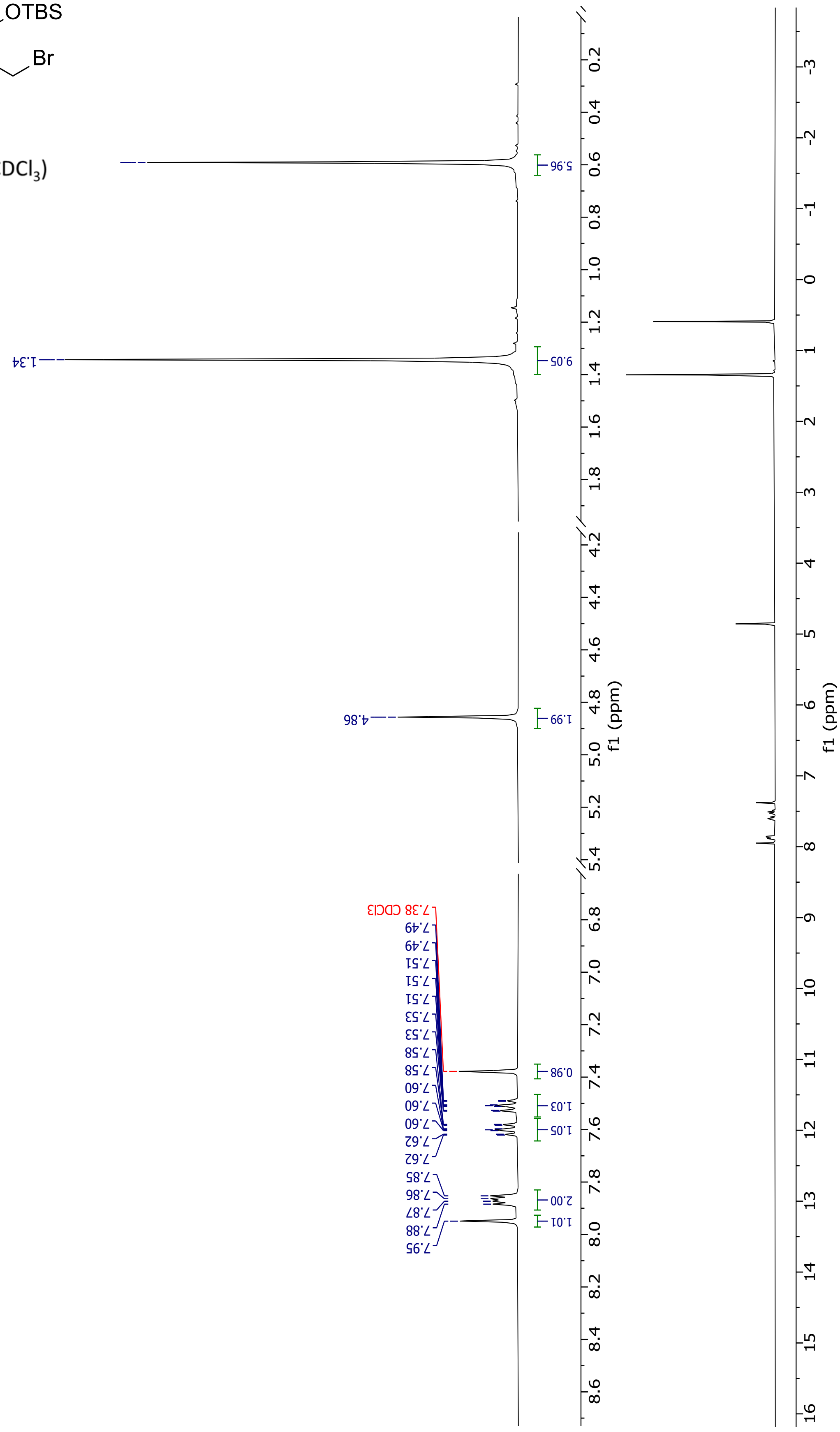

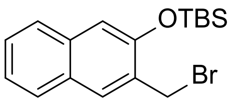

6

$^{13}C\{^{1}H\}$ NMR (101 MHz, $CDCl_3$)

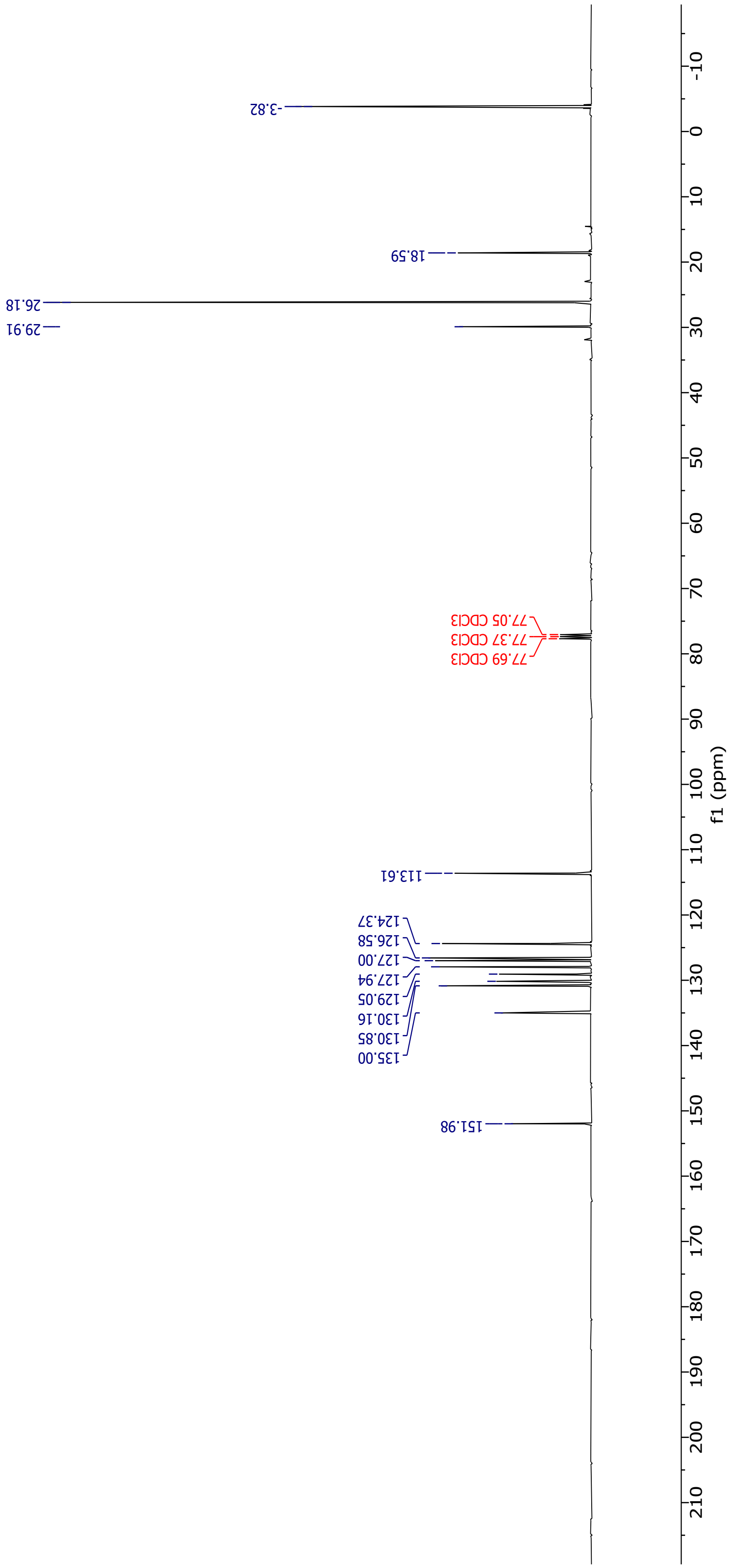

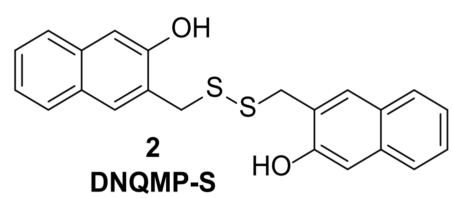

2
DNQMP-S

^{1}H NMR (400 MHz, CD_3OD)

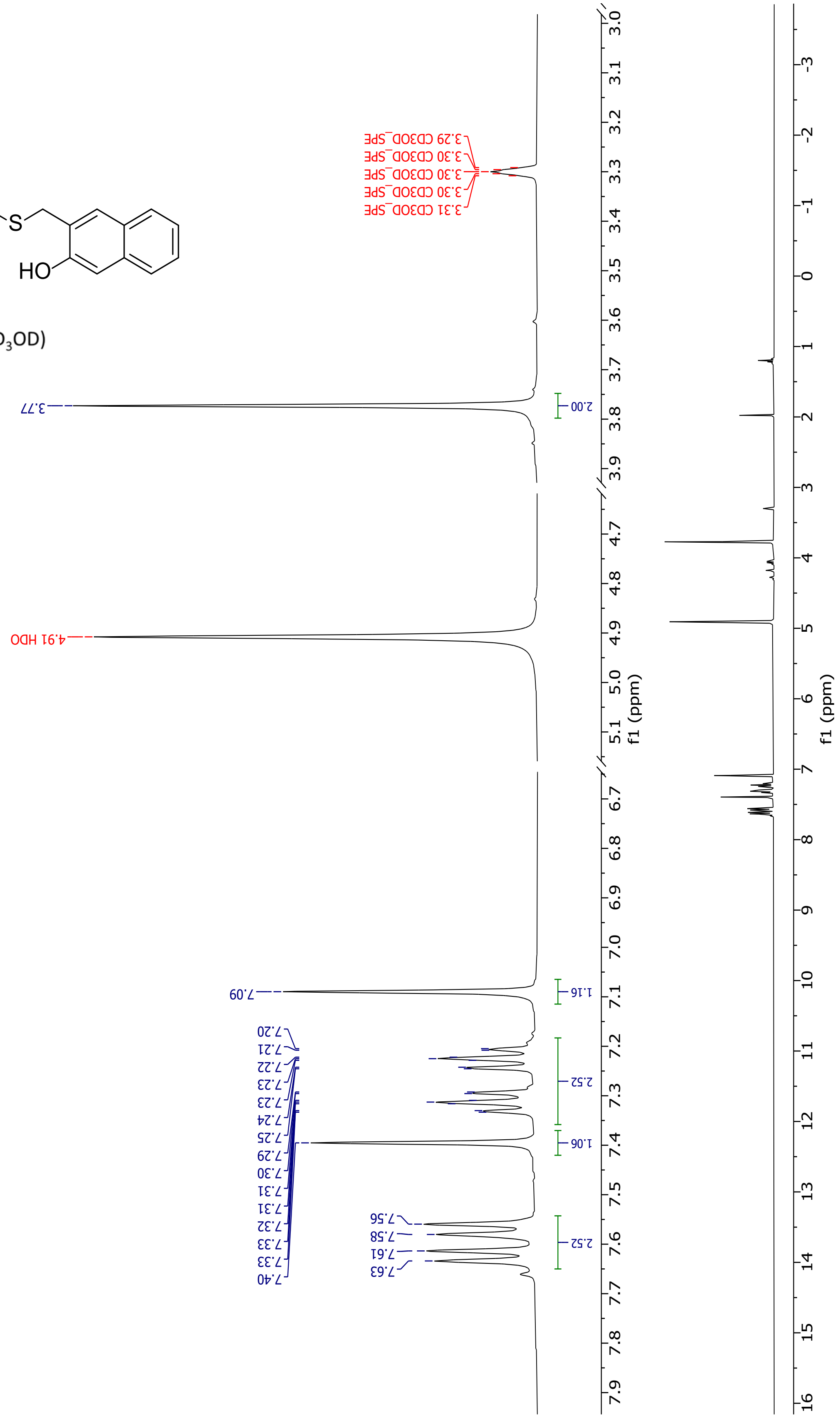

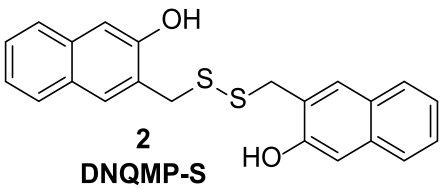

2
DNQMP-S

$^{13}C\{^{1}H\}$ NMR (101 MHz, CD_3OD)

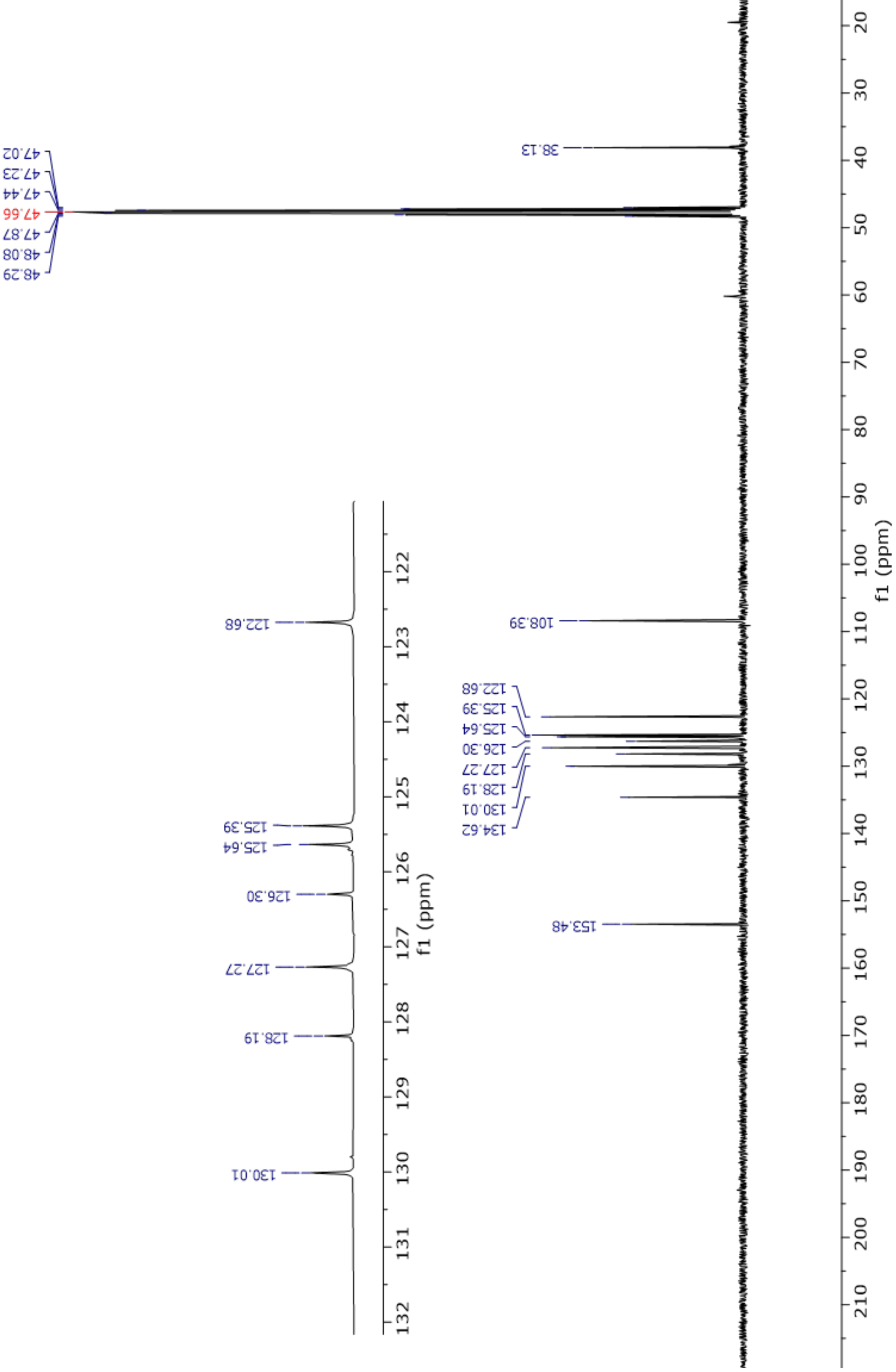

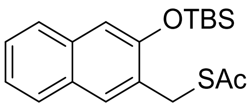

7

^{1}H NMR (400 MHz, $CDCl_3$)

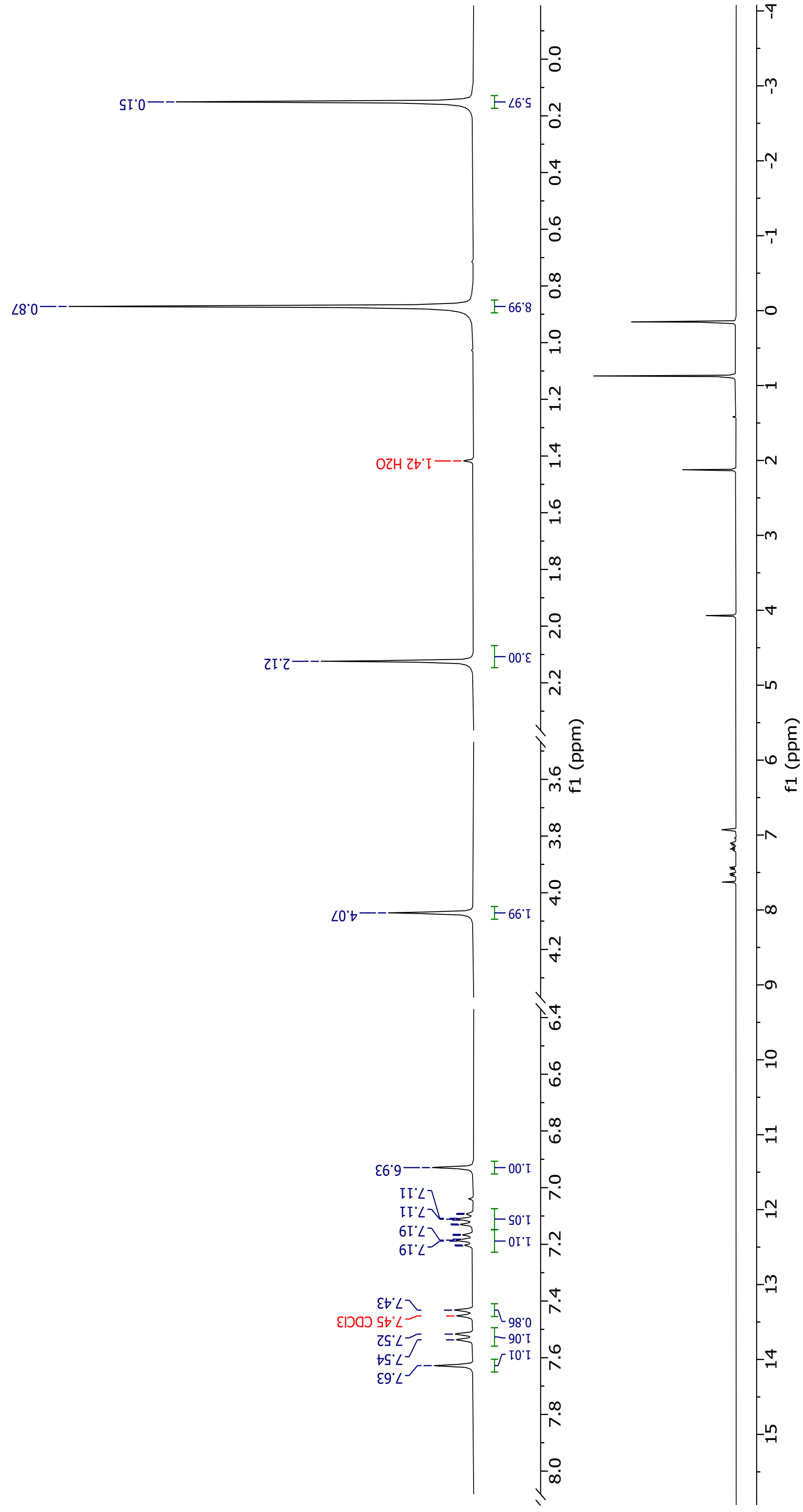

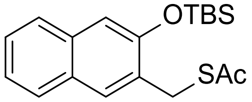

7

$^{13}C\{^{1}H\}$ NMR (101 MHz, $CDCl_3$)

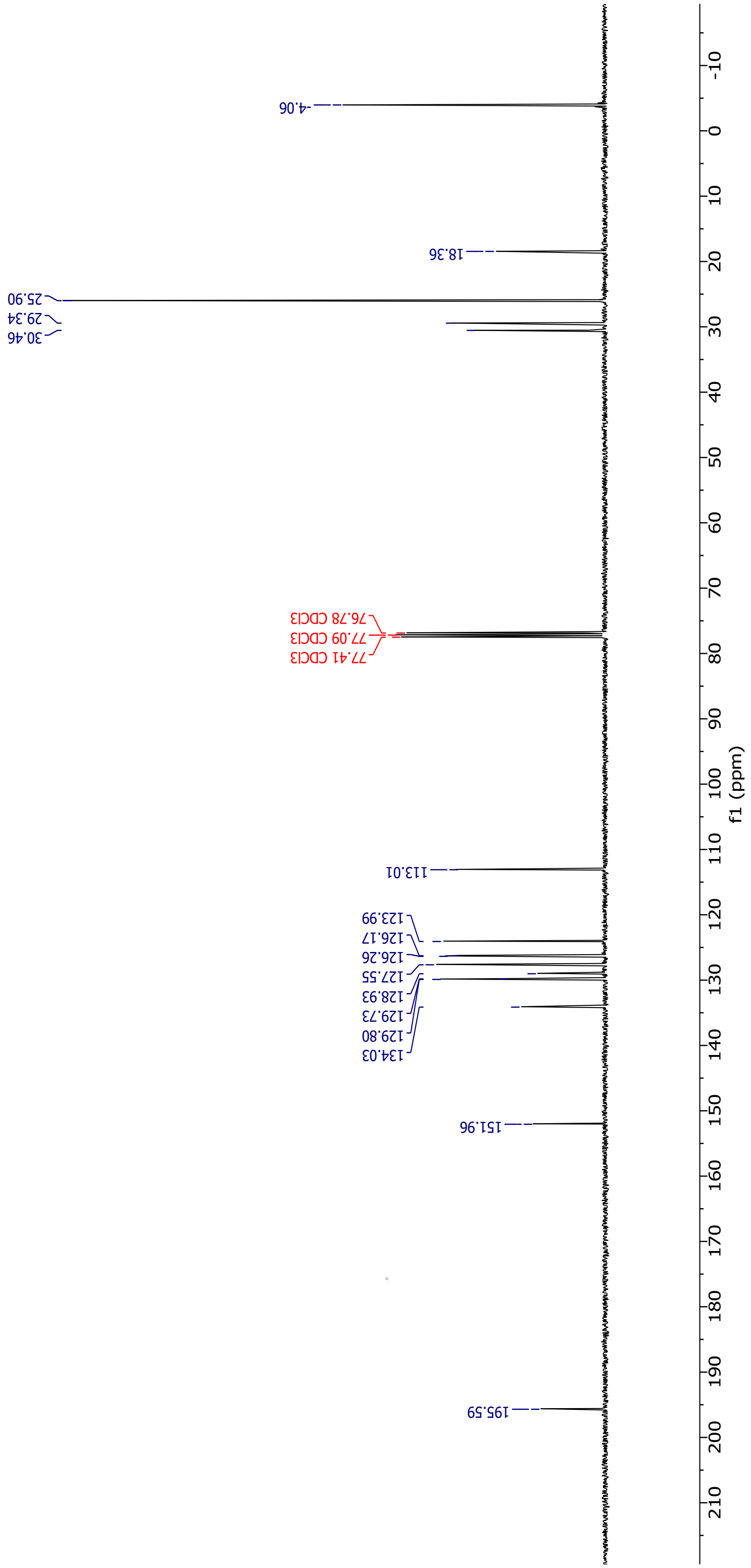

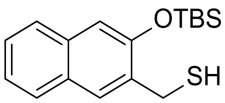

8

^{1}H NMR (400 MHz, $CDCl_3$)

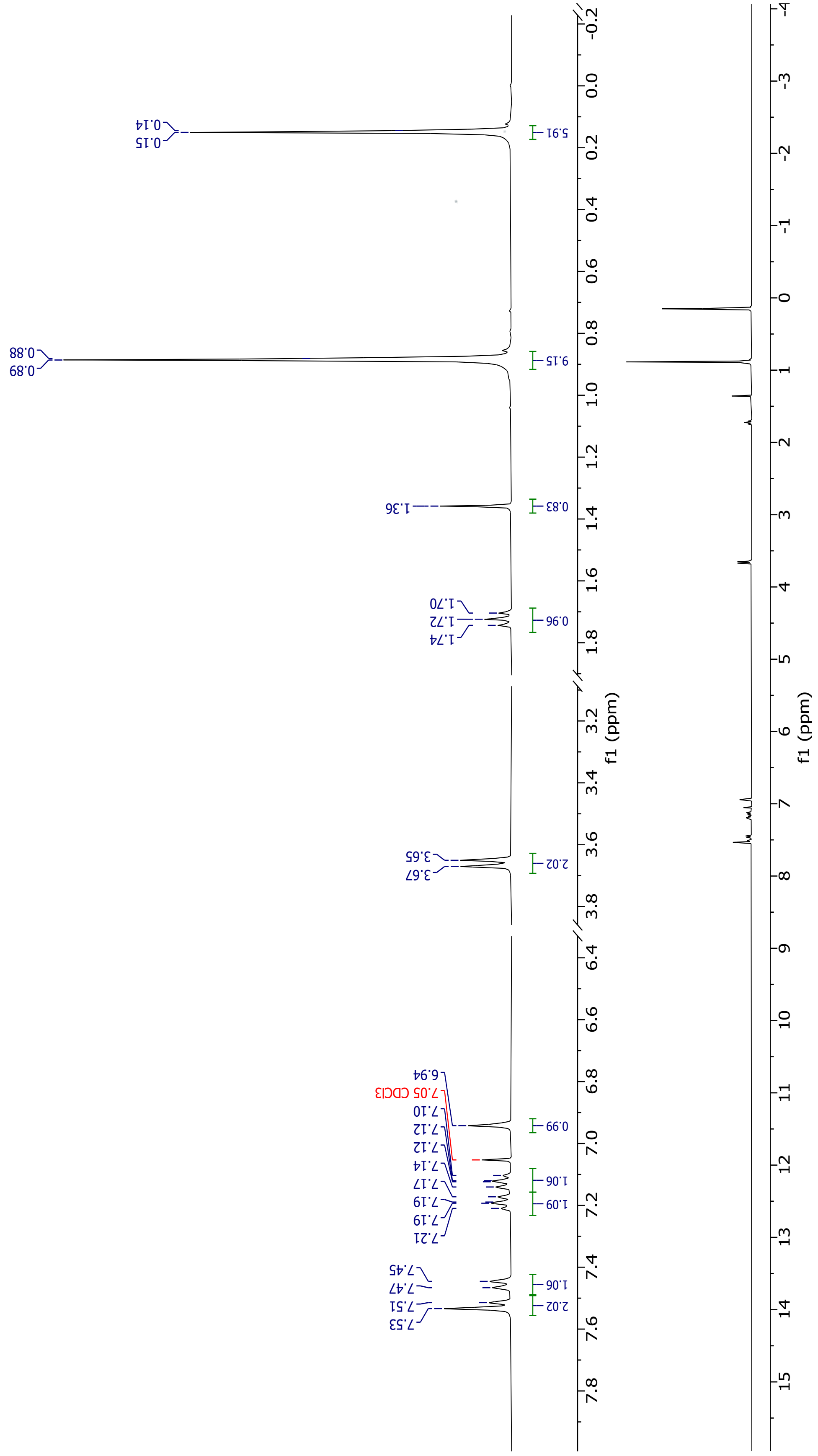

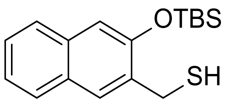

8

$^{13}C\{^{1}H\}$ NMR (101 MHz, $CDCl_3$)

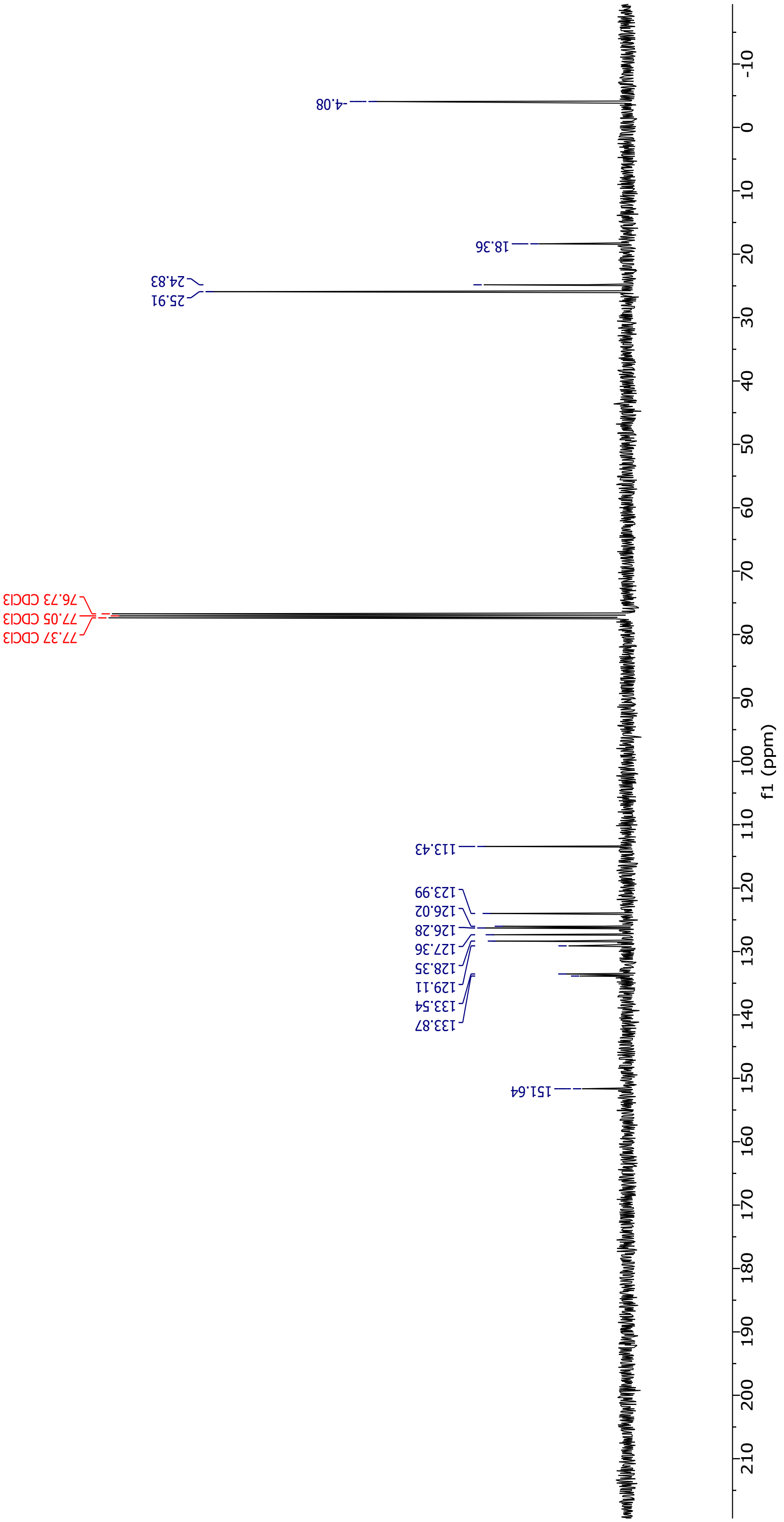

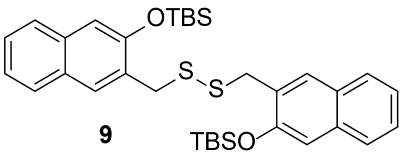

9

^{1}H NMR (400 MHz, $CDCl_3$)

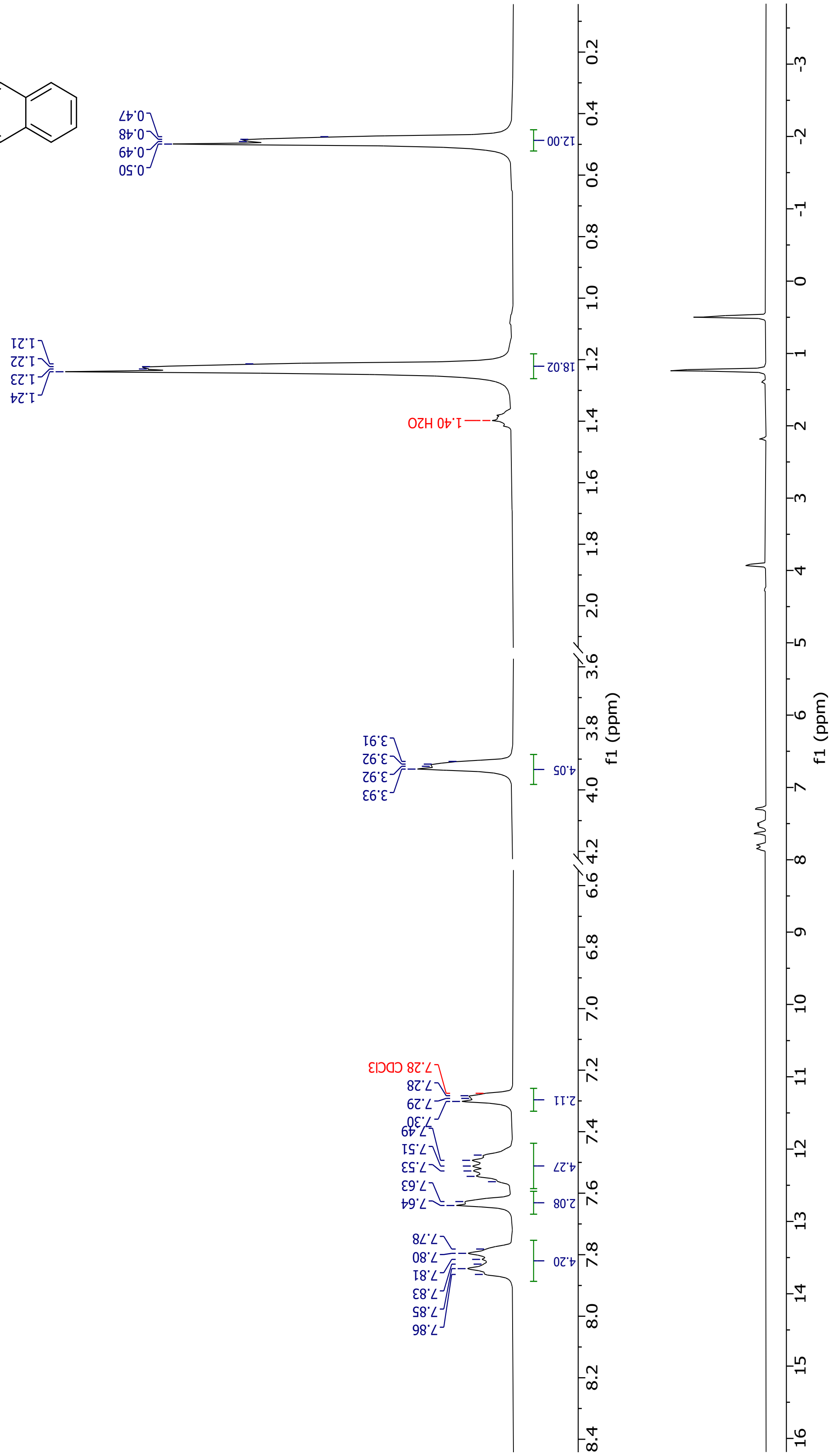

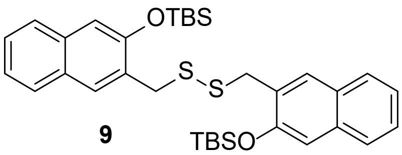

9

$^{13}C\{^{1}H\}$ NMR (101 MHz, $CDCl_3$)

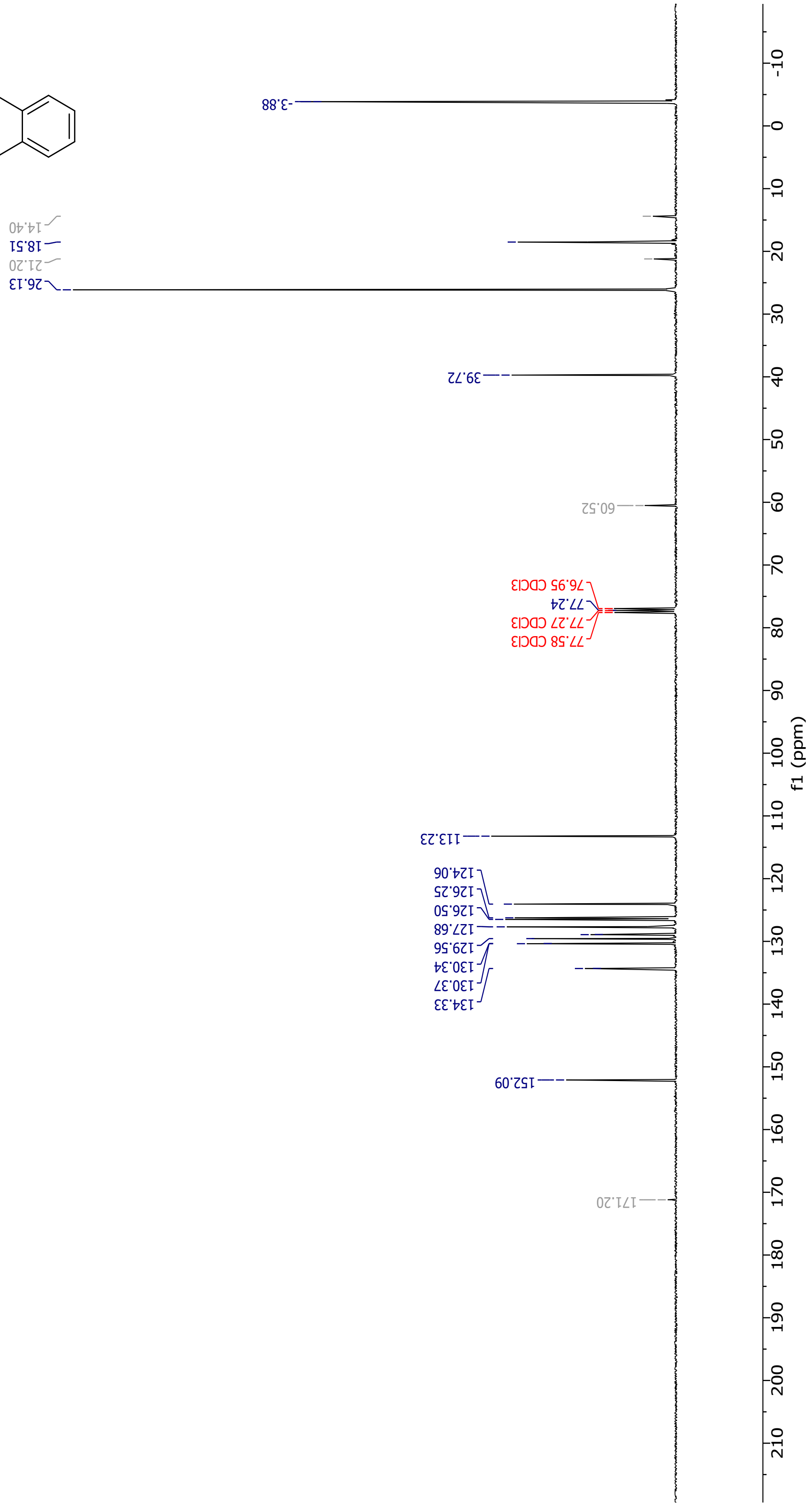

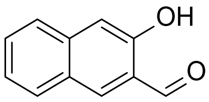

13

^{1}H NMR (400 MHz, $CDCl_3$)

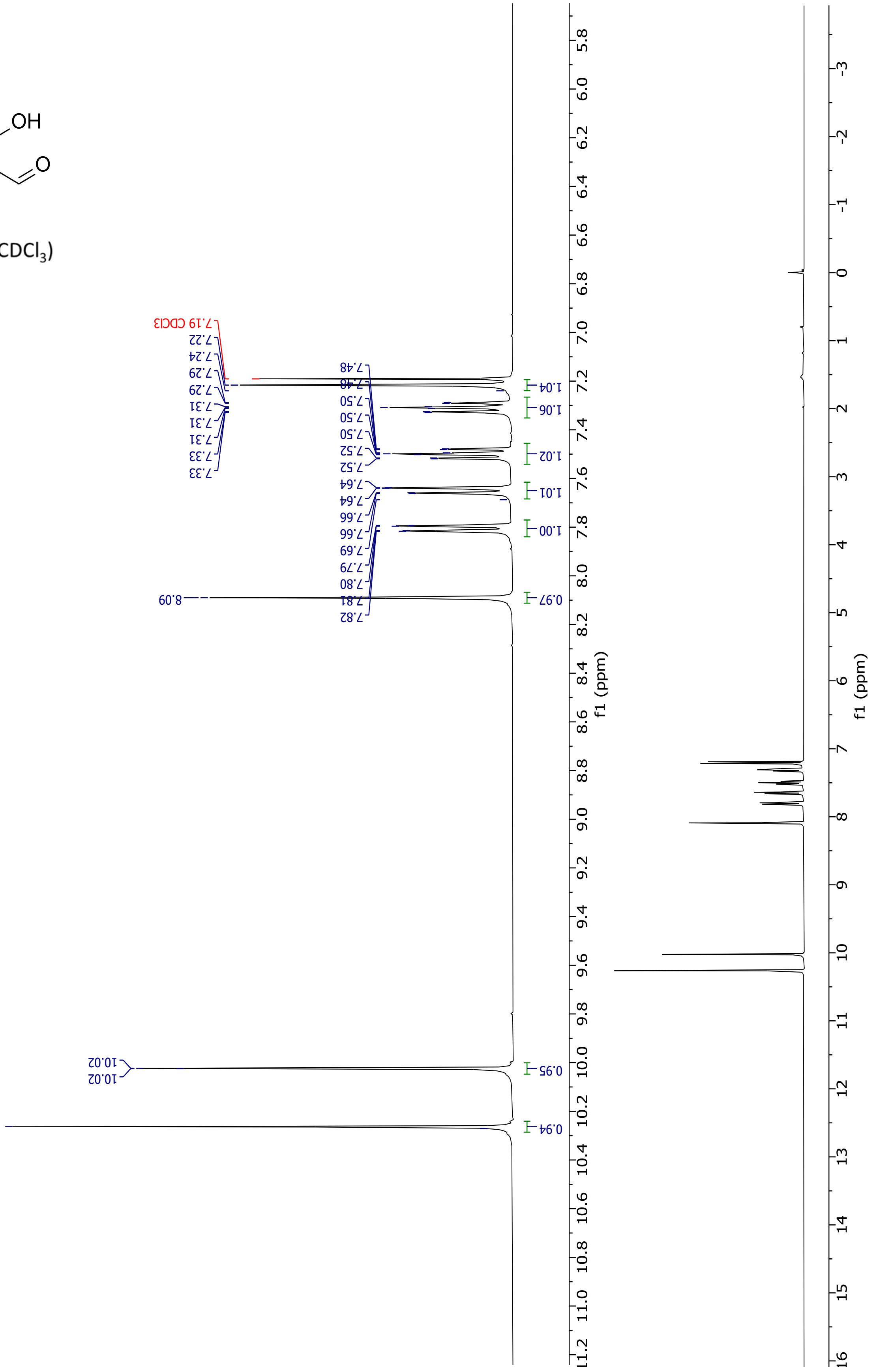

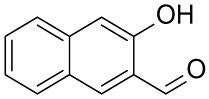

13

$^{13}C\{^{1}H\}$ NMR (101 MHz, $CDCl_3$)

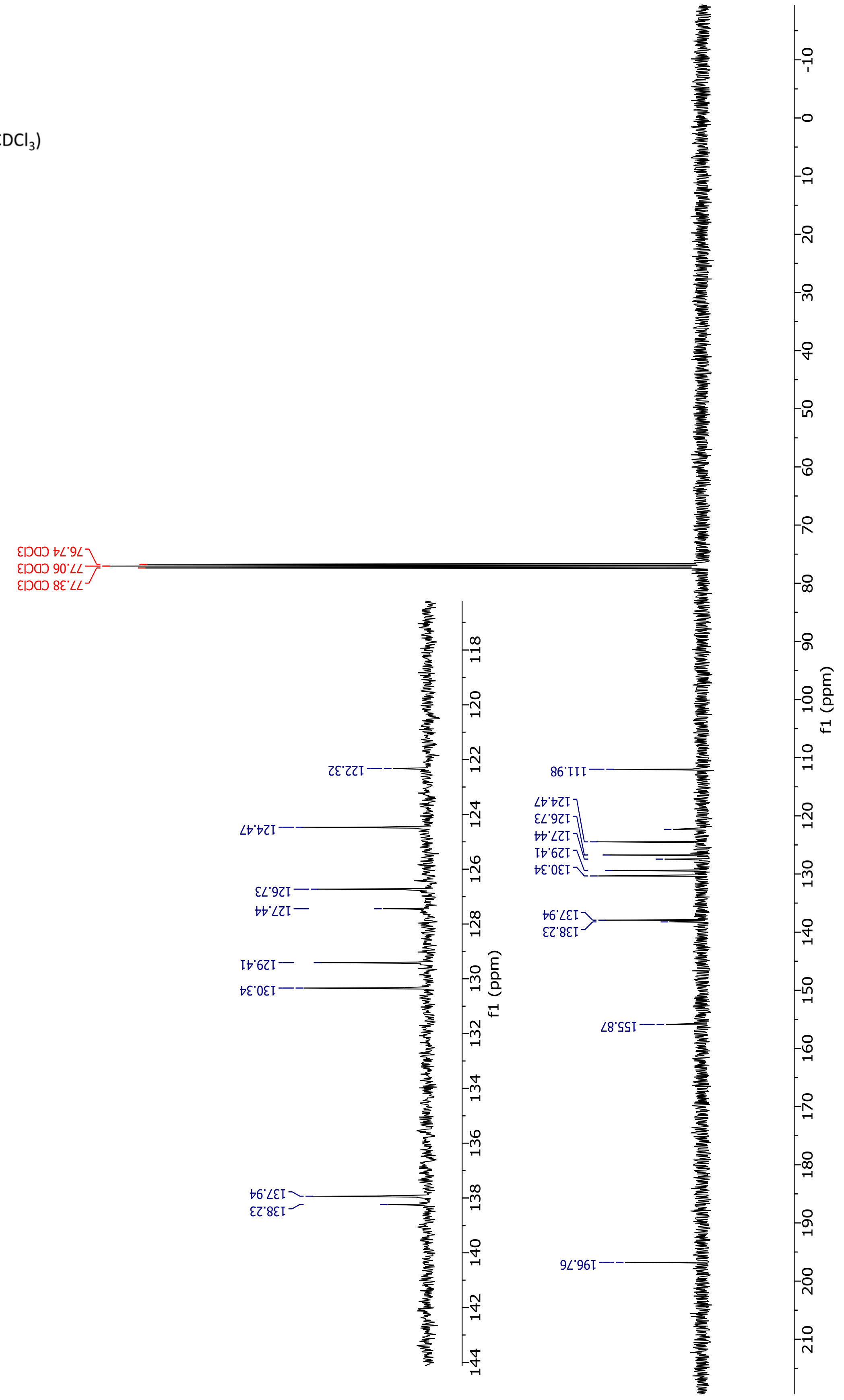

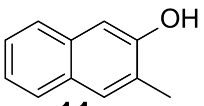

11

^{1}H NMR (400 MHz, $CDCl_3$)

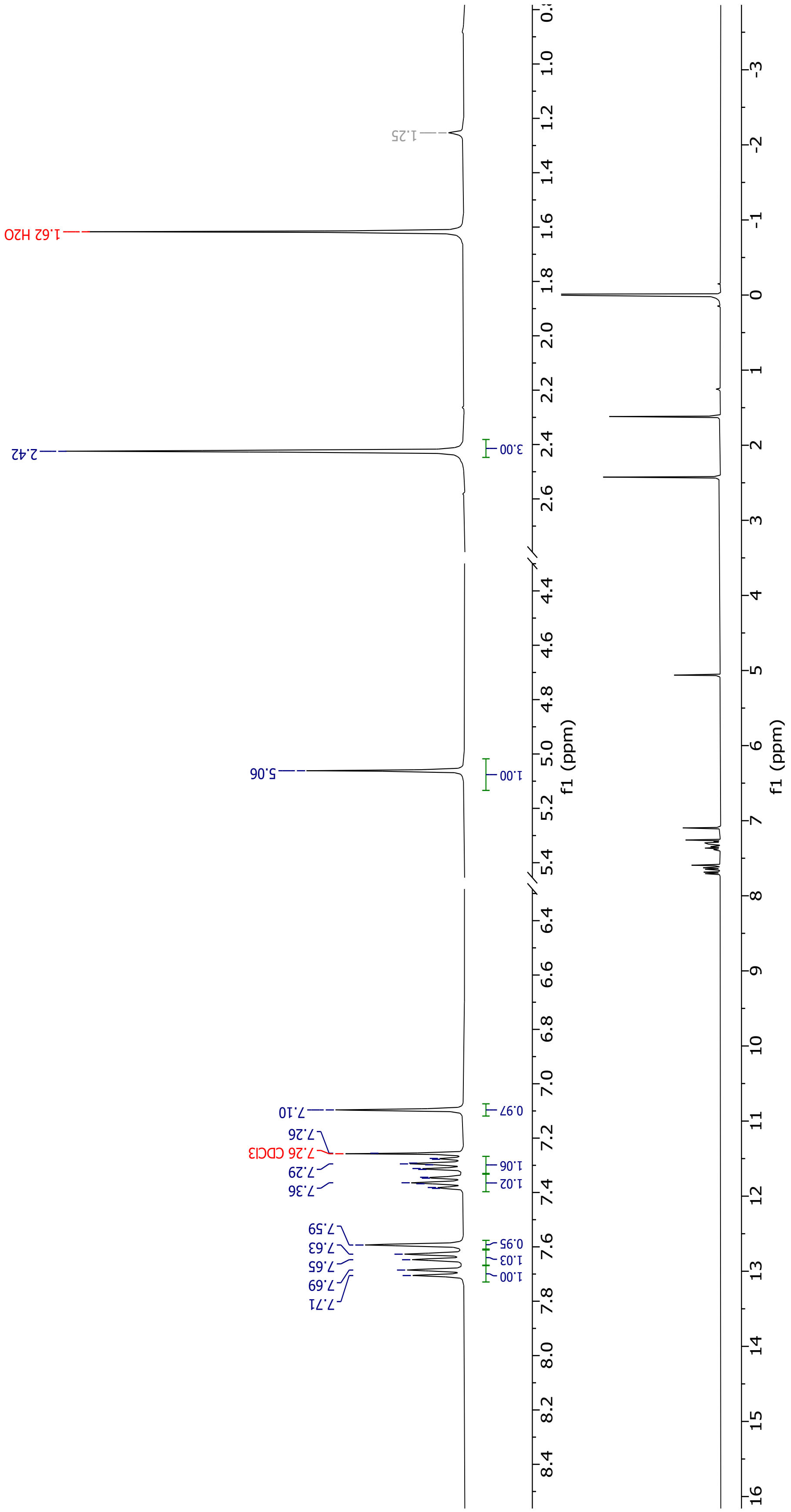

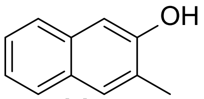

11

$^{13}C\{^{1}H\}$ NMR (101 MHz, $CDCl_3$)

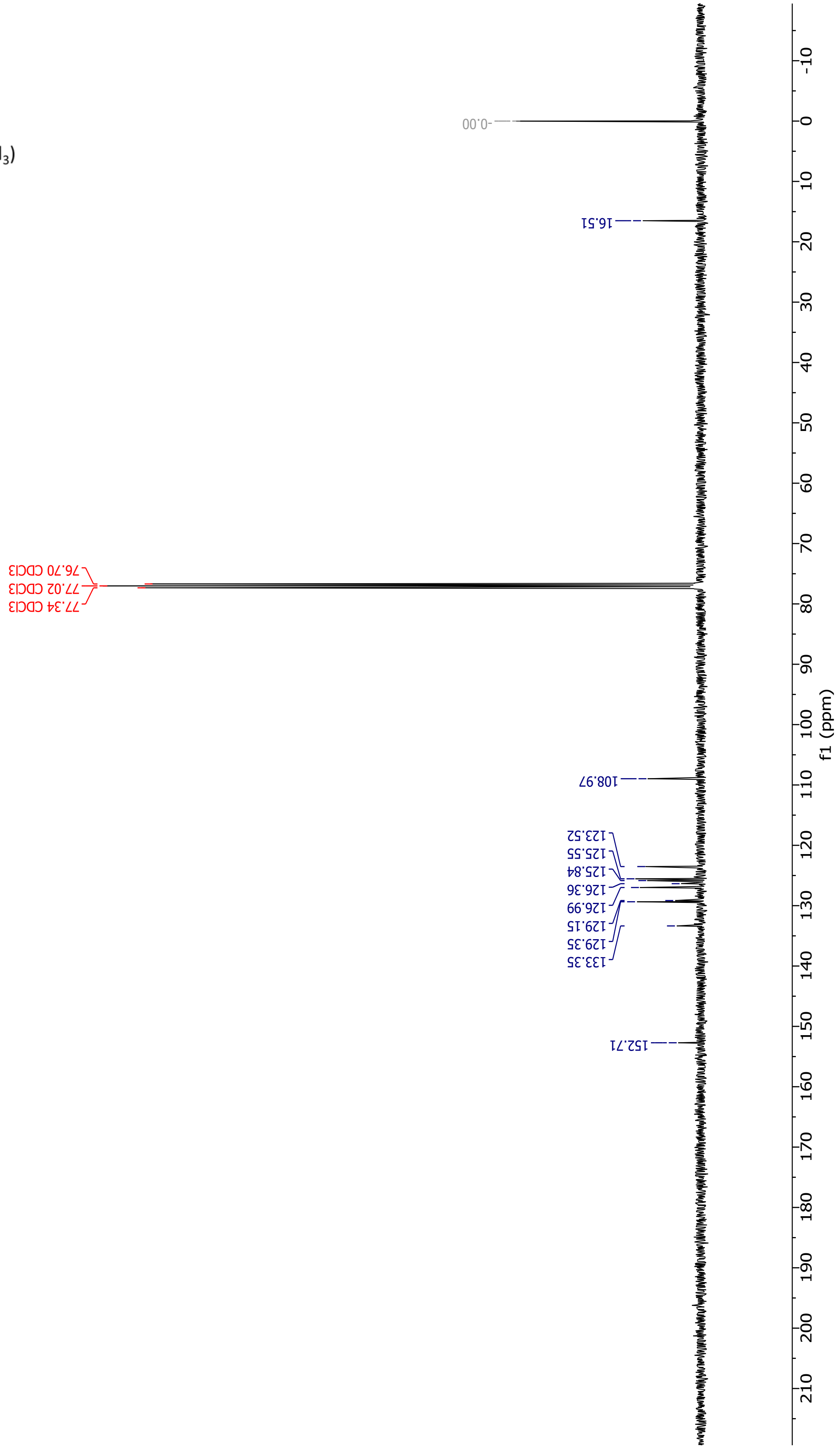

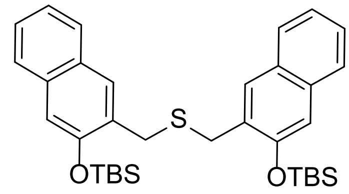

17

^1H NMR (400 MHz, $CDCl_3$)

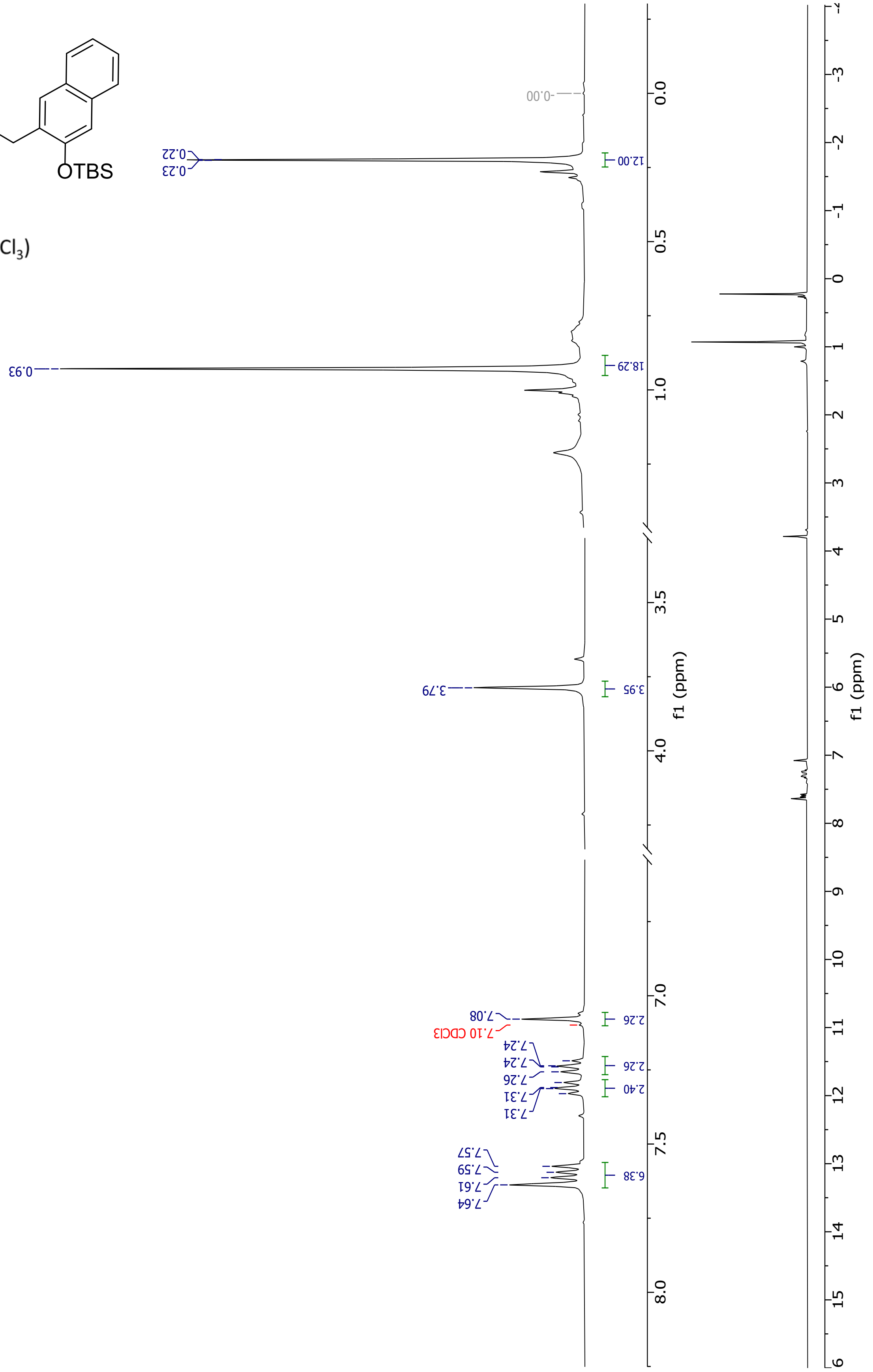

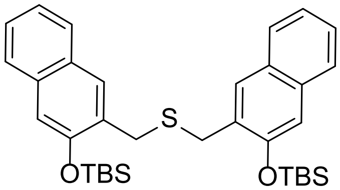

17

$^{13}C\{^{1}H\}$ NMR (101 MHz, $CDCl_3$)

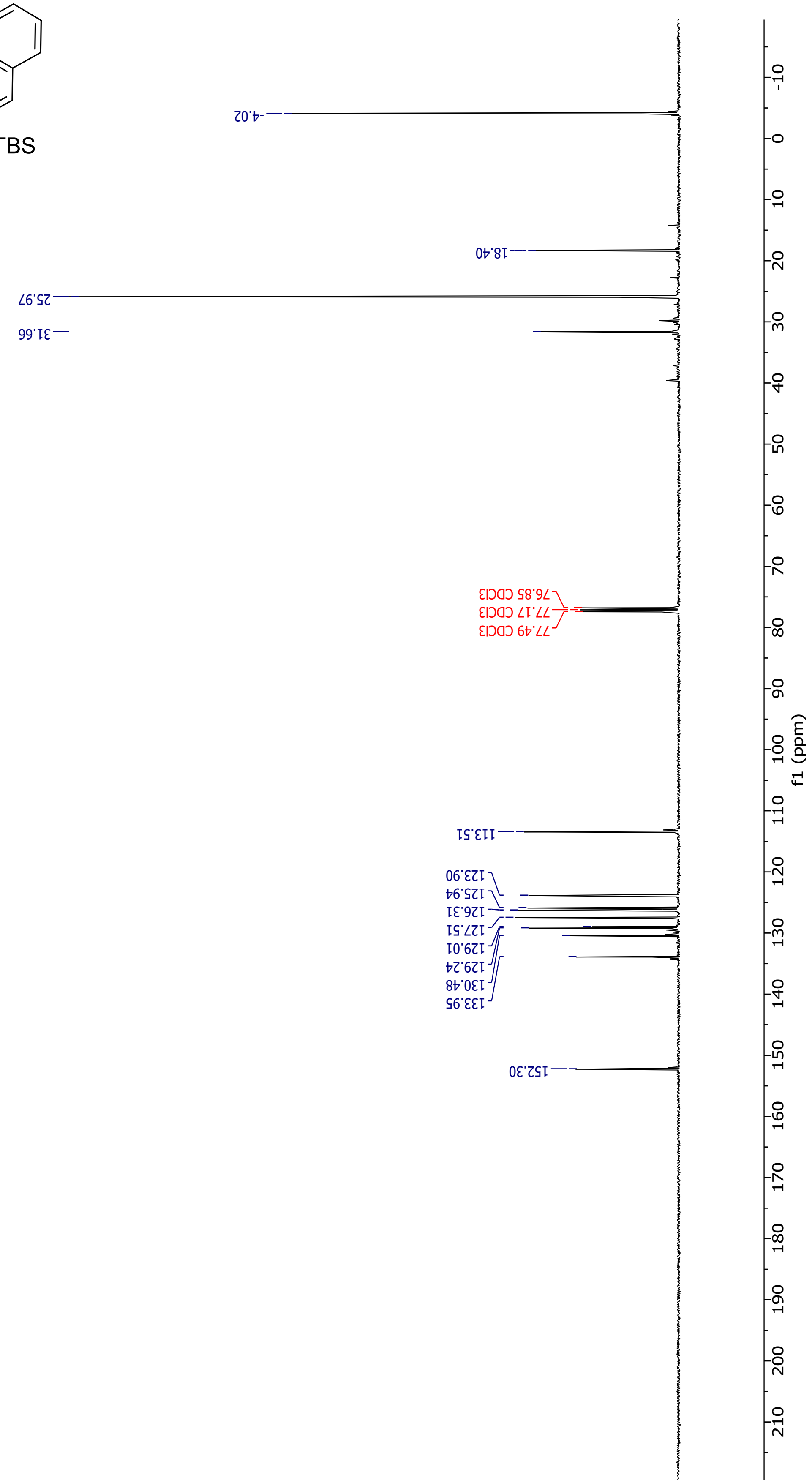

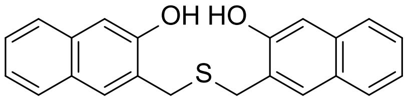

12

^{1}H NMR (400 MHz, $CDCl_3$)

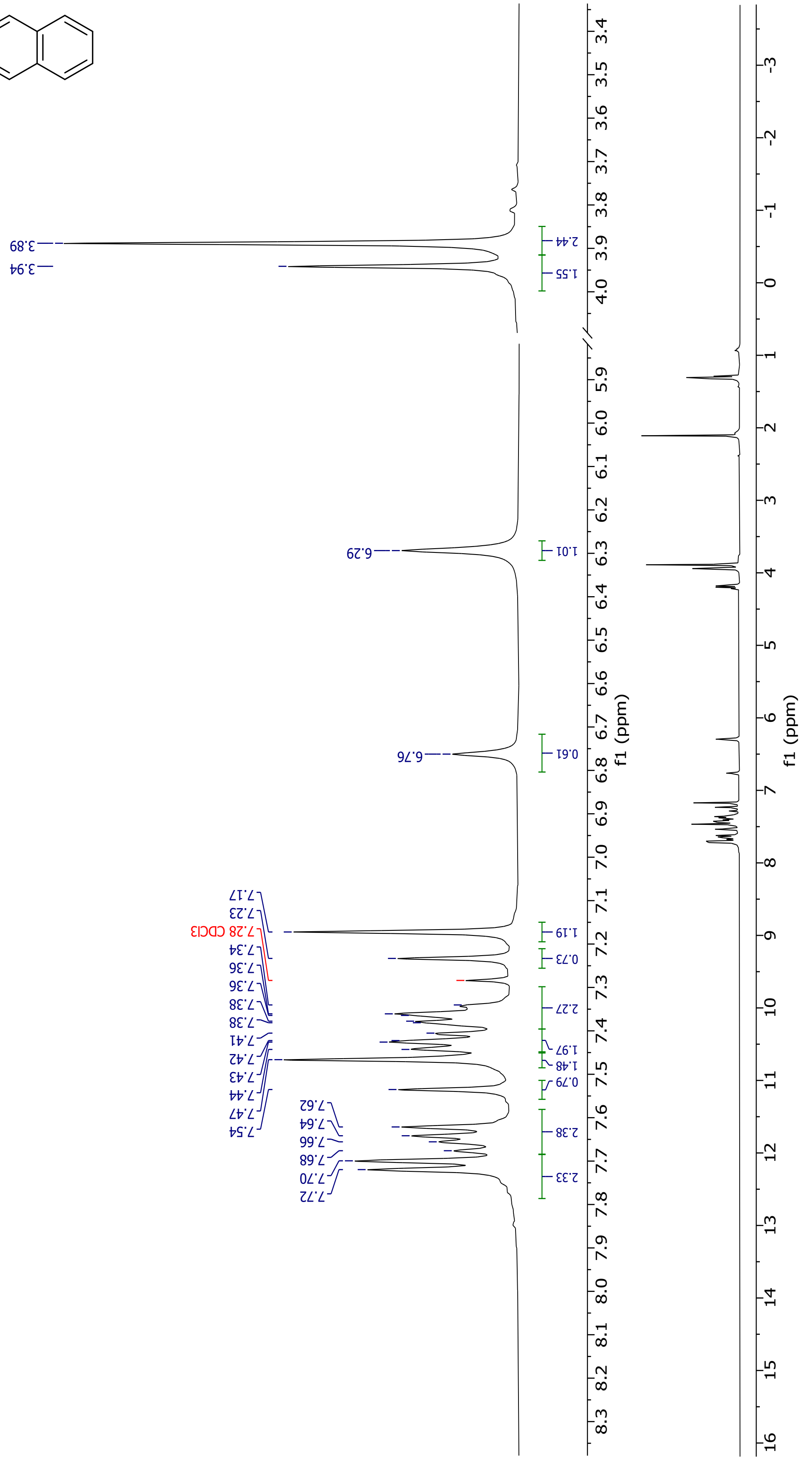

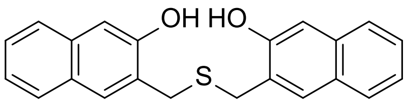

12

$^{13}C\{^{1}H\}$ NMR (101 MHz, $CDCl_3$)

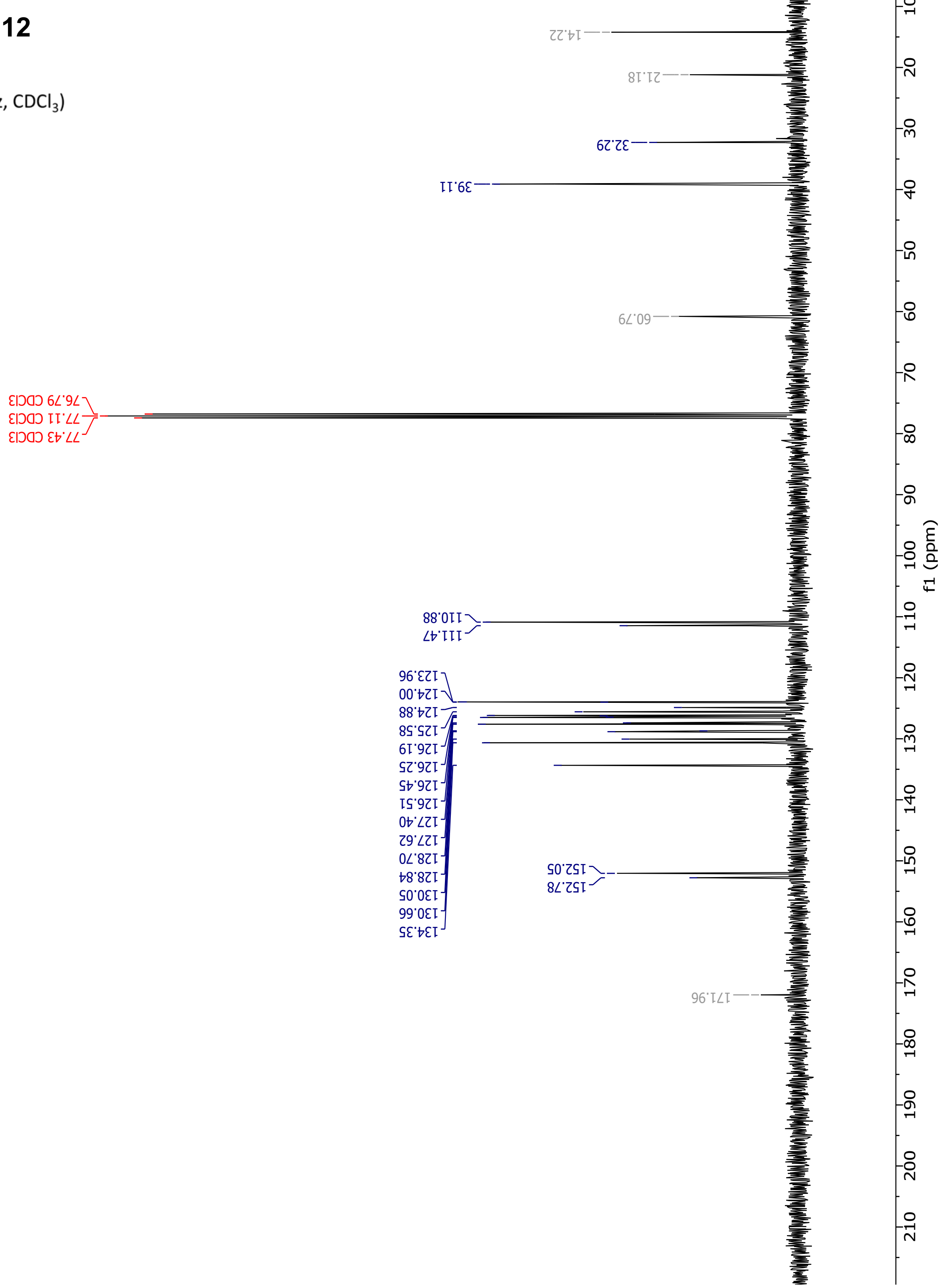

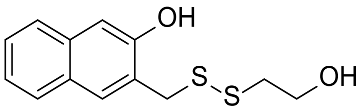

15c

^{1}H NMR (400 MHz, $CDCl_3$)

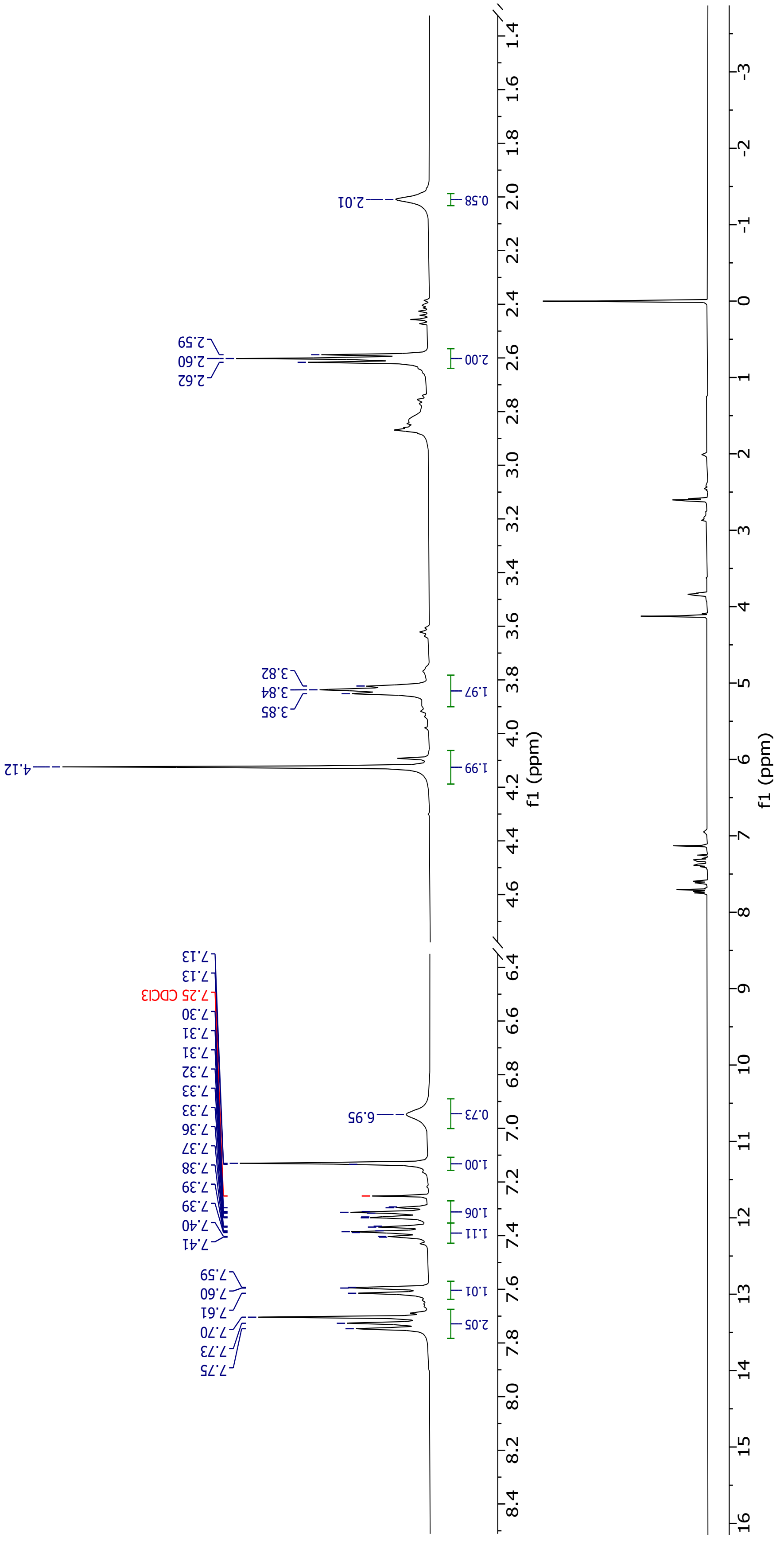

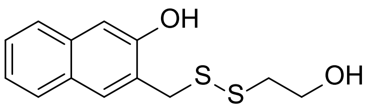

15c

$^{13}C\{^{1}H\}$ NMR (101 MHz, $CDCl_3$)

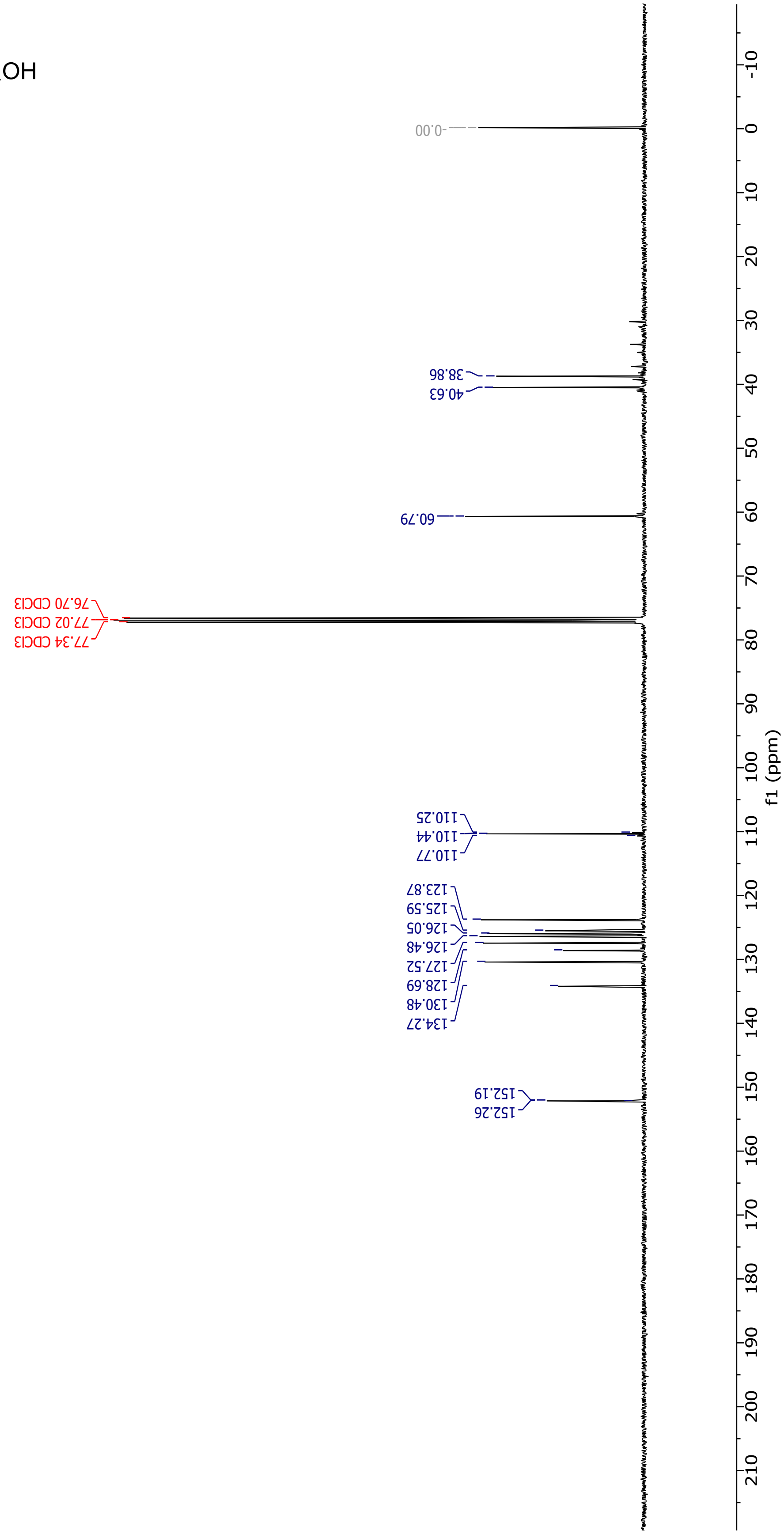

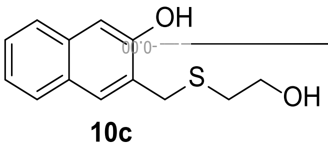

10c

^{1}H NMR (400 MHz, $CDCl_3$)

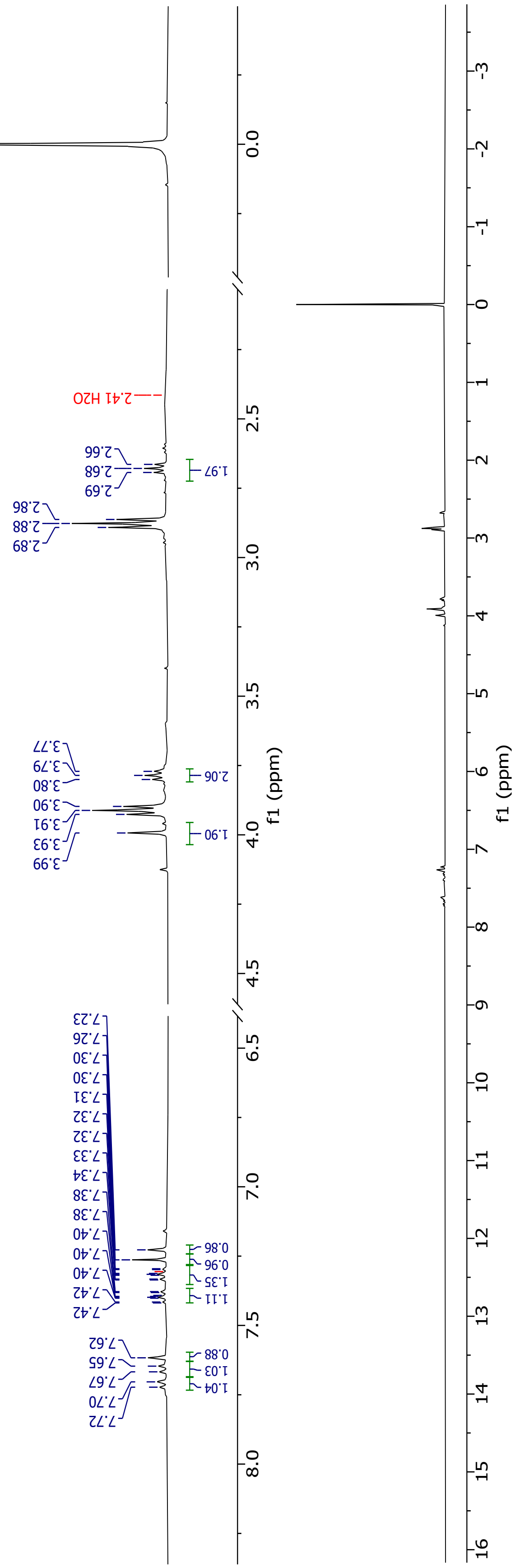

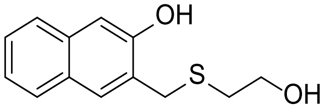

10c

$^{13}C\{^{1}H\}$ NMR (101 MHz, $CDCl_3$)

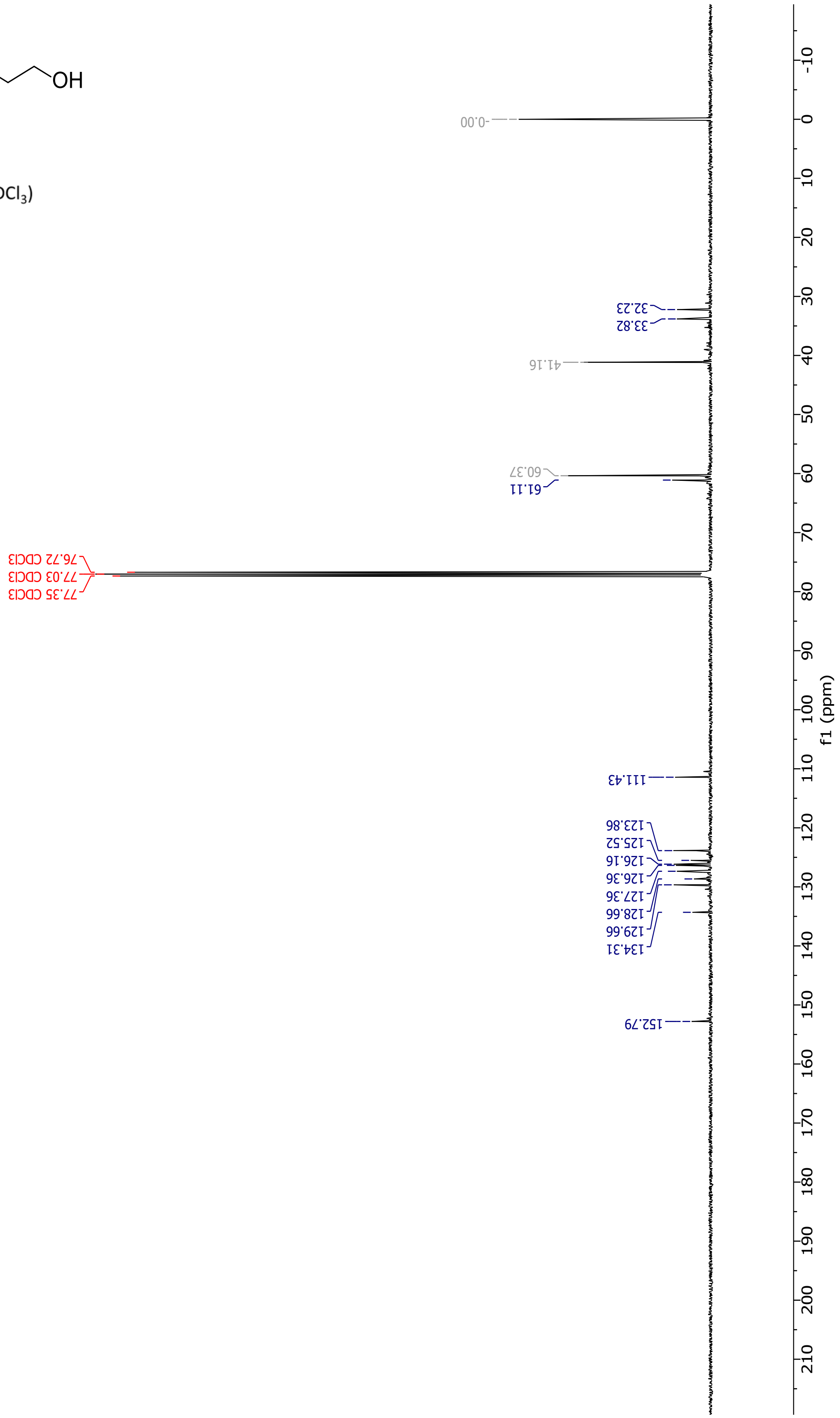

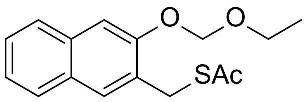

18

^{1}H NMR (400 MHz, $CDCl_3$)

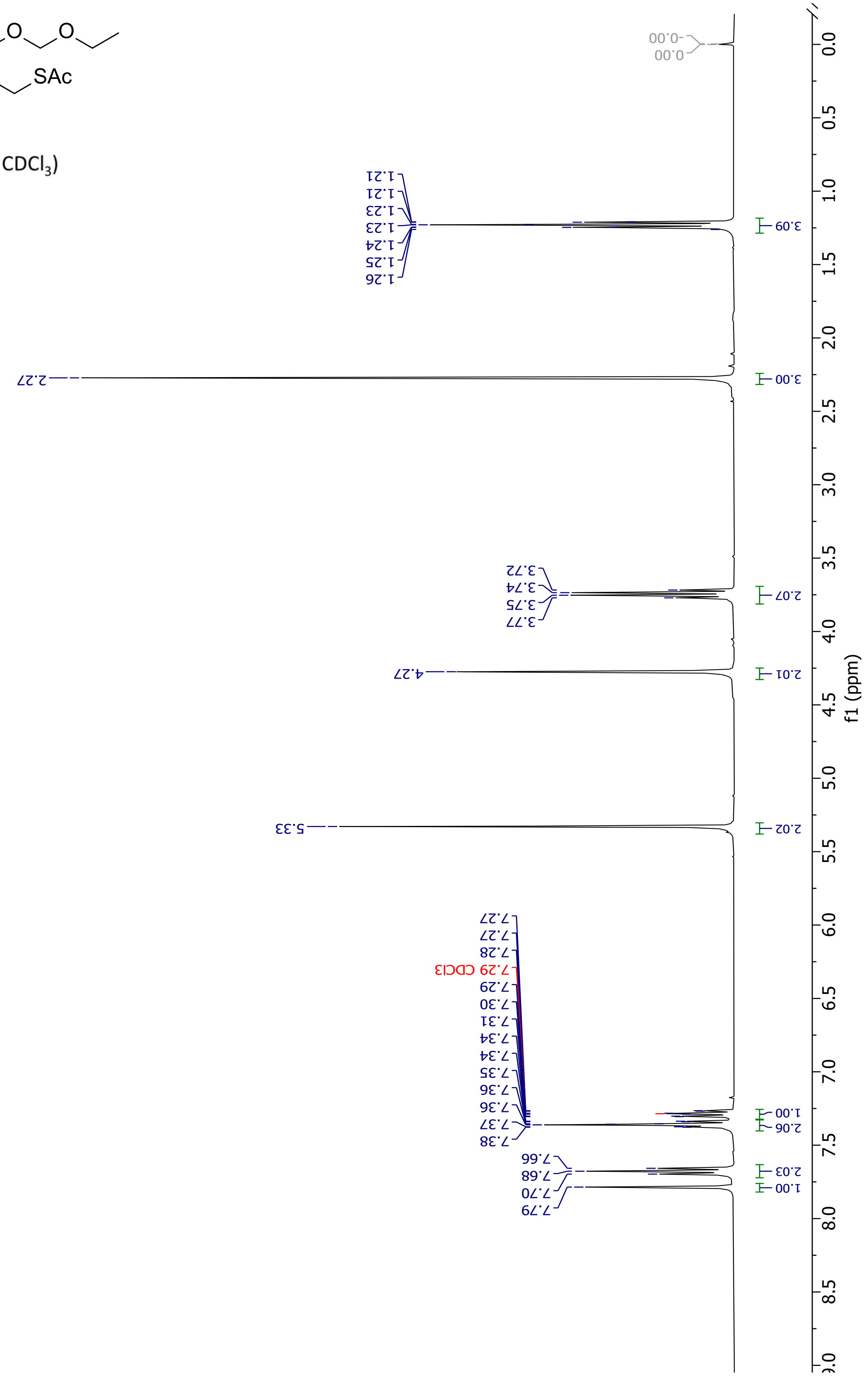

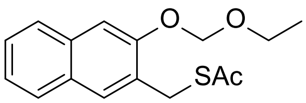

18

$^{13}C\{^1H\}$ NMR (101 MHz, $CDCl_3$)

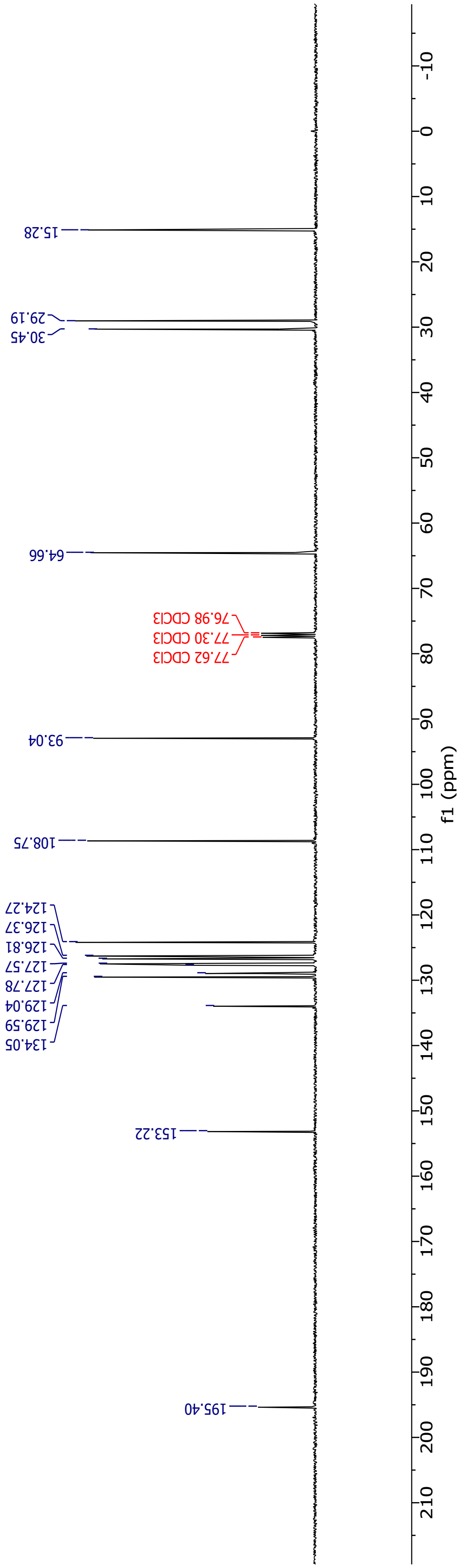

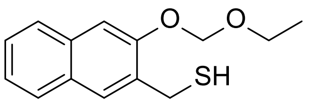

19

^{1}H NMR (400 MHz, $CDCl_3$)

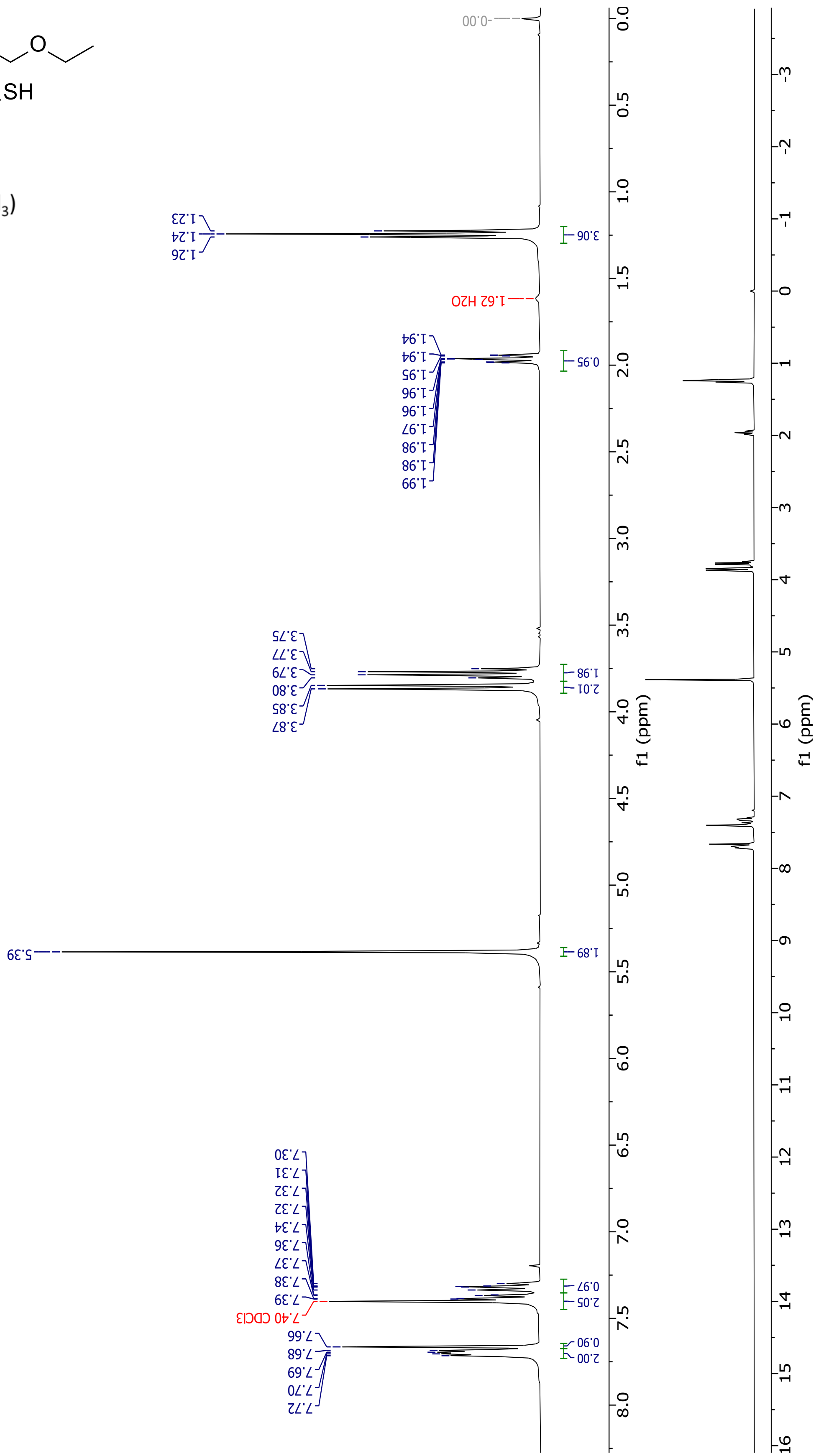

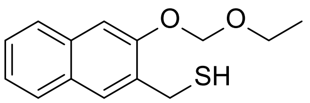

19

$^{13}C\{^1H\}$ NMR (101 MHz, $CDCl_3$)

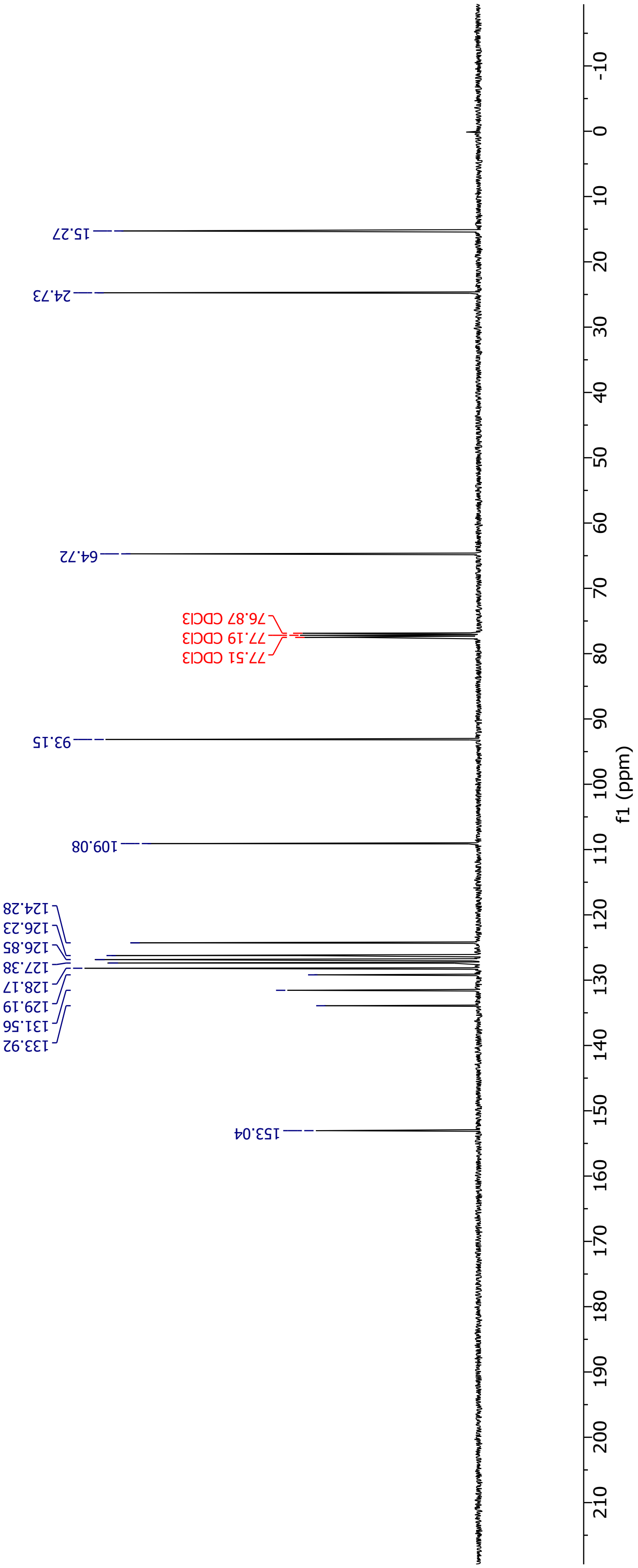

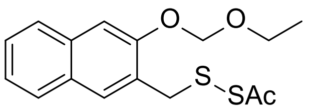

20

^{1}H NMR (400 MHz, $CDCl_3$)

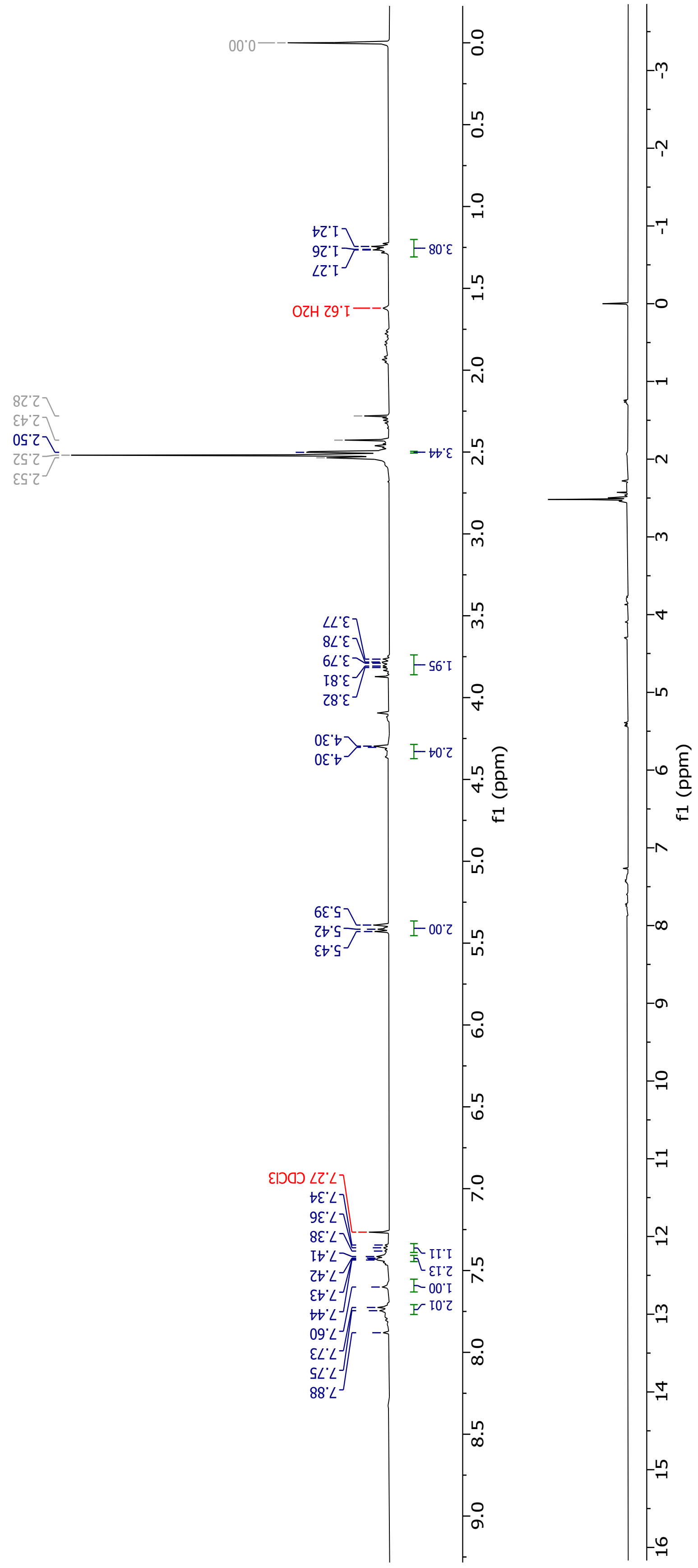

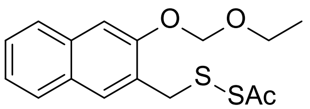

20

$^{13}C\{^1H\}$ NMR (101 MHz, $CDCl_3$)

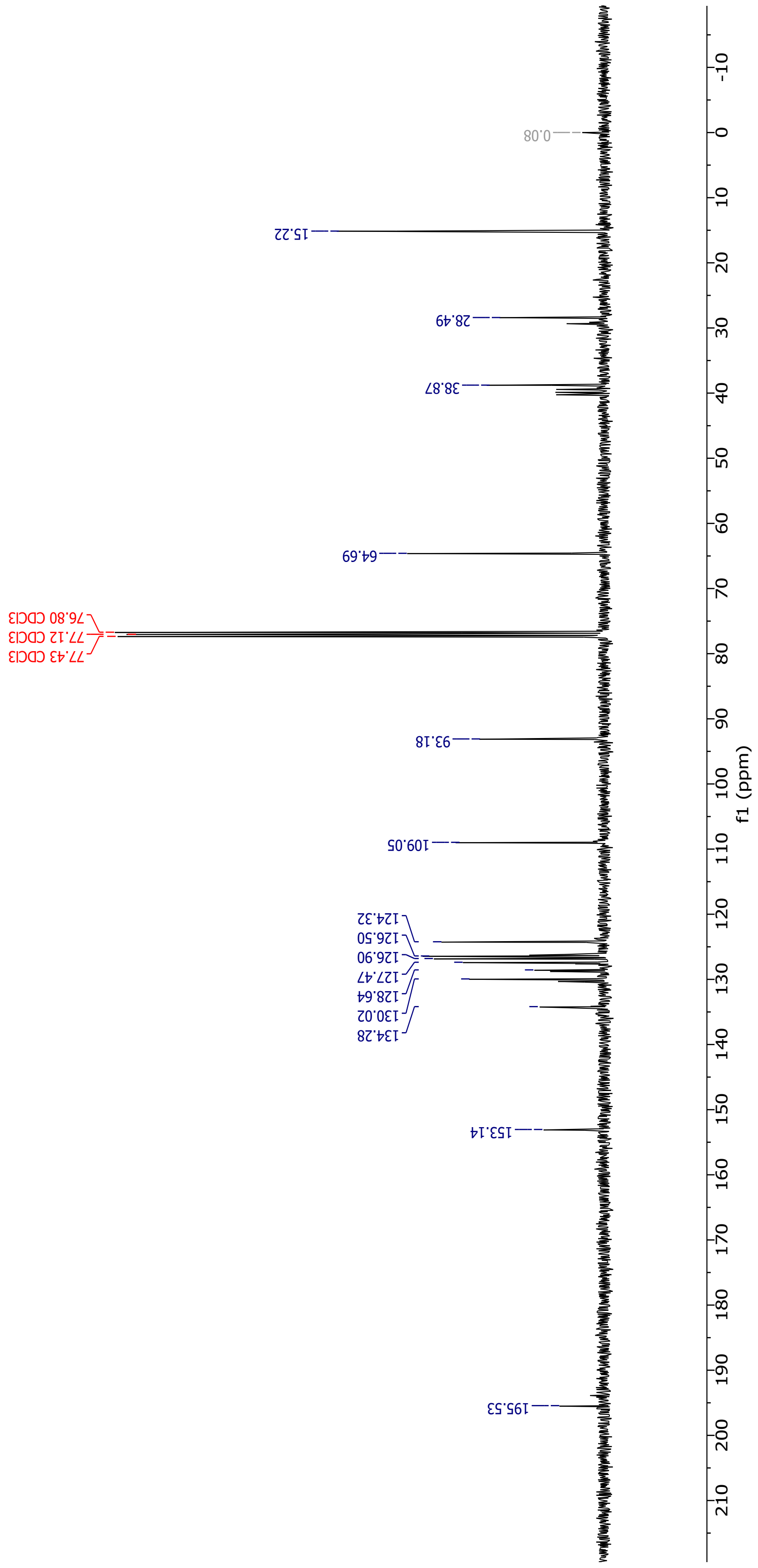

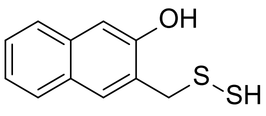

14

^{1}H NMR (400 MHz, $CDCl_3$)

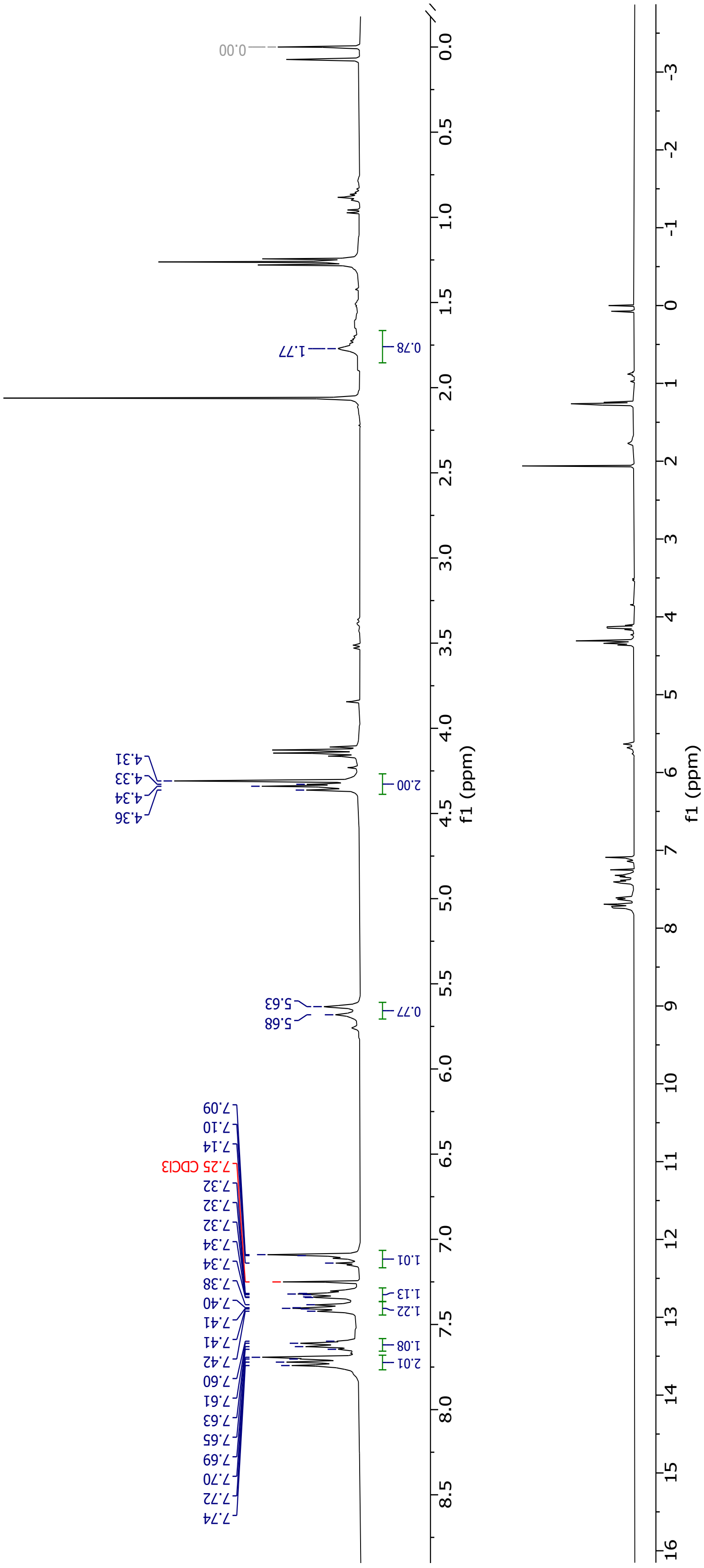

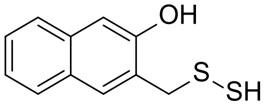

14

$^{13}C\{^{1}H\}$ NMR (101 MHz, $CDCl_3$)

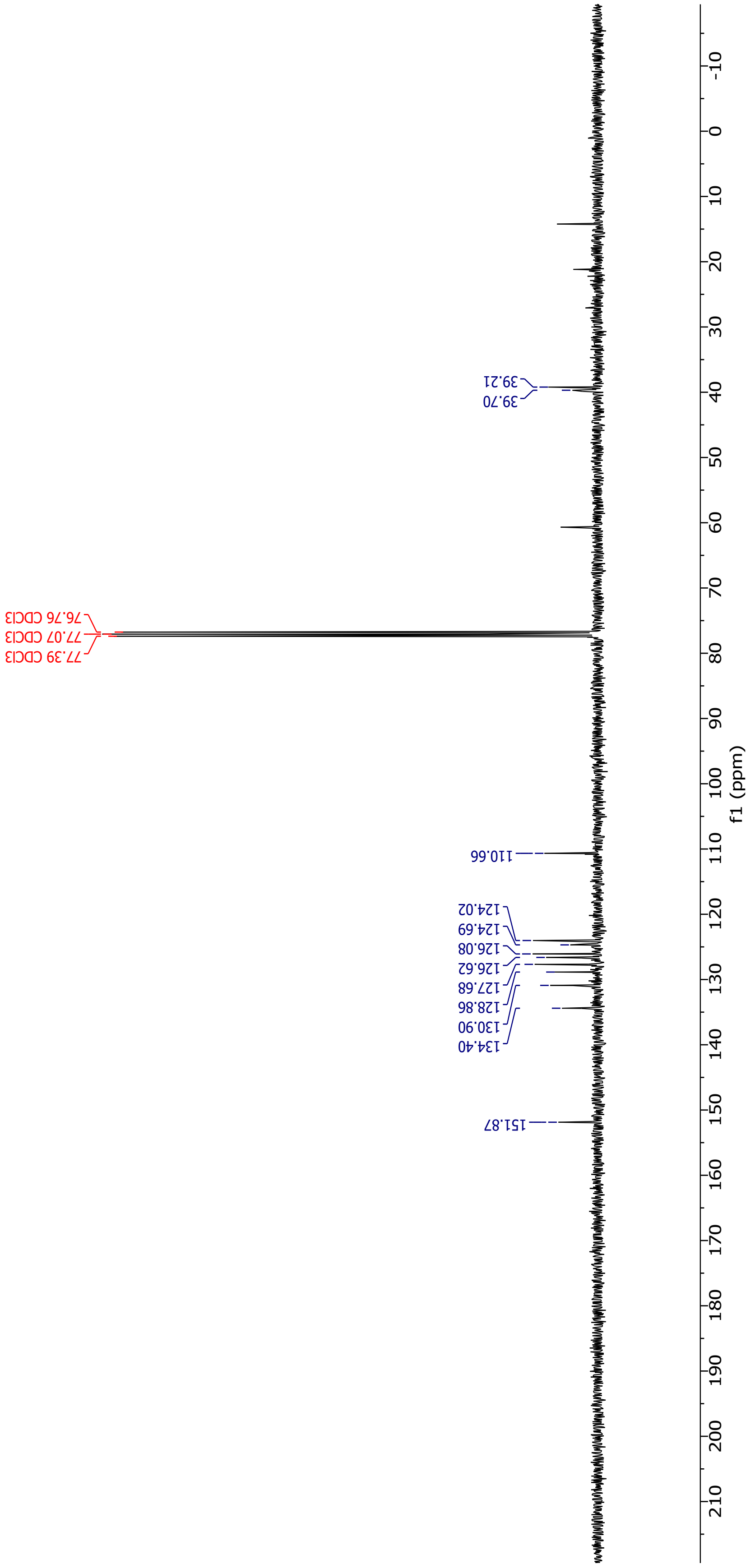

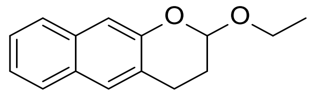

21

^{1}H NMR (400 MHz, $CDCl_3$)

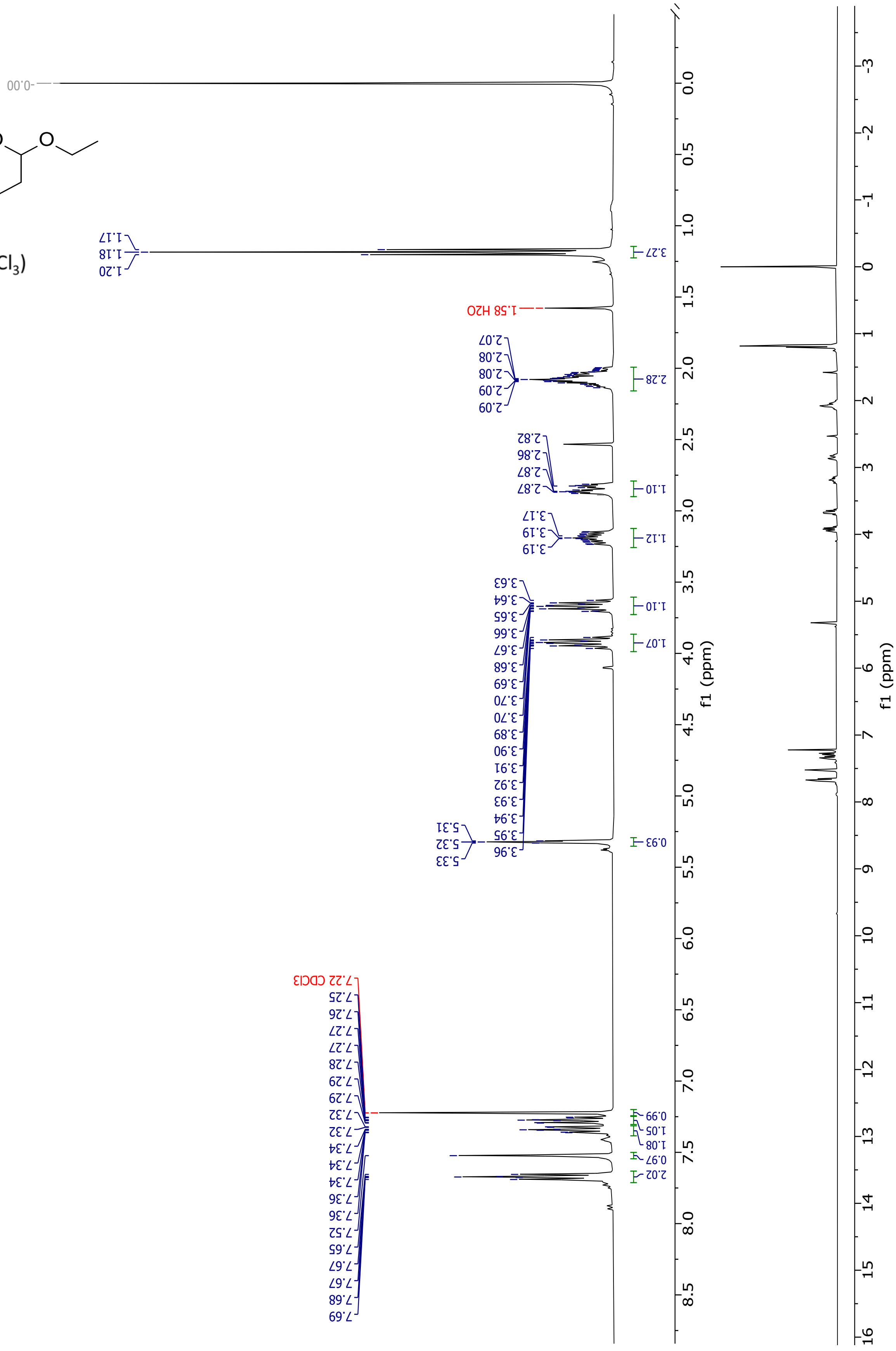

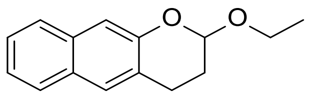

21

$^{13}C\{^{1}H\}$ NMR (101 MHz, $CDCl_3$)

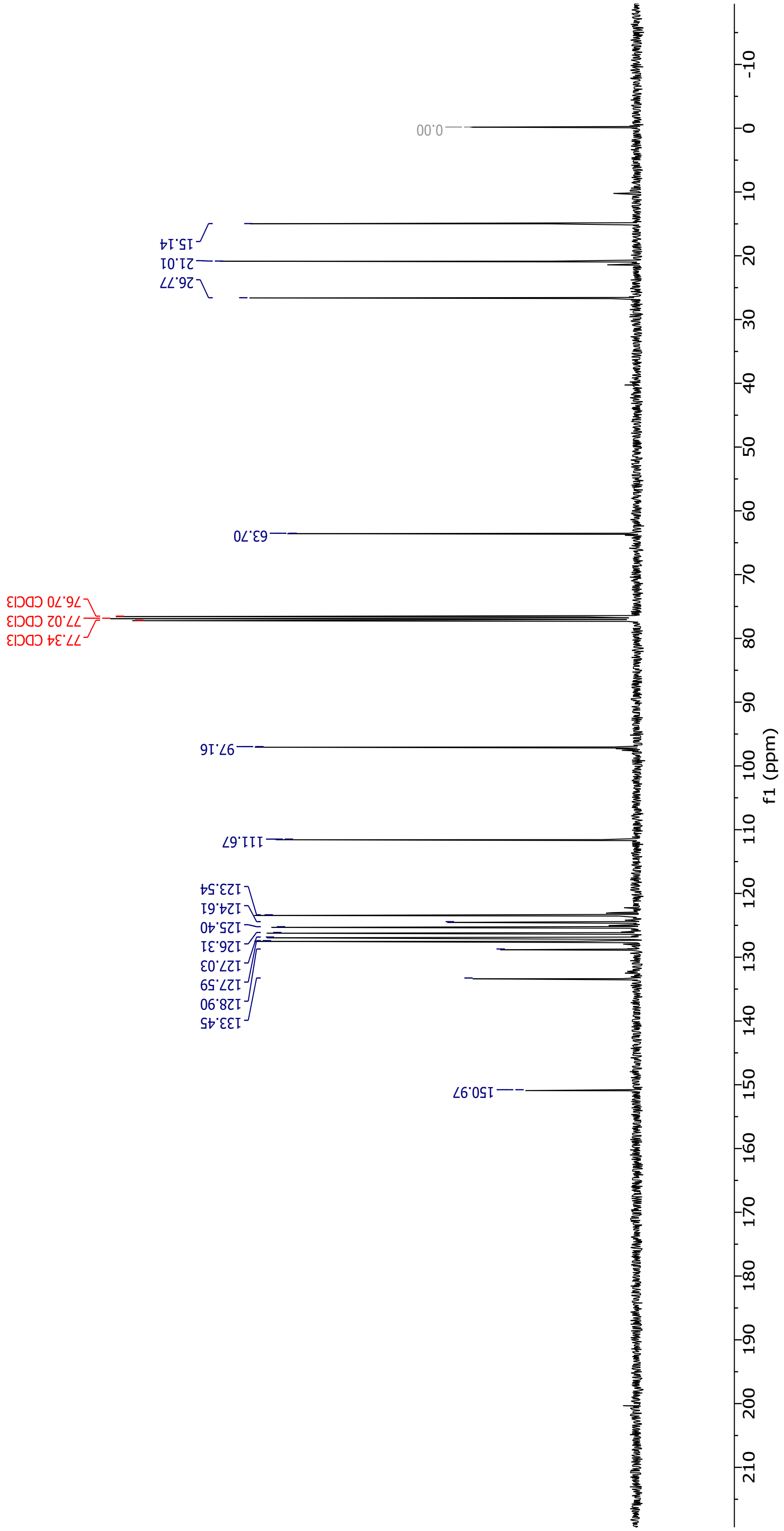

Supplement: Supplementary file 1 [file jo5c01997_si_001.pdf]
